# A human endothelial and adipose stem cell-based co-culture model for venous malformations

**DOI:** 10.1007/s10456-026-10045-9

**Published:** 2026-05-03

**Authors:** Mohammadhassan Ansarizadeh, Bojana Lazovic, Zahra Sarmadian, Abhishek Singh, Ryan Hicks, Lauri Eklund

**Affiliations:** 1 Oulu Center for Cell-Matrix Research, Biocenter Oulu and Faculty of Biochemistry and Molecular Medicine, University of Oulu, P.O. Box 5000, 90014 Oulu, Finland; 2 BioPharmaceuticals R&D Cell Therapy Department, Research and Early Development, Cardiovascular, Renal, and Metabolism (CVRM), BioPharmaceuticals R&D, AstraZeneca, Gothenburg, Sweden; 3 Translational Genomics, Discovery Sciences, BioPharmaceuticals R&D, AstraZeneca, Gothenburg, Sweden; 4 School of Cardiovascular and Metabolic Medicine and Sciences, King’s College London, London, UK

**Keywords:** Venous malformation, TIE2, Endothelial cell, Adipose tissue-derived stem cell, Vascular smooth muscle cell, *In vitro* disease model

## Abstract

Venous malformations (VMs) are developmental defects in vascular morphogenesis characterized by enlarged vein-like channels and defective perivascular cell coverage. The majority of VMs are caused by somatic mutations in endothelial cell (EC) receptor tyrosine kinase TIE2, but current medicinal options are limited. The common bottleneck when testing new therapeutic strategies and identifying efficient drug candidates is the absence of a reliable and robust model for replicating the characteristics of VMs. We report here on an advanced in vitro model for recapitulating cellular and molecular pathologies of the VM-causative *TIE2*^L914F^ mutation by co-culturing human adipose tissue-derived stem cells (hASCs) with either human umbilical vein ECs (HUVECs) retrovirally transduced to express *TIE2*^L914F^ or with induced pluripotent stem cell-derived ECs (iECs) with locus-targeted *TIE2*^L914F^. For comparison, cellular and molecular crosstalk between vascular cell types was investigated in HUVEC and primary vascular smooth muscle cell (vSMC) co-cultures. Advanced microscopy and transcriptomic analysis were used to investigate the cellular and molecular phenotypes in vascular cell types. Comparison of *TIE2*^WT^, gain- (*TIE2*^L914F^) and loss-of-function mutations (*TIE2*^KO^) revealed the importance of TIE2 signaling for vascular network formation in hASC/EC co-cultures. The model recapitulated the cellular hallmarks of the VMs and revealed gene signatures and signaling pathways relevant to VM pathologies. The data also demonstrated the utility of the hASC/EC model as a screening platform for VM pharmacotherapies. Overall, this work establishes a versatile platform for studying disease mechanisms affecting the vasculature and provides new insights into VM pathobiology and screening potential for therapeutic applications targeting VMs.

**Supplementary Information:**

The online version contains supplementary material available at 10.1007/s10456-026-10045-9.

## Introduction

Vascular anomalies (VAs) are disorders of blood or lymph vessels that can cause aesthetic problems, chronic pain, disability, organ dysfunction, or even sudden death [1, 2]. Venous malformations (VMs) are a common type of VA, often leading to aesthetic concerns and in severe cases to significant morbidity [3, 4]. VMs result from errors in vascular morphogenesis, forming dilated, thin-walled venous channels with uneven vascular smooth muscle cell (vSMC) coverage [5–9] and disorganization of the perivascular extracellular matrix (ECM) [10–12]. Genetic studies have revealed that most VMs are due to somatic gain-of-function mutations in the TIE2 gene (TEK) of endothelial cells (ECs) [10, 13, 14]. Invasive treatment options, including surgery and sclerotherapy, have limitations due to lesion inaccessibility and regrowth [15] and are unsuitable for patients with extensive or numerous VM lesions or lesions in vital organs. Since current molecular therapies face challenges concerning efficacy and side effects [16–19], more specific and effective therapeutic approaches for preventing VM growth or recurrence are highly desirable but currently lacking. One shortcoming in this field is a lack of robust model systems for mimicking the cellular and molecular characteristics of VMs and testing potential treatment strategies.

The commonly used in vitro tests are performed using monolayer cultures on plastic dishes or as 3D cultures of single EC types without vascular supportive cells (i.e. vSMCs or pericytes), but it is not possible to model the cellular architecture of vascular structures in monolayer cultures, and the presence of perivascular supportive cells would be necessary to replicate the VM pathology. Although some previous co-culture systems have used mesenchymal stem cells and HUVECs to promote EC network development, none has specifically focused on VM-causative TIE2 mutations [20–23]. The Transwell co-culture system has provided a setup for investigating the crosstalk between vSMCs and *TIE2*^L914F^ HUVECs [6] and has revealed a phenotypic transition in vSMCs in the presence of an inherited *TIE2*^R849W^ VM mutation [18]. Transwell studies are mainly limited to paracrine interactions, as the Transwell system does not allow free cell–cell contact between different cell types and may thus exclude contact-dependent signaling mechanisms. We have recently employed vessel-on-a-chip systems to investigate the mechanobiological responses of *TIE2*^L914F^ HUVECs and iECs under differential flow conditions [24, 25], but one shortcoming of microfluidic setups is that they commonly consist solely of EC monolayer and are based on fixed channels that do not allow the analysis of circumferential vascular growth or sprouting. Mouse models have offered insights into VM pathophysiology but are limited in scope, whereas human cell-based models are free from many of the ethical concerns that affect animal experiments and can be more relevant to drug development, since animals may not fully replicate human biology. To address these shortcomings of VM in vitro modeling, we report here on a co-culture set-up of HUVECs and adipose tissue-derived stem cells (hASCs). The latter include vascular supportive cell types that secrete a rich array of pro-angiogenic factors, including VEGF, FGF-2, PDGF, and TGF-β [26, 27]. These factors collectively stimulate EC migration and drive the formation of new vascular structures, making the co-culture conditions favorable for vessel development [27, 28]. The present hASC/HUVEC co-culture replicated cellular and molecular pathologies in VMs in terms of abnormalities in vascular morphogenesis and perivascular ECM, and in the interplay between EC and perivascular cells. To verify the results in retrovirally transduced *TIE2*^L914F^ primary ECs, we used iECs differentiated from pluripotent stem cells (iPS) with a locus-targeted *TIE2*^L914F^ mutation generated by CRISPR-Cas9. The hASC/HUVEC platform allowed testing of drug compounds to explore their efficacy against cellular abnormalities in VMs. Transcriptomic analysis provided insights into the effects of the *TIE2*^L914F^ mutation in ECs and in supportive vascular cell types and was used to verify the model by comparing in vitro and human VM tissue data.

## Results

### Generation and characterization of TIE2-mutated endothelial cells

Retroviral gene transfer was used to generate *TIE2*^L914F^-expressing primary HUVECs using a bicistronic IRES construct expressing green fluorescent protein (GFP) and TIE2 as separate proteins, as previously described [29]. HUVECs similarly overexpressing wild-type TIE2 (*TIE2*^WT^) or GFP without TIE2 overexpression (HUVEC^GFP^) were used as controls. The resulting cell lines were verified by western blot (WB) analysis and microscopic imaging (Fig. S1). *TIE2*^WT^ and *TIE2*^L914F^ were overexpressed at similar levels to HUVEC^GFP^, and the L914F mutation resulted in an increase in TIE2 phosphorylation and the activation of Akt downstream from *TIE2*^L914F^, indicating the expected gain-of-function effect.

**Fig. 1:**
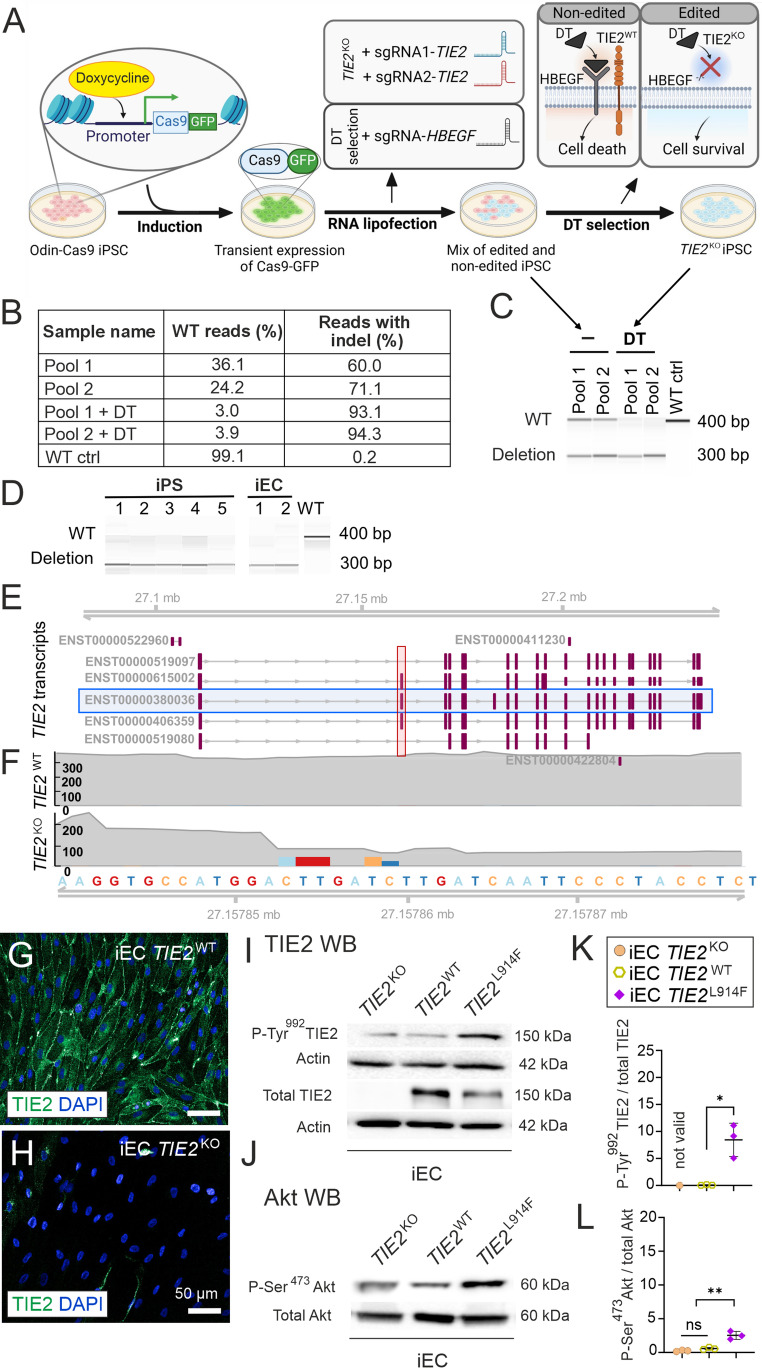
Generation and characterization of TIE2 deficient iECs using diphtheria toxin selection. **A** Stepwise overview of TIE2 deficient (*TIE2*^KO^) iEC generation. Step 1, induction of Cas9-GFP expression in OdinCas9-GFP iPSCs. Step 2, RNA lipofection to transfect sgRNAs targeting TIE2, and HBEGF for DT selection. Step 3, DT selection of edited cells. **B** Amplicon sequencing results showing edited pools before and after DT selection. **C** Fragment analyzer results displaying PCR products of the CRISPR/Cas9-targeted region before and after selection. **D** Fragment analyzer results demonstrate the stability of the targeted region over five passages in iPSCs and in two independent iEC differentiation batches. **E** Schematic representation of all known TIE2 transcripts, highlighting the most prominently transcribed isoform (blue horizontal rectangle) and the edited exon (red vertical rectangle). **F** RNA transcript analysis of *TIE2*^WT^ and *TIE2*^KO^ iECs with mutation sites indicated, showing transcript abundance in transcripts per million (TPM) on the left and the corresponding DNA codons with genomic locations in the bottom row. G and **H** Immunofluorescence staining of *TIE2*^WT^ and *TIE2*^KO^ iECs for TIE2. **I**, and **J** TIE2 and Akt protein expression and activation (phosphorylation states) investigated using Western blotting (WB) from cell lysates, confirming the absence of TIE2 in *TIE2*^KO^ iECs. VM-causative *TIE2*^L914F^ substitution results in increased TIE2 tyrosine phosphorylation (P-Tyr), indicating a gain-of-function effect. WB signal intensities are quantified on the left (**K** and **L**), *P < 0.05, **P < 0.01; ns, statistically non-significant in one way ANOVA followed by Tukey's post hoc test. n, three independent experiments. Means ± SD. Scale bar, 50 µm

For comparison to the gain-of-function *TIE2*^L914F^ mutation, we next generated TIE2-deficient (*TIE2*^KO^) iECs (Fig. 1A) using dual sgRNA CRISPR/Cas9 with diphtheria toxin (DT) selection in an inducible Cas9 iPSC line to improve efficiency [30]. RNA lipofection was used to deliver sgRNAs targeting TIE2 and the HBEGF receptor, enabling diphtheria toxin (DT) selection. Editing efficiency ranged from 60 to 71% (Fig. 1B) and increased to 93% after DT selection (Fig. 1C). Stability analysis across five passages showed a consistent *TIE2*^WT^/*TIE2*^KO^ ratio (Fig. 1D), suggesting no proliferative (dis)advantage. This ratio remained stable after differentiation into iECs, indicating that *TIE2* deletion does not affect the identity of the iEC. To confirm TIE2 loss-of-function, *TIE2* mRNAs were sequenced (Fig. 1E, F), revealing multiple non-sense substitutions and a significant reduction in its expression, which was corroborated at the protein level (Fig. 1G–L). The data verified effective and stable *TIE2* deficiency, validating the reliability of our CRISPR/Cas9 and DT selection strategy. The iEC *TIE2*^WT^ and iEC *TIE2*^L914F^ cell lines were generated as described previously [25]. Increases in the phosphorylation states of TIE2 and Akt were verified by western blotting (Fig. 1I–L). Taken together, *TIE2*^L914F^ increased TIE2 and Akt activation in both HUVECs and iECs. The activation state was higher in the retrovirally overexpressing HUVEC *TIE2*^L914F^ (Fig. S1) than in the locus-targeted iEC *TIE2*^L914F^.

### Enlarged EC structures, ECM deposition and perivascular cell abnormalities correlate with the state of TIE2 activation in the hASC/EC co-culture model

**Fig. 2:**
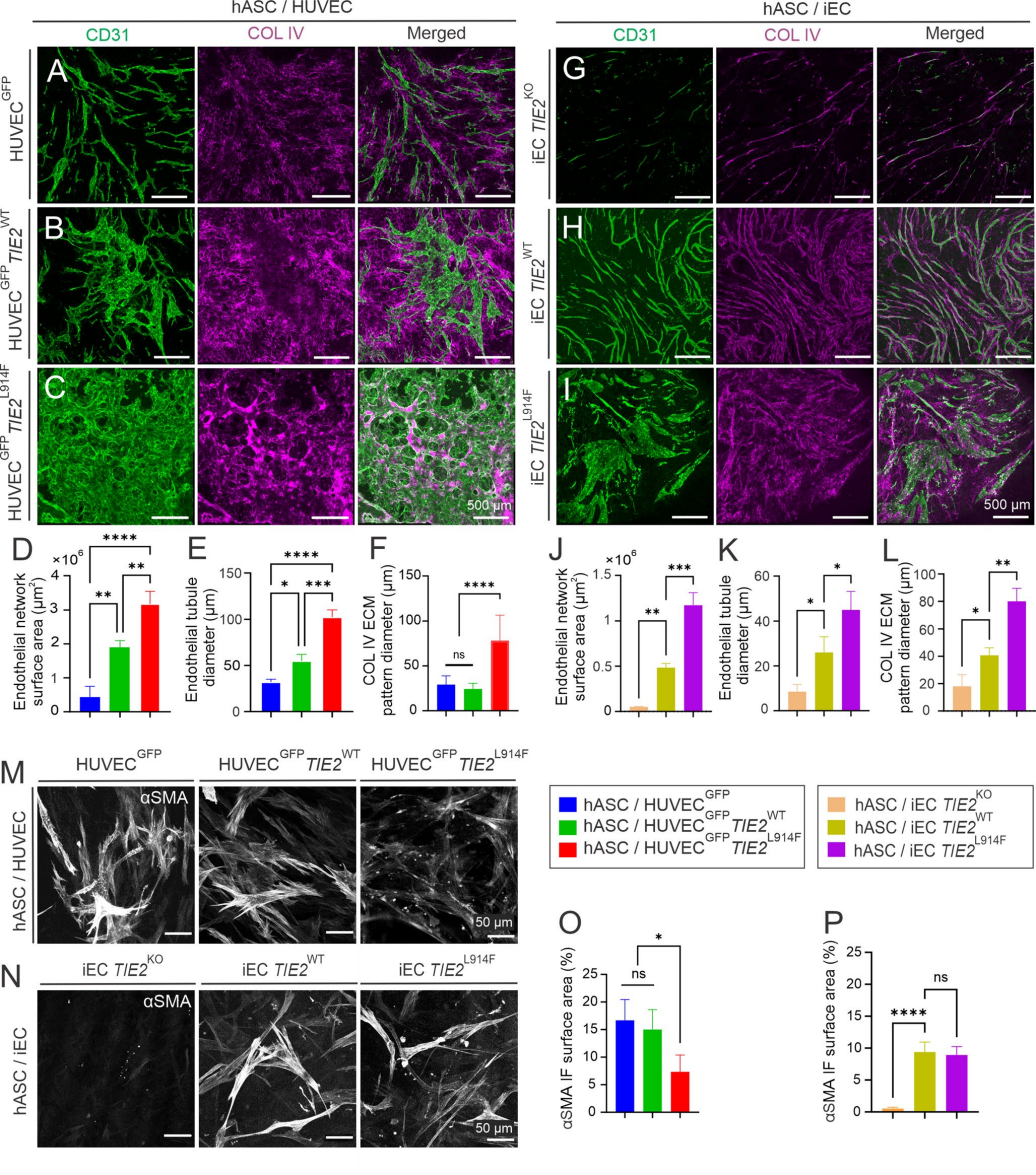
Co-cultures of human adipose tissue-derived stem cells with endothelial cells in fibrin gel. hASC/EC co-cultures were performed as described in ''Material and Methods'' section and stained with the antibodies indicated above the panels. **A** hASC/HUVEC^GFP^ formed a capillary-like EC network and **B** HUVEC^GFP^ *TIE2*^WT^ an intermediate type of capillary-like and expanded EC structures, while **C** HUVEC^GFP^ *TIE2*^L914F^ resulted in an expanded EC structure and no capillary-like architecture. **D**-**F** Quantification of the data, in which an increase in TIE2 signaling expands the EC network area, tubule diameter and COL IV deposition. **G** TIE2 loss-of-function (KO) mutation in iECs resulted in deficient formation of capillary-like structures. **H** iEC *TIE2*^WT^ formed capillary-like EC structures such as HUVEC^GFP^, and iEC *TIE2*^L914F^ resulted in abnormally enlarged EC structure. **J**-**L** Quantification of the data. **M** and **N** αSMA staining in co-culture with genetically edited HUVECs and iECs indicated. **O** and **P** Quantification of the αSMA immunofluorescence surface area. *P < 0.05, **P < 0.01, ***P < 0.001, ****P < 0.0001; ns, statistically non-significant in ANOVA followed by Tukey's post hoc test. n, three independent experiments. Means ± SD. Scale bar, 500 µm (**A**-**C**, **G**-**I**); 50 µm (**M**, **N**)

As VMs are deformations of vascular development, our interest was in developing an in vitro model allowing us to investigate the morphogenesis of the EC network. For this purpose, we co-cultured hASCs with HUVECs on fibrin gel using a published protocol [20]. Following a 7-day culture in vascular stimulation media, the control HUVEC^GFP^ formed an EC network resembling normal capillary structures (Fig. 2A), whereas the hASC/HUVEC^GFP^ *TIE2*^L914F^ exhibited abnormally enlarged EC clusters, without any organized capillary-like structure. HUVEC^GFP^* TIE2*^WT^ (overexpressing *TIE2*^WT^) resulted in an intermediate phenotype showing a capillary-like EC organization network in some areas and enlarged clustering in others (Fig. 2B, D–F). The negative control monocultures of HUVEC^GFP^ or hASCs did not form any cellular networks (Fig. S2).

For deeper insights into the EC network formation defect, hASC/HUVEC^GFP^ and hASC/HUVEC^GFP^ *TIE2*^L914F^ were imaged using time-lapse microscopy at 20 min intervals from day 0 until day 5 (Supplementary Movies 1 and 2, snapshots from selected time-points are shown in Fig. S3). The HUVEC^GFP^ cells adhered to each other, assembled themselves in an elongated, cord-like manner, showed sprouting activity, and formed a capillary-like lumenized structure, whereas their HUVEC^GFP^* TIE2*^L914F^ counterparts also adhered to each other and formed an elongated, cord-like structure, but afterwards grew into an abnormally large cell cluster having a distinct border (Fig. S3) and showing no sprouting activity or filopodia and were less polarized outwards from the EC cluster than the HUVEC^GFP^ cells (Fig. S4). Extended culture time somewhat increased the density of control EC network but did not result in any vessel-like structures in the mutants (Fig. S5). Histological analysis of VM biopsies from patients and mouse xenograft models have revealed abnormalities in vascular supportive tissue in VM lesions, including a wide, multilamellar basement membrane (BM), randomly distributed collagen fibrils and sparse vSMCs covering the affected vessels [10, 31]. BM-located type IV collagen (COL IV) is commonly considered to be deposited around ECs in the maturation phase of angiogenesis and less in the sprouting tips [32, 33]. Here COL IV staining followed the EC pattern in the control hASC/HUVEC^GFP^ samples but was more dispersed around the ECs in the HUVEC^GFP^*TIE2*^WT^ and unevenly deposited in the HUVEC^GFP^*TIE2*^L914F^ cases (Fig. 2A–C, F).

To verify the effect of *TIE2*^L914F^, we next investigated it in hASC/iEC co-cultures. In addition to providing another cellular source, iECs allowed us to compare the TIE2 gene locus-targeted *TIE2*^L914F^ gain-of-function mutation with a *TIE2-*deficient (*TIE2*^KO^) line. Here well-organized EC structures like HUVEC^GFP^ were formed in hASC/iEC *TIE2*^WT^ (expressing endogenous TIE2), with a well-defined COL IV matrix closely aligning EC tubules of a uniform diameter, while abnormally enlarged EC structures were observed in the case of iEC *TIE2*^L914F^, accompanied by disorganized COL IV. In contrast to the *TIE2*^WT^ and *TIE2*^L914F^ experiments, *TIE2*^KO^ iECs formed less numerous, and thinner EC tubules (Fig. 2G–I, J–L).

In a previous study of co-cultures of hASC with normal HUVECs [20] immunostaining showed the presence of platelet-derived growth factor receptor-β, αSMA and smooth muscle myosin heavy chain-positive cells, suggesting that hASCs can mature to vascular supportive cells of a contractile type (i.e. pericytes, SMCs). As VM affected vessels are characterized by sparse coverage of abnormal vSMCs [5–9], our interest was in investigating the phenotypes of αSMA^+^ cells in the presence of genotypically different ECs. Immunostaining showed a smaller area of αSMA in the presence of HUVEC^GFP^ *TIE2*^L914F^ than in the controls (Fig. 2M, O), while the αSMA staining was absent when iEC *TIE2*^KO^ was used (Fig. 2N, P). These data indicated that the extent of TIE2 signaling activity is critical for perivascular cell maturation in the hASC/EC model, as both loss (*TIE2*^KO^) and excessive activation (*TIE2*^L914F^) led to αSMC^+^ cell defects. There were also fewer cells expressing αSMA in the hASCs cultured with control iECs (Fig. 2P) than in those cultured with HUVECs (Fig. 2M–P). Furthermore, hASC/iEC *TIE2*^L914F^ did not result in αSMA^+^ cell defects (Fig. 2N, P), suggesting that the EC origin may affect maturation of hASCs towards the αSMA^+^ cell type. Altogether, these findings indicate that both balanced TIE2 signaling and the intrinsic properties of the EC types are essential for proper perivascular cell differentiation and vessel stabilization in an hASC/EC model in vitro.

### hASC/EC co-culture as a novel in vitro platform for testing VM pharmacotherapies

Although most VMs occur due to somatic gain-of-function mutations in TIE2, there are no specific TIE2 inhibitors available for clinical use. Instead of targeting TIE2, current molecular approaches used in clinics and/or in mouse models regarding TIE2-mutation-positive VMs have been based on the inhibition of multiple tyrosine kinases using the BCR-ABL inhibitor ponatinib, or have been targeted at PI3K/AKT downstream from the mutant TIE2 by means of rapamycin or alpelisib [34–42]. Mechanistically, rapamycin may downregulate mTORC2-mediated AKT_Ser473_ phosphorylation [43], while alpelisib is a specific inhibitor of the catalytic subunit of the PI3K.

**Fig. 3:**
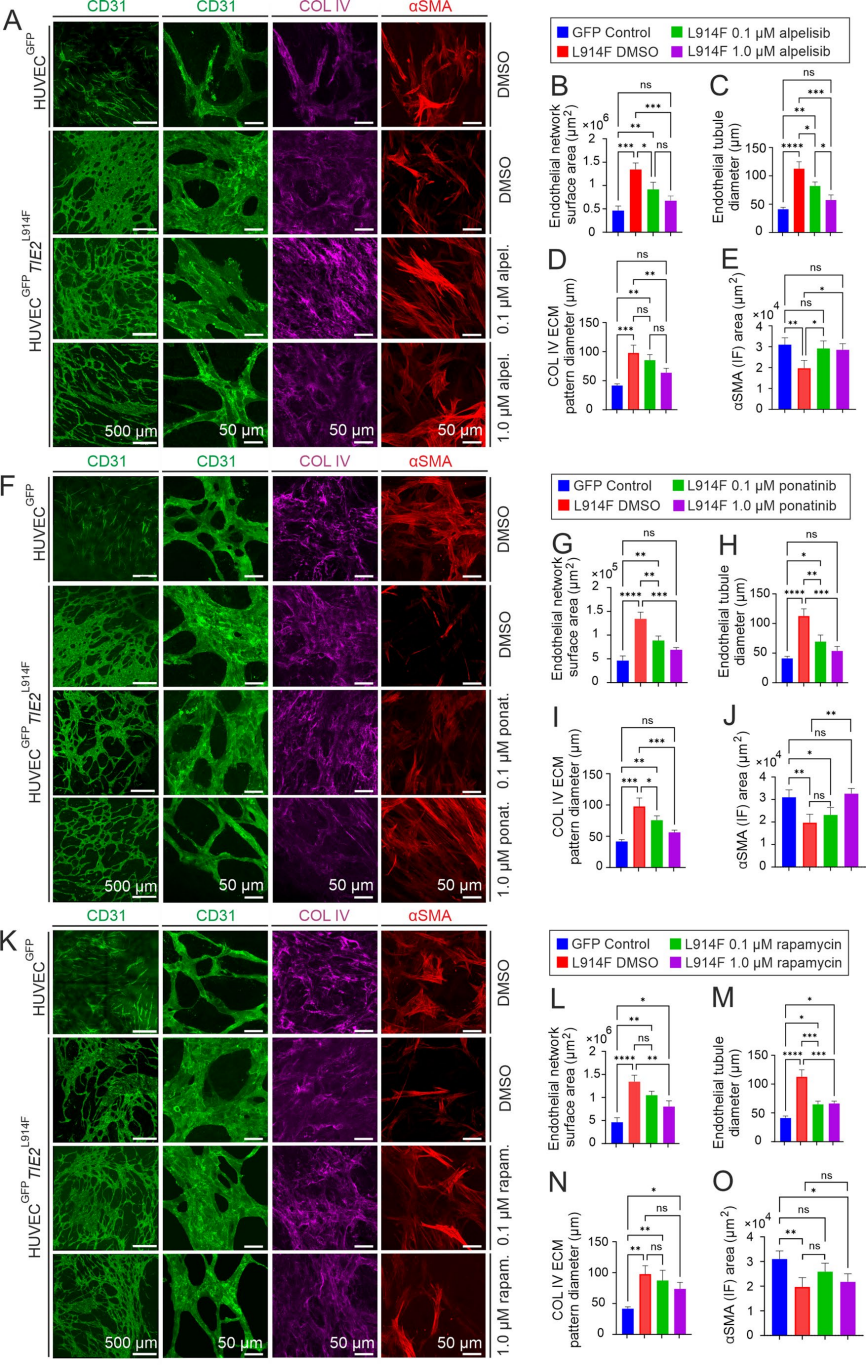
Efficacy of alpelisib, ponatinib and rapamycin for normalizing *TIE2*^L914F^ EC network formation defects in the hASC/HUVEC co-culture model. Cells (genotypes to the left of the image panels) were grown for 5 days, treated with DMSO (control) or with the inhibitor indicated (to the right of the image panels), fixed and stained with antibodies against CD31, COL IV and αSMA. Quantification of the image data is shown on the right. **A** and **B** 0.1 µM alpelisib (vs. DMSO-treated *TIE2*^L914F^) resulted in narrowing of the enlarged EC network, **C** tubule diameter, **D** COL IV pattern and **E** restored low αSMA area. Alpelisib at a 1.0 µM concentration resulted in normalization of the EC network and the COL IV and αSMA defects (i.e. no difference between *TIE2*^L914F^ and HUVEC^GFP^). **F** and **G** 0.1 µM ponatinib (vs. DMSO treated *TIE2*^L914F^) resulted in narrowing of the enlarged EC network, **H** tubule diameter, (**I**) COL IV deposition, and **J** restored αSMA area. A 1.0 µM concentration resulted in normalization of the EC network and αSMA defect (non-significant difference in ANOVA vs. HUVEC^GFP^). **K** and **L** 0.1 µM rapamycin (vs. DMSO-treated *TIE2*^L914F^) narrowed the EC tubule diameter, and a 1.0 µM concentration decreased EC surface area and the COL IV pattern diameter. **O** 1.0 µM rapamycin normalized αSMA expression (no difference between *TIE2*^L914F^ and HUVEC^GFP^), but not **L** the EC surface area, **M** tubule diameter or **N** COL IV pattern. *P < 0.05, **P < 0.01, ***P < 0.001, ****P < 0.0001; ns, statistically not significant in ANOVA followed by Tukey's post hoc test. n, three independent experiments. Scale bar, 500 µm (CD31 left), others 50 µm. Means ± SD

As proof-of-concept, the feasibility of hASC/EC setting for testing therapeutics against VMs was demonstrated by culturing hASC/HUVEC^GFP^ *TIE2*^L914F^ in the absence or presence of the above inhibitors and comparing the effects with those of carrier-treated (DMSO) *TIE2*^L914F^ and with healthy controls (hASC/HUVEC^GFP^) in terms of EC network formation, COL IV pattern and αSMA^+^ expression. Treatment with 0.1 µM alpelisib (Fig. 3A) and ponatinib (Fig. 3B) narrowed the *TIE2*^L914F^ network, while normalization (i.e. a statistically non-significant difference from DMSO-treated HUVEC^GFP^) was achieved with 1.0 µM of ponatinib and alpelisib. Rapamycin reduced the diameter of the enlarged *TIE2*^L914F^ EC structures and increased the αSMA area but was less efficient than ponatinib and alpelisib in normalizing EC network and αSMA defects. A higher concentration of all three inhibitors (10.0 µM) resulted in toxicity (fragmented EC structures and cell death).

### Transcriptomic analysis of the hASC/HUVEC VM model

**Fig. 4:**
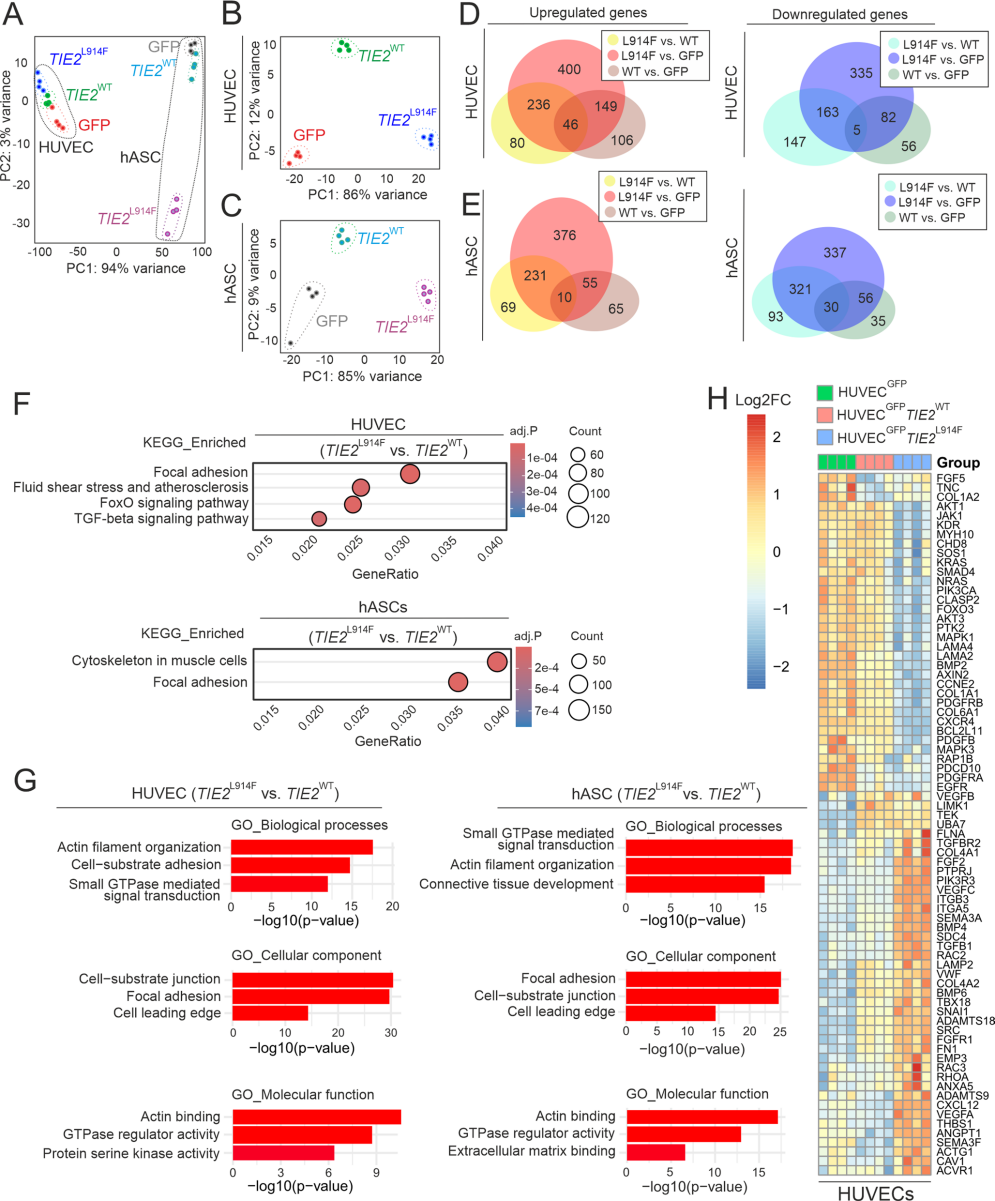
Transcriptomic analysis of hASC/HUVEC co-cultures. HUVECs were retrovirally transduced with bicistronic TIE2-IRES-GFP or GFP-expressing retroviruses and co-cultured with hASCs for 7 days. HUVECs (GFP^+^) and hASCs (GFP^-^) were separated by FACS and transcriptomic analysis was performed as indicated in Material and Methods. **A**-**C** Principal component analysis. Note the distinct clustering of genotypically different HUVECs and co-cultured hASCs. **D** and **E** Venn diagrams of the top 1000 up- and downregulated genes. Genes with adj.*P* < 0.05; Log2FC < –1 were classified as downregulated, while those with adj.*P* < 0.05; Log2FC > 1 were classified as upregulated. **F** Selected dot plots of the top 20 enrich pathways in hASC and in HUVECs (*TIE2*^L914F^ vs. *TIE2*^WT^) potentially relevant to vascular anomalies. **G** Selected bar plots of the top 20 GO enrichment analyses for biological processes, cellular components and molecular functions in hASCs and HUVECs. All P-values < 1×10^-9^. The full GO and KEGG tables are shown in the supplementary material. **H** Heatmap of individual DEGs in HUVECs. The DEGs were selected based on the GO and KEGG analyses, potential involvement in VMs and biological function in ECs based on published data

Xenografts with VM mutations expressing HUVECs and the use of EC-specific Cre drivers together with inducible VM mutations have indicated that a somatic mutation in an EC compartment is sufficient for the formation of VM lesions [10, 43–46]. This suggests that vSMC abnormality results secondarily to the alteration in signaling from EC to SMC, as vSMCs themselves do not harbor the mutation. To investigate cellular phenotypes and the interaction between hASCs and *TIE2*^L914F^ HUVECs in more depth, we next analyzed hASC/HUVEC co-culture using transcriptomic analysis (Fig. 4). After 7 days of co-culture, the cells were trypsinized and the hASCs and HUVECs were sorted out using FACS based on GFP expression in the HUVECs (Fig. S6A). Genes selectively expressed in HUVECs (including ANGPT2, CD31, CHD5, TEK and TIE1) were not enriched in the hASCs population (Supplementary_DEGs.xlsx) verifying the successful separation of the hASCs from the HUVECs, as was also evident in principal component analysis (PCA, Fig. 4A). Different HUVEC genotypes also exhibited distinct gene expression profiles in the PCA (Fig. 4B) and volcano plot (Fig. S7A). Interestingly, hASCs co-cultured with different HUVEC genotypes also displayed distinct clustering (Fig. 4C, S7B), indicating a significant influence of TIE2 signaling in the ECs on the co-cultured hASCs. As indicated in the Venn diagrams and Volcano plots (Fig. 4D, E, S7), the highest number of differentially expressed genes (DEGs) were detected in the comparison of *TIE2*^L914F^ with GFP in both the hASCs and HUVECs. In view of the cellular phenotypes observed in the hASC/HUVEC co-cultures and in VM lesions [9, 13, 14, 47], we were especially interested in DEGs related to cell signaling, ECM, the actin cytoskeleton, shear stress sensing and mural cell maturation. Interestingly, these were included in the top 20 pathways in the Kyoto Encyclopedia of Genes and Genomes (KEGG) and in Gene ontology (GO) pathway enrichment analysis (Fig. 4F–H, S8, Supplementary pathway enrichment analysis).

### Phenotypic analysis of vascular smooth muscle cells in vSMC/HUVEC co-cultures

**Fig. 5:**
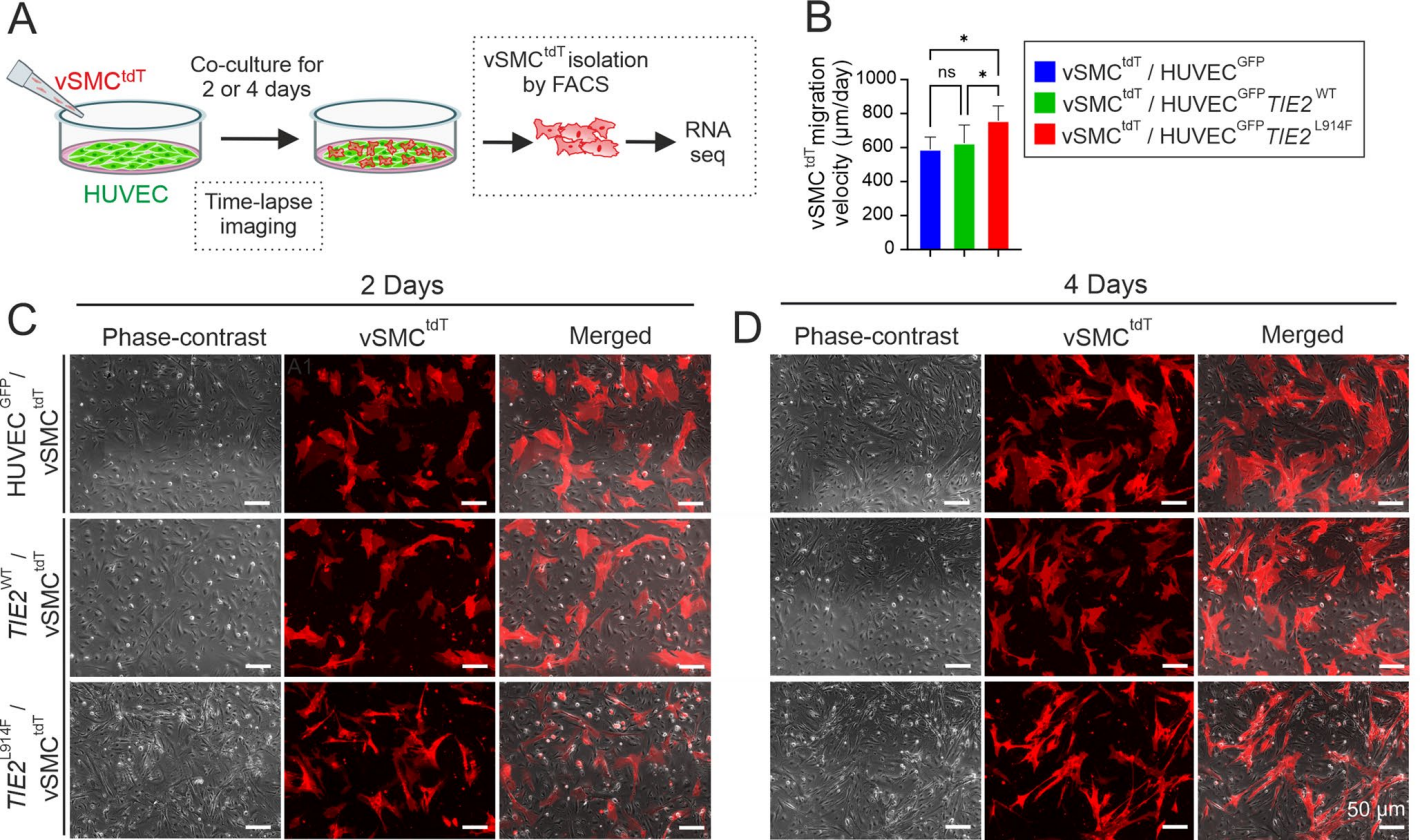
vSMC^tdT^/HUVEC co-cultures. **A** Experimental scheme. Three genotypes of HUVECs, (HUVEC^GFP^, HUVEC^GFP^ *TIE2*^WT^ and HUVEC^GFP^ *TIE2*^L914F^), were cultured for 2 days after seeding vSMC^tdTomato^ (vSMC^tdT^) on top of the HUVEC layer, time-lapse imaged, or FACS sorted followed by RNA extraction and sequencing. **B** Migration velocity as quantified from time-lapse movies using a cell tracker. Means ± SD. *P < 0.05; ns, statistically non-significant in one way ANOVA followed by Tukey's post hoc test. n, three independent experiments. **C** and **D** Cellular morphology of vSMCs^tdT^ in co-cultures of HUVEC and vSMC imaged after 24 h and 48 h. Genotypes of HUVECs are indicated on the left. The vSMCs^tdT^ have a more elongated and narrower phenotype when cultured on top of HUVEC^GFP^ *TIE2*^L914F^. Scale bar, 50 µm

Although vSMCs are irregularly distributed around the affected vessels in VMs, the mechanisms resulting in defective vSMC coverage and how these may contribute to the formation of VM lesions are not known. Our results obtained with hASC/HUVEC setups showed that *TIE2*^L914F^ interferes with the differentiation of αSMA^+^ cells from hASC population. For comparison to hASC/EC model, which may mimic developmental angiogenesis, we next investigated matured primary vein SMCs transduced with a fluorescent marker protein tdTomato (vSMC^tdT^) in co-cultures with either HUVEC^GFP^, HUVEC^GFP^ *TIE2*^WT^ or HUVEC^GFP^ *TIE2*^L914F^ (Experimental scheme in Fig. 5A). The cellular phenotypes were investigated by fluorescence microscopy and transcriptional analysis on days 2 and 4 (Fig. 5C, D). The phenotype of vSMCs^tdT^ on top of HUVEC^GFP^ and HUVEC^GFP^ *TIE2*^WT^ were mutually similar, while vSMCs^tdT^ co-cultured with HUVEC^GFP^ *TIE2*^L914F^ were elongated and narrower by comparison with the control HUVEC lines. Cell tracking measurements from the time-lapse movies showed that vSMCs^tdT^ were more motile in the presence of HUVEC^GFP^ *TIE2*^L914F^ than the controls HUVEC^GFP^ or HUVEC^GFP^* TIE2*^WT^ (Fig. 5B).

**Fig. 6:**
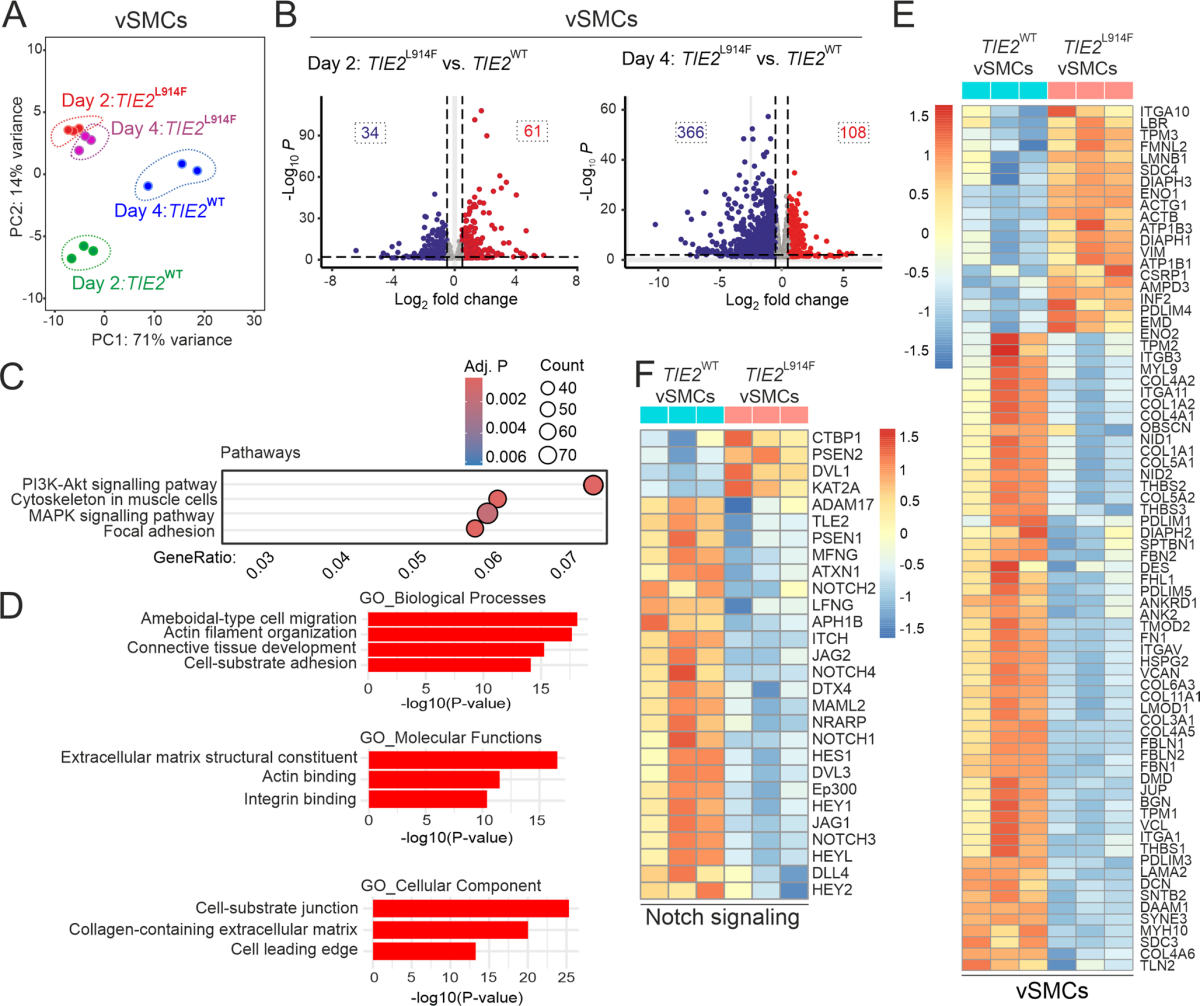
Transcriptomic analysis of vSMC/HUVEC co-cultures. **A** The PCA plot illustrates the distinct clustering of vSMCs co-cultured with the TIE2 genotypes indicated and harvested on days 2 and 4. **B** The volcano plots highlight the differential expression of vSMCs genes on days 2 and 4. The DEGs in blue/red are down/upregulated and the number of DEGs in each case is in dashed boxes. **C** Dot plot of potentially VM-related pathways among the top 20 KEGG-enriched pathways (vSMCs co-cultured with *TIE2*^L914F^ vs. *TIE2*^WT^, at day 2). **D** Bar plot of GO enrichment analyses highlighting potentially VM-related pathways among the top 20 GOs (vSMCs co-cultured with *TIE2*^L914F^ vs. *TIE2*^WT^ at day 2). All P-values <1×10^-6^. The full GO and KEGG tables are shown in the supplementary data. **E** Heatmap of DEGs involved in vSMC contraction, ECM, cytoskeleton and **F** Notch signaling (at day 4)

To complement our microscopic observations of elongated migratory vSMCs, we performed a transcriptomic analysis to investigate associated changes in gene expression. vSMCs^tdT^ for RNAseq were harvested on days 2 and 4 and selected by FACS (tdT^+^; GFP^−^) (Fig. S6B). PCA plots showed vSMCs^tdT^ co-cultured with HUVEC^GFP^ *TIE2*^L914F^ to exhibit complete segregation from vSMCs^tdT^ co-cultured with HUVEC^GFP^ *TIE2*^WT^ (Fig. 6A), and also demonstrated differential clustering of vSMCs cultured on *TIE2*^WT^ for 2 or 4 days, while this difference in timepoints was not observed in the vSMCs/*TIE2*^L914F^ co-cultures (Fig. 6A). Following filtering based on an adj.*P* < 0.05, we identified 1787 DEGs on day 2 and 5657 on day 4 in *TIE2*^WT^ relative to *TIE2*^L914F^ (Fig. 6B). To pinpoint the biological pathways which such DEGs may influence, a KEGG pathway enrichment analysis was performed based on the day 2 dataset, to find early rather than secondary responses. The top four enriched pathways with the highest gene counts are shown in Fig. 6C and the top twenty KEGG-enriched pathways in Supplementary_Pathway enrichment analysis. Among the top twenty GOs, the pathways relevant to vSMC defects in VAs, or other forms of vSMC disease were selected based on published data [6, 31, 48–50] and presented in Fig. 6D. DEGs within in these GO pathways included genes linked to the regulation of cell signaling, cell migration, the SMC cytoskeleton, ECM, and Notch signaling (Fig. 6F, E, S8).

### Comparison of the transcriptomic data between hASC/HUVEC co-cultures, HUVEC monocultures and TIE2 mutation-positive VM lesions

To further examine the relevance of the hASC/EC model and the DEGs identified by it, we utilized the previously published, openly available RNAseq data sets from TIE2 mutation-positive EC monocultures [51] and VM patients [7]. The previous comparison of *TIE2*^WT^ with *TIE2*^L914F^ in HUVEC monocultures identified 80 genes with statistically significant changes of at least twofold in their expression (34 genes upregulated and 46 downregulated), and 56 out of these 80 genes showed a change in the same direction (up or down) also in the hASC/HUVEC co-culture, although the magnitude was statistically different in 38 cases. 8 DEGs upregulated in the monoculture were downregulated in the co-culture, and 15 DEGs downregulated in the monoculture were upregulated in the co-culture (Supplementary_DEGs.xlsx).

Our reference to human VMs consisted of data from a recent study [7] that included the largest dataset of TIE2 mutation-positive VM patient samples available to date. Here, 80 out of 190 upregulated genes identified in VM lesions were also upregulated in a hASC/HUVEC setup (*TIE2*^L914F^ vs. *TIE2*^WT^). Given the possibility of an artefact due to overexpression of the TIE2 in HUVECs, we also included DEGs from iECs (*TIE2*^L914F^ vs. *TIE2*^WT^) and found thirty-three mutual genes upregulated in all three datasets (Supplementary_DEGs.xlsx). After performing pathway enrichment analysis (Fig. S8) on these thirty-three genes using Metascape (https://metascape.org [52]), the vascular lesion pathway emerged as the top hit in the DisGeNET enrichment results, indicating a good level of similarity between the patient and the in vitro transcriptomic data.

## Discussion

VMs are the most common type of vascular malformation. While the genetic cause of most VMs is known, the molecular and cellular changes resulting in VM growth are still incompletely understood. From a clinical point of view, efficient treatment options are still limited in many VM cases, so that the search for new therapeutic strategies is necessary to address clinical need. Since the isolation and expansion of ECs expressing the TIE2 mutant and lesion-associated vSMCs from low proliferating VMs has proved technically challenging, patient-derived cells have not been available for experimental applications and there have been no data available for vSMCs isolated from VM lesions. Thus, in vitro models using genetically modified human cells are a matter of high priority. In this work we developed a novel co-culture model that replicated cellular and molecular VM pathologies and allowed cell type-specific high-resolution microscopic analysis providing quantifiable phenotypic and molecular disease read-outs. Transcriptomic analyses of co-cultured endothelial and supportive vascular cell types revealed previously unreported gene signatures associated with VM pathophysiology and allowed us to investigate crosstalk between mutant ECs and vascular supportive cells and examine the phenotypic changes in both cell types. To demonstrate the feasibility of drug testing, we performed a proof-of-concept experiment using potential inhibitors of VMs. Finally, we also tested the disease relevance of the model by comparing in vitro readouts with clinical data for TIE2 mutation-positive VMs.

Our cellular source for ECs was primary cells (HUVECs) genetically engineered through retroviral transduction to express the VM-causative mutation *TIE2*^L914F^ and the selection marker gene GFP. For comparison with primary ECs, we used iECs differentiated from iPSCs expressing a TIE2 gene locus-targeted L914F mutation. We also successfully generated a TIE2-deficient iPSC line (*TIE2*^KO^) using dual-sgRNA CRISPR/Cas9 and diphtheria toxin selection, resulting in a stable iEC *TIE2*^KO^ population, which demonstrated the indispensable role of TIE2 in forming vessel-like EC structures in hASC/EC co-cultures. In conventional 2D monocultures the iEC *TIE2*^KO^ maintained endothelial identity without any apparent proliferative disadvantage or phenotypic drift across passages, demonstrating that TIE2 is dispensable for iEC differentiation and maintenance under basal conditions, a result consistent with conventional [53] and induced TIE2 deletion in mice [54]. In contrast to the thin, sparse cellular architecture formed by TIE2-deficient ECs, *TIE2*^L914F^ resulted in abnormally enlarged EC structures, which is in agreement with the gain-of-function effect of the L914F mutation. In addition to the abnormally widened EC structure, microscopic analysis revealed a low abundance of the cellular characteristics of spouting activity (i.e. filopodia and polarized cells) in the presence of *TIE2*^L914F^. This may contribute to the formation of larger cell clusters instead of the network architecture observed in the control ECs. In the tip cells which guide the outgrowth of vascular sprouts, TIE2 is expressed at low levels in vivo [55, 56] and it is plausible that high TIE2 signaling may interfere with tip cell identity. Golgi polarization is associated with EC migration, endothelial lumen formation and vessel specification [57, 58]. We and others have previously reported that ANGPT1/*TIE2*^WT^ promotes front-rear polarity in motile ECs [59] and that HUVEC *TIE2*^L914F^ show less frequently a polarized phenotype that normal ECs acquire in migration, in lumen formation [60], and as a response to laminar shear stress [24]. The *TIE2*^L914F^ ECs in the hASC/HUVEC co-cultures were less polarized at the front of the cell clusters, which may contribute to the inward migration pattern and result in the formation of large EC structures rather than sprouts.

In addition to our microscopic investigation of cellular phenotypes, transcriptomic analysis revealed significant alterations in gene expression profiles in both EC compartments and in hASCs or vSMCs co-cultured with HUVEC^GFP^ *TIE2*^L914F^. KEGG and GO enrichment analysis of DEGs in the hASC/HUVEC model identified the EC pathways involved in cell-to-ECM interactions, the actin cytoskeleton and cellular signaling. KEGG enrichment analysis of the DEGs in hASCs in co-culture with HUVEC *TIE2*^L914F^ showed major changes in cytoskeletal and ECM-related pathways, and GO enrichment analysis identified changes in GTPase signaling, the actin cytoskeleton, connective tissue development, cell-to-ECM adhesion, and ECM organization. Altogether, the data indicated that mutant ECs significantly influence the perivascular cells that may occur in a VM microenvironment via changes in the vascular ECM, secreted growth factors, and direct cell–cell contacts.

The hASC/HUVEC model may mimic developmental angiogenesis in terms of EC-supportive vascular cell interactions, tube formation and vessel network morphogenesis, although the exact cellular identity of the αSMA^+^ cells remained elusive. Our interest was therefore in the investigation of the mutant EC-to-vSMCs signaling in fully differentiated primary vein SMCs. In our experimental setup the vSMCs^tdT^ plated on top of the HUVEC^GFP^* TIE2*^L914F^ were more elongated than those grown on top of the control ECs (HUVEC^GFP^ or HUVEC^GFP^ *TIE2*^WT^) and showed enhanced motility. To assess the vSMC phenotypic changes in more depth, we performed transcriptomic profiling at two time points, on days 2 and 4. We were especially interested in the DEGs involved in cell signaling, perivascular ECM deposition and SMC contraction, i.e. those previously implicated in VMs [5–12] and known to be essential for vascular integrity and functionality [61, 62]. KEGG and GO pathway enrichment analysis of RNA-seq data revealed changes in PI3K-Akt, TGFβ and MAPK signaling, as well as in the SMC cytoskeleton, ECM and cell adhesion. We also compared our data with a recent vSMC/HUVEC co-culture study performed in a Transwell set-up in the presence of the inherited VM mutation *TIE2*^R849W^ [31]. Both the *TIE2*^R849W^ and the *TIE2*^L914F^ datasets showed significant downregulation of canonical contractile cell markers such as ACTA2 and CNN1, suggesting a shift from a contractile towards a synthetic phenotype, although this did not hold true for the other markers of a synthetic phenotype in *TIE2*^L914F^ (e.g. TAGLN, OPN). Overall, our findings indicate that the expression of *TIE2*^L914F^ in ECs affects the vSMC genes involved in contraction and tissue remodeling, even though the resulting vSMC characteristics do not fit the previously suggested canonical contractile / synthetic vSMC phenotypes.

To investigate whether *TIE2*^L914F^ results in different transcriptome readouts in co-culture from monoculture we compared the hASC/EC transcriptomic data with previous findings in a HUVEC *TIE2*^L914F^ monoculture [63]. The same DEGs were also identified in the co-culture, but the level of expression was statistically different in 75% of cases, indicating that co-cultured hASCs may influence the effect of *TIE2*^L914F^ on ECs. To examine the relevance of the hASC/HUVEC model to clinical VM research, we compared our in vitro results with recently published human VM tissue data [7]. Although the current limitation is that human data do not include downregulated genes, 80 out of the 190 upregulated genes identified in VM lesions, were also upregulated in the hASC/HUVEC experimental design. As a summary, the most potentially VM-related DEGs identified in ECs, hASCs and vSMCs based on our in vitro study, determined by GO and KEGG enrichment analyses, are visualized in Fig. S9.

The high potential of hASCs to support angiogenesis is because they origin from vascular cell types. Adipose tissue is composed of adipocytes and non-adipocyte stromal-vascular fraction. In this study, we used hASCs isolated from the stromal fraction by mechanical dissociation, enzymatic digestion and subsequent culturing of adherent cells. As previously characterized, most of the hASCs express mesenchymal stem cell markers (CD73, CD90, CD105) [20] and they show multilineage differentiation capacity [64]. Interestingly, in adipose tissue hASCs locate in perivascular niche [65], and perivascularly located mesenchymal progenitor and pericytic cells have been identified in other tissues as well [66, 67]. Accordingly, FACS assays of the pooled hASC populations have revealed the presence of pericyte (NG2, 31.2% of cells; CD140b, 52.8%) and perivascular stem cell marker expressing cells (CD34, 40.9%) [20]. The cellular heterogeneity has been further studied by single-cell RNAseq of cultured hASCs [68] and freshly sorted stromal vascular fraction [69]. These studies have identified at least two major hASCs subpopulations; contractile type of vascular cells (i.e. pericytes and/or vSMCs) and less differentiated adventitial stromal cells. Collectively these cell types can support EC structures directly, by secreting proangiogenic factors and by providing an ECM scaffold for vascular structure [26, 70, 71].

Standard VM treatments, compression, surgery and sclerotherapy provide symptom relief but are rarely curative, and due to regrowth, patients may require lifelong interventions. Novel cellular and molecular therapies offer the promise of new treatment strategies to combat VMs, but their development requires reliable disease models. To demonstrate the potential of the hASC/HUVEC co-culture approach for drug development, we investigated the effect of three inhibitors that have been shown to downregulate TIE2 mutation-positive VM signaling pathways in vitro [43, 72, 73] and also to have some efficacy against VMs in mice and patients [42, 43, 73]. Rapamycin, primarily known for its immunosuppressant action, downregulates mTORC2-Akt_Ser473_ signaling downstream from TIE2 and may thus provide clinical benefits but is not curative [34–37] and had a limited effect in hASC/HUVEC co-cultures as it did not normalize all the cellular *TIE2*^L914F^ abnormalities. Alpelisib is a specific PIK3CA inhibitor for PIK3CA-related overgrowth syndromes and it is also FDA-approved for the management of the subset of vascular malformations associated with PIK3CA-related overgrowth spectrum [38–40]. It downregulates PIK3/Akt signaling more efficiently than rapamycin in in vitro experiments [72], and in preliminary clinical tests it has shown effectiveness against TIE2-mutant VM [38, 40–42], and also normalized cellular *TIE2*^L914F^ abnormalities in the hASC/HUVEC model. Ponatinib, a multi-target tyrosine-kinase inhibitor, regressed *TIE2*^L914F^ VMs in a mouse transplantation model [73] and similarly normalized cellular *TIE2*^L914F^ abnormalities in hASC/HUVEC co-cultures. These results show that hASC/HUVEC provides a potential platform for drug testing.

All in all, our findings demonstrate the feasibility of hASC/HUVEC co-culture as a disease model for VMs, providing quantitative measures for evaluating the pathogenicity of a gene variant, and readouts for investigating drug efficacy. We also identified vascular cell-type specific effects of the *TIE2*^L914F^ VM mutation and hASC/HUVEC showed a good level of similarity to patient data by means of a comparative transcriptomic analysis, indicating its potential as a disease model. The DEGs in all three experimental settings (HUVECs, iECs, and patient data) may represent potential biomarkers or therapeutic targets with respect to VMs, thus warranting further investigation. However, despite these advantages, the hASC/EC model has certain limitations. First, it lacks perfusion through the vascular networks, which is a matter of physiological relevance [74, 75] and also having a role in VMs [24]. Second, the hASC/HUVEC setting does not replicate the vessel type-specific features of venous development and maturation. Third, although the ECs in the co-culture with hASCs form a capillary-like architecture with some lumen structures, the experimental design is not a true 3D angiogenic assay, as the EC structures grow on the top of the hASCs and not freely in a 3D space. Addressing these limitations in future studies could help develop a more pathophysiologically representative and comprehensive *in vitro* model of VMs.

## Material and methods

### Generation of ***TIE2***^L914F^ and ***TIE2***^KO^ iPSC lines

iEC *TIE2*^WT^ and iEC *TIE2*^L914F^ were generated, differentiated and cultured as described in our previous study [25]. To generate *TIE2*^KO^ iPSCs, OdinCas9-hiPSCs were seeded at a density of 40,000 cells/cm^2^ in 6-well plates 36 h before transfection and Cas9 expression was induced 16 h prior to transfection by treating the cells with 10 µg/ml doxycycline (Sigma, TA9H11E417A3) for 1 h. A few hours before transfection the culture medium was refreshed to increase the likelihood of cells being in the proliferative log phase. The iPSCs were transfected using the CRISPRMAX reagent (Thermo Fisher Scientific, CMAX00003) with a 2.5:1 transfection reagent to sgRNA ratio, following a reverse transfection protocol. For transfection, 40,000 cells/cm^2^ were directly seeded into a 96-well format onto pre-formed transfection complexes containing 60 ng of sgRNA per well, targeting TIE2 and HBEGF for co-selection. Transfected iPSCs were treated with 20 ng/ml diphtheria toxin (DT) starting from day 3 post-transfection. The DT-supplemented medium was then replaced daily until the negative control cells died. sgRNA sequences were designed to target the first exon transcribed in all mRNA variants of the targeted genes (Table S1) and were synthesized by Synthego.

### Genomic DNA extraction and next-generation amplicon sequencing

Genomic DNA was extracted using the QuickExtract DNA Extraction Solution (Lucigen, NC9904870) following the manufacturer’s instructions. Amplicons of interest were analyzed from genomic DNA samples using a NextSeq platform (Illumina). Briefly, target genomic regions were amplified in a two-step PCR process. The first PCR was performed using the NEBNext Q5 Hot Start HiFi PCR Master Mix (New England Biolabs) in 15 μl reactions, with 0.5 μM of primers (Table S2) and 1.5 μl of genomic DNA as a template. The cycling conditions were as follows: 98 °C for 30 s, followed by 30 cycles of:98 °C for 10 s, primer-specific annealing temperature for 20 s and 72 °C for 20 s, with a final extension at 72 °C for 2 min. PCR products were purified using the HighPre PCR Clean-up System (MagBio Genomics), and the correct product size and DNA concentration were verified using a Fragment Analyzer (Agilent).

In the second PCR step unique Illumina indices were added using the KAPA HiFi HotStart Ready Mix (Roche, KK2601). A total of 1 ng of purified first-round PCR product was used as a template in a 50 μl reaction containing indexing primers. The cycling conditions for this step were: 72 °C for 3 min, 98 °C for 30 s, followed by 10 cycles of 98 °C for 10 s, 63 °C for 30 s, and 72 °C for 3 min, with a final extension at 72 °C for 5 min. The final PCR products were purified using the HighPre PCR Clean-up System (MagBio Genomics, AC-60005) and analyzed using a Fragment Analyzer (Agilent). Libraries were quantified with a Qubit 4 Fluorometer (Life Technologies), pooled, and sequenced on an Illumina NextSeq platform.

### HUVEC, hASC, and vSMC culture

The HUVECs were purchased from PromoCell (pooled donor, C-12203) and hASCs were isolated using a published protocol [76]. HUVECs were then cultured in ECGM2 (PromoCell, C-22011) supplemented with 10% fetal bovine serum (FBS, ScienCell, 0010), with the manufacturer’s supplement Mix solution (PromoCell, C-39215) and 1% penicillin/streptomycin (P/S; Sigma-Aldrich, P4333). The culture plates were coated initially with an Attachment Factor (Cell Applications, 123–500) for 1 h, after which the coating was aspirated without washing. Subsequently, the HUVECs were cultured on these coated plates and maintained at + 37 °C with 5% CO_2_ until they reached 70–80% confluency. The same protocol was used for the iECs.

The hASCs were cultured in DMEM/F12 (Gibco, 21331-020) supplemented with 10% FBS and 1% P/S (ScienCell Research Laboratories, 0503) on noncoated plates. The medium was refreshed every second day and the cells were used for experiments when they reached 70% confluency. HUVECs were used up to passage eight, while hASCs and iECs were used up to passage four. Human umbilical vein smooth muscle cells (vSMCs, ScienCell Research Laboratories, 8020) were cultured using a specific Smooth Muscle Cell Medium (ScienCell Research Laboratories, 1101) prepared by combining 500 ml of Smooth Muscle Cell Medium with 10 ml of FBS (ScienCell Research Laboratories, 0010), 5 ml of smooth muscle cell growth supplement (ScienCell Research Laboratories, 1152) and 5 ml of P/S. Before cell seeding, the culture plates were precoated with poly-l-lysine (ScienCell Research Laboratories, 0403) at a concentration of 0.1 mg/ml diluted in PBS for 1 h. The coating was then washed away with H_2_O two times before seeding the vSMCs.

### Retroviral vector production and transduction of the HUVECs and vSMCs

The genetically modified HUVECs were generated as described previously [10]. Briefly, 293-GPG-VSV-G packaging cells were cultured in a high-glucose DMEM GlutaMAX™ (Gibco, 10569010), 10% FBS, 1% P/S, 0.45 mg/ml G418 (Roche, 04727878001), 1.5 µg/ml tetracycline (Sigma-Aldrich, T3258) and 3 µg/ml puromycin (Sigma-Aldrich, P9620). Packaging cells were transfected with pMXs IG, pMXs IG *TIE2*^WT^ and pMXs IG *TIE2*^L914F^ plasmids, and after 48 h, the medium was collected, filtered and ultra-centrifuged. Ultracentrifuged retroviruses were used to transduce the HUVECs, which were then sorted using FACS (GFP^+^), validated for protein expression and used in the experiments. Tdtomato lentivirus was used to transduce vSMCs.

### Cell lysis and western blot

The confluent 6 cm plates of HUVECs or iECs were washed with ice-cold 1× PBS twice. The cells were then incubated in a mixture of 500 µl ice-cold cell lysis buffer (9.1 mM Na_2_HPO_4_, 1.7 mM NaH_2_PO_4_, pH 7.2, 1% NP-40, 0.25% sodium deoxycholate, 150 mM NaCl, 0.1% SDS, 1 mM EDTA), protease and phosphatase inhibitor cocktails (1:100, Sigma-Aldrich, P8340 and P5726) for 5 min at + 4 °C on a shaker. To homogenize the lysate, the suspension was drawn up and down several times with a syringe and 20G needle and the supernatant was collected from the top after 15 min of centrifugation at 13,000 RPM at + 4 °C, aliquoted and stored at − 80 °C for future use.

To verify the protein level of the transduced HUVECs, a western blot was carried out on the cell lysate samples, the cell lysate proteins being separated in 4% concentrating / 7.5% separating polyacrylamide gel in 1× running buffer (0.2% SDS and 5× running buffer; 0.25 M Tris, 1.92 M glycin, 35 mM SDS). 30 µl of lysate samples were mixed with 10 µl of 4× loading buffer (0.25 M Tris–HCL pH 6.8, 8% SDS, 40% glycerol, 135 mM bromophenol blue) with 1% β-mercaptoethanol (Sigma-Aldrich) and samples were denatured at 95 °C for 5 min. The proteins were separated out by loading and running on SDS-PAGE for 10 min at 100 V and 45 min at 200 V. Blotting was continued by transferring proteins from polyacrylamide gels into a nitrocellulose membrane. For this purpose, one sponge, two Whatman papers, the gel, the nitrocellulose membrane (Millipore, HATF85R), two Whatman papers and another sponge were assembled and soaked in ice-cold 1× transfer buffer (20% ethanol, 25 mM Tris, 920 mM glycine). The transfer was then carried out by running the blot at 1× transfer buffer for 1 h and 30 min, 100 V, at + 4 °C.

To detect the transferred proteins, the nitrocellulose membrane was incubated with antibodies and stained. First, the membrane was blocked in 5% milk in PBST (1× PBS, 0.1% Tween 20) for 1 h, and then washed 2× with PBST. Secondly, the membrane was incubated with primary antibodies (Table S3) diluted 1:1000 in 3% BSA PBST at + 4 °C on a roller rocker overnight. The next morning, after washing the membrane with PBST three times, staining was performed by applying the secondary antibodies (Table S3, 1:10,000) at room temperature (RT) on a roller rocker for 1 h. After 3 × 5 min of washing in PBST, the protein bands were visualized with enhanced chemiluminescence (ECL) reagent (Lumi-Light, Roche, WBKLS0050) and detected using the Azure-600 Biosystem. After each staining, the membrane was rinsed with PBST for 3 × 5 min to reblot with another antibody later. Image J software was used to analyze each band’s intensity and then normalized to β-actin, or to the total form of phosphoprotein of interest.

### hASC/EC co-culture and live cell imaging

To establish a hASC/HUVEC co-culture model suitable for live imaging, cells were seeded on coverslip-bottom 1 cm^2^ gasket chambers (Akita by Finnadvance), following a modified protocol based on previous work [20]. Initially, a thin layer of fibrin gel was applied to the plate. The fibrin gel was prepared by combining 25 µl of 25 mg/ml human fibrinogen (Sigma-Aldrich, 34576) with 25 µl of DMEM-F12 medium. Subsequently, 3 µl of 100 U/ml human thrombin (Sigma-Aldrich, 605190) was added and the mixture was pipetted rapidly and distributed evenly between the gaskets. Next, hASCs were seeded in DMEM-F12 medium at a cell density of 20,000 cells/cm^2^. After 3–5 h, HUVECs were seeded on top of the hASCs at a cell density of 5000 cells/cm^2^ and incubated at + 37 °C with 5% CO_2_ overnight. The following day, a vascular stimulation medium was introduced into the co-culture, with the medium changed every second day until day 7 post-stimulation.

The stimulation medium was DMEM/F12 (Gibco, 11320033), 1% BSA (PANBiotech, P06-138410), 100 mM sodium pyruvate (Gibco, 11360070), l-glutamine (Gibco, 25030081), 5 mg/ml insulin, 5 mg/ml transferrin, 5 mg/ml selenious (ITS; Biosciences, 354351, dissolved in sterile water) and 10 µM tri-iodothyronine (T3; Sigma-Aldrich, T6397, dissolved in 0.1 M NaOH). To finalize the medium, a fresh solution of 25.5 mg/ml l-ascorbic acid (Sigma-Aldrich, A4544, dissolved in 0.1 M NaOH), 1 ng/ml FGF-β (Sigma-Aldrich, SRP4037), 10 µg/ml VEGF (Sigma-Aldrich, SRP3182), 5 mg/ml hydrocortisone solution (Sigma-Aldrich, H0888, dissolved in absolute ethanol) and 0.2% heparin (Stemcell Technologies, 07980) was added. For live cell imaging, glass bottom plates (Ibidi, 8158) with lids were used, and the medium was refreshed every day.

Multi-position live cell imaging was performed using a fully motorized Zeiss LSM 780 laser scanning confocal microscope. The objective used was a Plan-Apochromat 10×/0.45 DIC (air). Imaging was performed at 20 min intervals in a top stage incubator for live cell time-lapse imaging with temperature, humidity, CO_2_ and O_2_ control. Samples were imaged at 488 nm, with a spectral fluorescence GaAsP array detector.

### vSMC/EC 2D double layer co-culture

The plates were initially coated with the Attachment Factor, followed by seeding of HUVECs at a density of 5000 cells/cm^2^. vSMCs were then seeded the next day on top of the cultured ECs at a density of 1250 cells/cm^2^. The ratio of ECs to vSMCs (4 to 1) was used to facilitate the confluent EC monolayer for stable cell–cell contacts and paracrine communication, as well as sufficiently high number of vSMCs for their subsequent analysis. Cell tracking was performed on single cells of the ECs and vSMCs separately using the CellTracker program developed by Piccini et al. in MatLab 2012 and Image Processing Toolbox 8.1 [77].

### Immunofluorescent staining

Specimens were fixed using 4% PFA for 15 min at RT then underwent PBST for 3 × 5 min and were subsequently subjected to simultaneous blocking and staining with primary antibodies (Table S4) in 3% BSA-PBST overnight at + 4 °C without shaking to prevent fibrin gel damage. The next day the blocking-staining solution was aspirated, and the samples were washed for 3 × 5 min each. They were then treated with secondary antibodies (Table S4) in 3% BSA-PBST for 2 h at RT, followed by another 3 × 5 min post-treatment wash with PBST before storage at + 4 °C covered with PBST until imaging.

### hASC/EC microscopy

Imaging of the fixed samples was performed using either a Leica SP8 FALCON laser scanning confocal or a Leica STELLARIS 8 DIVE multiphoton confocal microscope. The objectives used in the FALCON were HC PL APO 10×0.40 CS2 (air), HC PL APO 20×/0.75 CS2 (air), HC PL APO 40×/1.10 W motCORR CS2 (water) and HC PL APO 63×/1.40 OIL CS2 DIC (oil), and the samples were imaged with a 405 nm diode and supercontinuum 470–670 nm laser with spectral fluorescence detectors (3 HyD, 1 PMT). The images were acquired with a fully motorized inverted microscope and a stage for multi-position and tile-scan imaging using high-resolution galvanometric scanning and operated with LAS X software. The objectives used with the STELLARIS 8 DIVE were HC PL APO 10×/0.40 CS (air), HC PL APO 20×/0.75 CS2 (air), HC PL APO 40×/1.10 W CORR CS2 (water, for both confocal and multiphoton) and HC PL APO 63×/1.40 OIL CS2 (oil), and the samples were imaged with 405 nm, 488 nm, 561 nm and 638 nm lasers with spectral fluorescence detectors for confocal use (3 HyD S). The images were acquired with a fully motorized inverted microscope and a stage for multi-position and tile-scan imaging, operated by LAS X software.

### Live cell imaging of vSMC/HUVEC co-cultures

The plates for live cell imaging of 2D double layer cultures in the vSMC/HUVECs were initially coated with the Attachment Factor, followed by seeding of the HUVECs at a density of 5000 cells/cm^2^. The vSMCs were then seeded the next day on top of the cultured HUVEC at a density of 1250 cells/cm^2^. Imaging commenced on the following day and was performed using the Olympus CellSens time-lapse imaging system using a CPlanFLN PhC 10×/0.30 objective. Imaging was performed in a top stage incubator with temperature, humidity and CO_2_ controlled to suit 6-well plates with a time interval of 20 min. The samples were imaged with GFP and TRICT filters, an Olympus XM10 CCD camera operated with Olympus CellSens software.

### Image analysis

Fiji (ImageJ) was used for processing and quantification of the fixed images, the confocal images being opened as separate channels and z-stacked using the maximum intensity projection method. The images were then converted to an 8-bit grayscale format, followed by automatic thresholding without manual intervention. To quantify the selected areas, the Analyze Particles tool was used to measure and sum the segmented regions. Tubule diameters and ECM patterns were measured using the Straight-Line tool at ten randomly selected locations per image. All the analyses were conducted under consistent thresholding and measurement parameters to ensure reproducibility.

### FACS sorting

Cells were resuspended in cold PBS supplemented with 1% FBS and sorted based on either GFP or tdTomato expression. The cells were then centrifuged and either pelleted for culture and expansion or immediately snap-frozen in liquid nitrogen for RNA extraction. A BD FACS Discover™ S8 Cell Sorter was used for the hASC/ HUVEC experiments and BD FACS Aria III for the vSMC/HUVEC experiment.

### RNA extraction

RNA was extracted using the RNeasy Mini Kit (QIAGEN, 74104). Cells were lysed, mixed with ethanol, and passed through a spin column to bind RNA. The column was washed with buffers, dried, and RNA was eluted with RNase-free water. DNase treatment with RNase-Free DNase (QIAGEN, 79254) removed genomic DNA. The extracted RNA was sent to Novogene for mRNA sequencing.

### RNA sequencing analysis

FASTQ files were obtained from NovoGene, and RNAseq was performed with unstranded, paired-end sequencing. The raw reads then underwent quality control, trimming and quantification using Salmon in its pseudo-alignment mode. Gene-level counts were aggregated, and DESeq2 [78] was used for differential expression analysis, normalization of the data and identification. PCA was performed on variance-stabilized data to evaluate clustering of the samples. Significantly enriched KEGG and GO pathways were identified by functional enrichment analysis using ClusterProfiler [79]. Gene Ontology (GO) and KEGG pathway enrichment analyses were performed on differentially expressed genes (DEGs) to explore their functional significance. GO analysis covered the biological process, cellular component and molecular function categories using the ‘go_enrich’ function of Bioconductor in R with significance set at adj.*P* < 0.05, while the KEGG pathway analysis was carried out using the ‘enrichKEGG’ function of Bioconductor in R, highlighting significantly enriched metabolic and signaling pathways among the DEGs. All programming and plotting took place in Rstudio (R 4.3.3).

### Statistical analysis

All the statistical analyses were carried out with GraphPad Prism 9 software. The unpaired two-tailed Student’s *t*-test was used to compare two groups, one-way ANOVA for multiple group comparisons, and two-way ANOVA was used for multiple comparisons. Tukey’s post hoc test was used for comparisons. Statistical significance is denoted in the figures by: *P < 0.05; **P < 0.01; ***P < 0.001; and ****P < 0.0001.

## Supplementary Information

Below is the link to the electronic supplementary material.

Supplementary Figures and Tables

Supplementary Movie 1. Co-culture of hASC / HUVEC_GFP

Supplementary Movie 2. Co-culture of hASC / HUVEC_GFP TIE2_L914F

Supplementary Pathway enrichment analysis

Supplementary RNAseq raw data

Supplementary DEGs

## Data Availability

The transcriptomic data is presented in the major figures of this article and its supplementary information files.
