# Supplementary material for: A human endothelial and adipose stem cell-based co-culture model for venous malformations
Source: Angiogenesis. 2026 May 3;29(3):30. doi: 10.1007/s10456-026-10045-9 (PMC13136223; doi:10.1007/s10456-026-10045-9)
Supplement: Supplementary file 1 — Supplementary Figures and Tables [file 10456_2026_10045_MOESM1_ESM.docx]

**Human endothelial and adipose stem cell -based co-culture model for venous malformations**

Mohammadhassan Ansarizadeh, Bojana Lazovic, Zahra Sarmadian, Abhishek Singh,

Ryan Hicks, Lauri Eklund

**SUPPLEMENTARY MATERIAL**


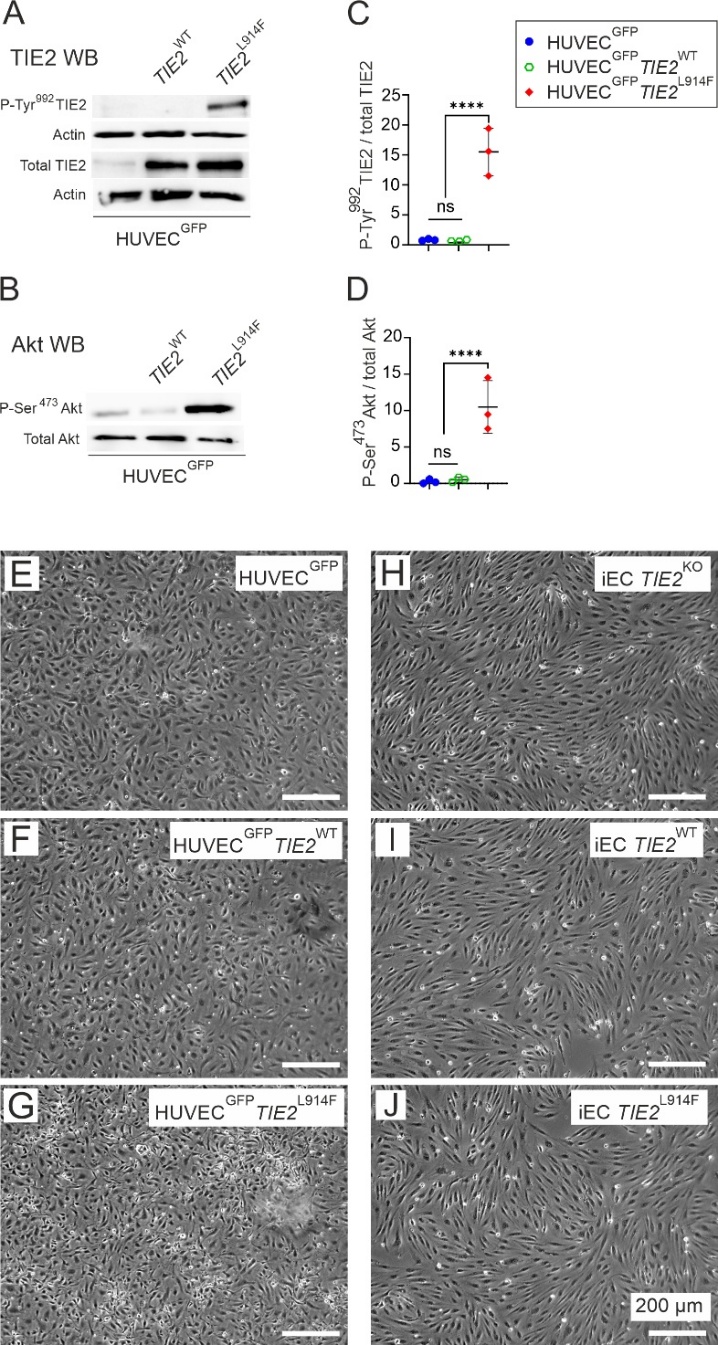


**Fig. S1. Western blot analysis and phase-contrast microscopy of control and genetically modified HUVECs and iECs**. HUVECs were transduced with bicistronic TIE2-IRES-GFP or only GFP-expressing retroviruses as the control. (**A, B**) TIE2 and Akt protein expression and activation (phosphorylation states) were investigated using western blotting (WB) from cell lysates and (**C-D**) signal intensities were quantified. Retroviral expression of VM-causative *TIE2*^L914F^ mutation results in increased TIE2 tyrosine phosphorylation (P-Tyr) indicating a gain-of-function effect and downstream activation of Akt. (**E-J**) EC monolayer morphology imaged using phase contrast microscopy. (**G**) Overexpression of *TIE2*^L914F^ in HUVECs results in an elongated and partially overlapping cellular phenotype instead of EC cobblestone morphology in controls. (**H-J**) In iECs monoculture, TIE2 deficiency or *TIE2*^L914F^ substitution does not change cellular morphology. ****P<0.0001; ns, statistically not significant in one way ANOVA followed by Tukey's post hoc test. n, three independent experiments. Means ± SD. Scale bar, 200 µm.


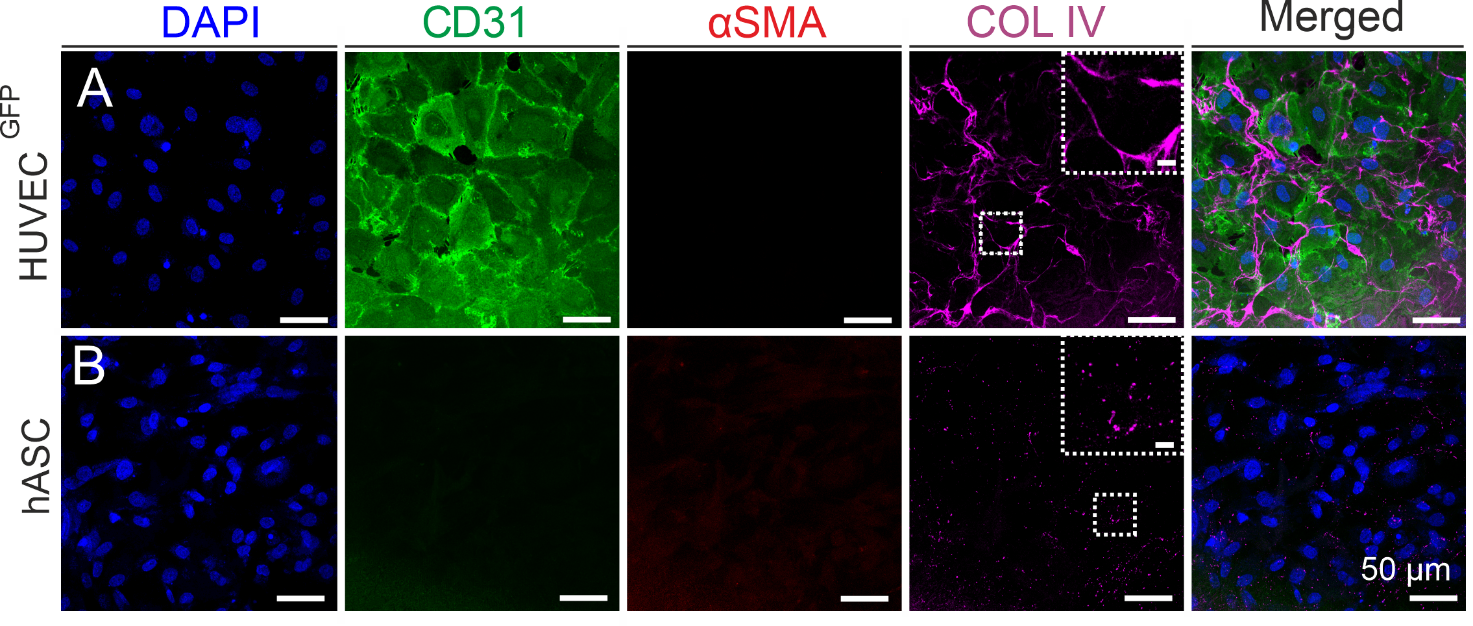
**Fig. S2. Monocultures of HUVEC^GFP^ and hASC**. Cells were grown separately on the fibrin gel in vascular stimulation media for 7 days, fixed, and stained as indicated. Note that in the monoculture capillary-like EC structures and αSMA expressing cells are absent and that COL IV deposition is qualitatively (fibrillar structure in monoculture) and quantitatively different (less in monoculture) from the co-culture model. Scale bar, 50 µm.


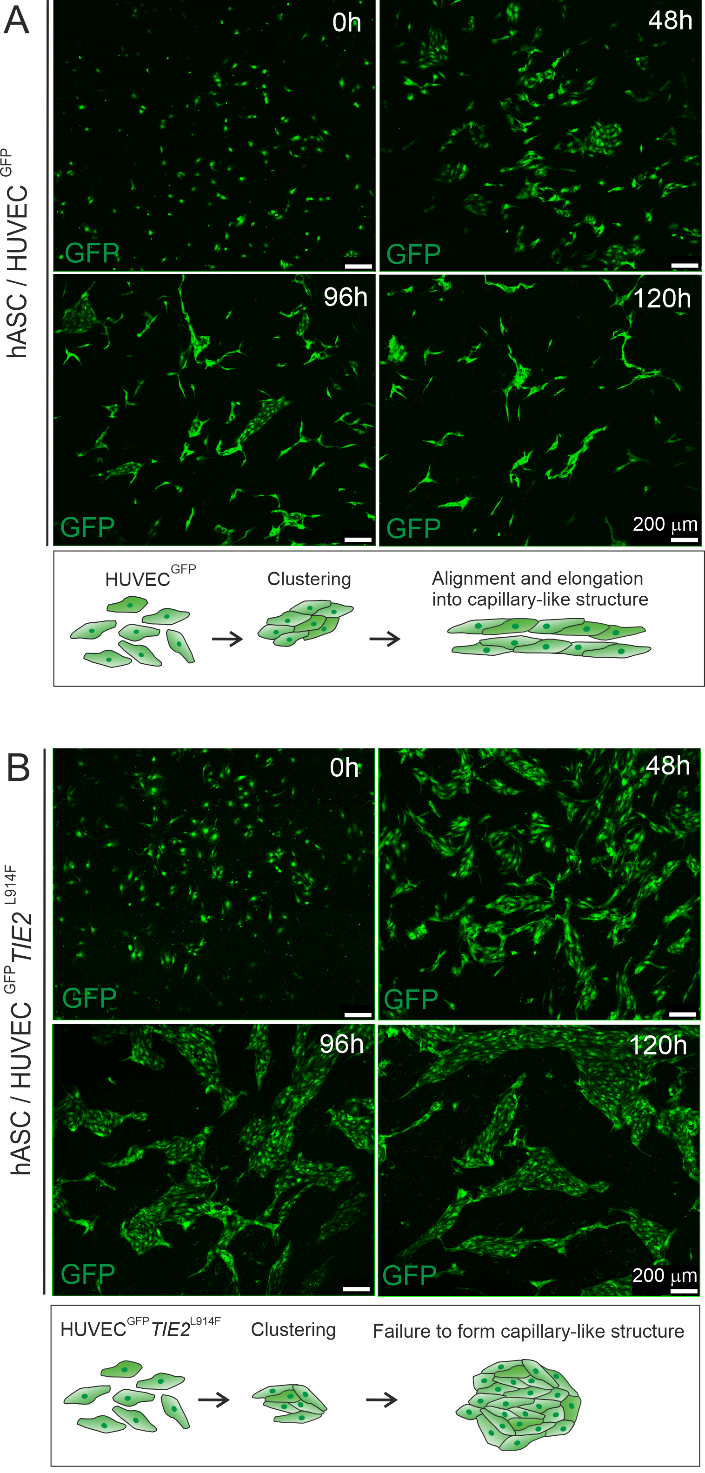


**Fig. S3. Selected timepoints from time-lapse imaging of hASCs/HUVECs co-culture**. (**A**) EC structures formed by control HUVEC^GFP^. (**B**) HUVEC^GFP^ *TIE2*^L914F^ failed to form capillary-like structures.


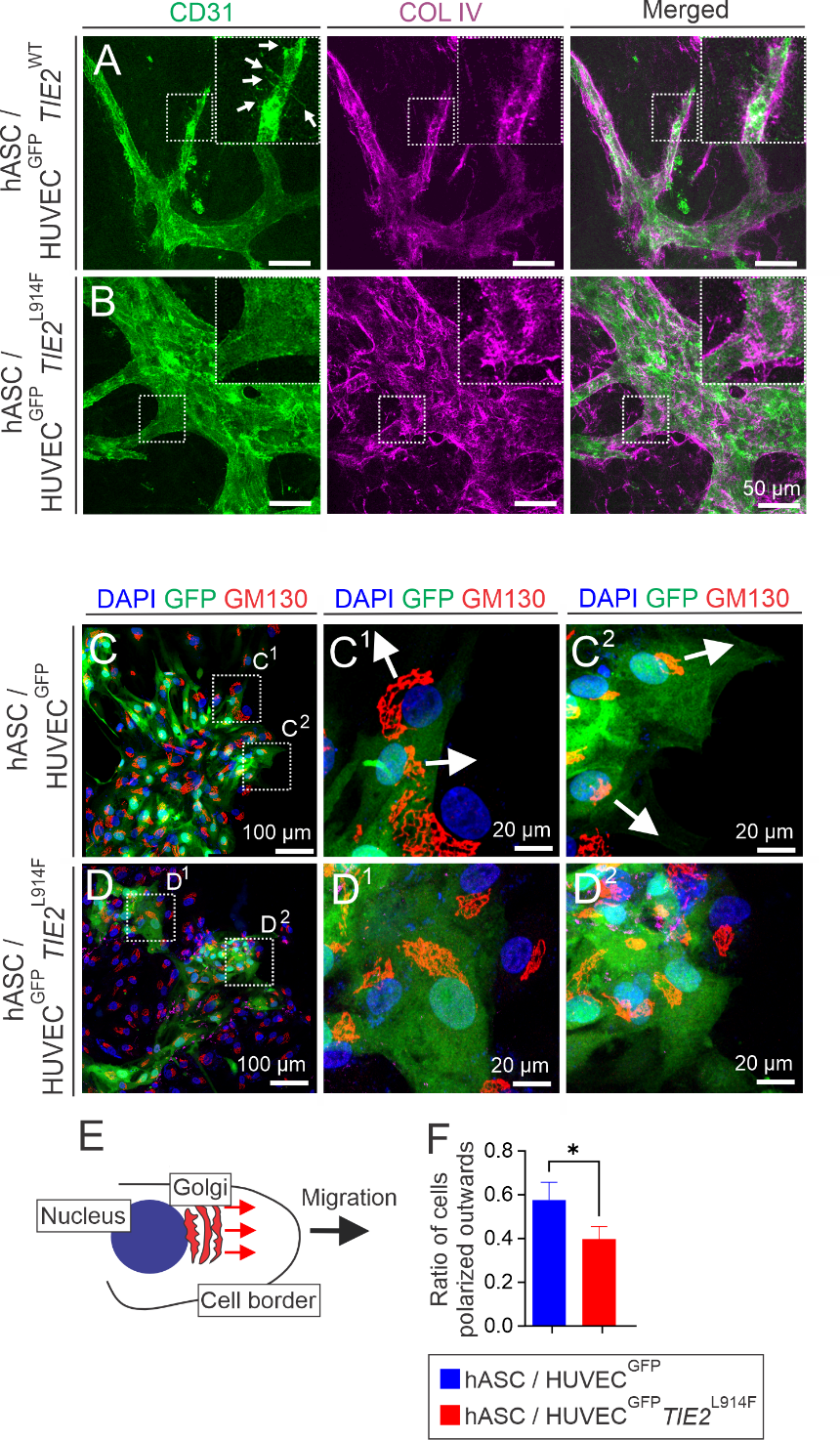


**Fig. S4. Deficit characteristics of sprouting angiogenesis in hASC/HUVEC^GFP^ *TIE2*^L914F^**. (**A**) HUVEC^GPF^ *TIE2*^WT^ (CD31, green) shows numerous filopodia (arrows). (**B**) *TIE2*^L914F^ EC structures lack sprouting pattern and show few filopodia. (**C-F**) Front-rear cell polarity of HUVECs at the border of cell cluster. (**C-D**) Immunofluorescence staining. (**E**) Cartoon illustrates how the polarity of motile cells is predicted; the Golgi is towards the direction of migration and positioned at the front edge of the nucleus. (**F**) Quantification of HUVECs migrating outward; in the control HUVECs the Golgi apparatus (GM130, red in C and D) shows the higher tendency to polarize outwards (white arrows) of cell clusters. *P<0.05 in t-test. n, three independent experiments. Means ± SD.


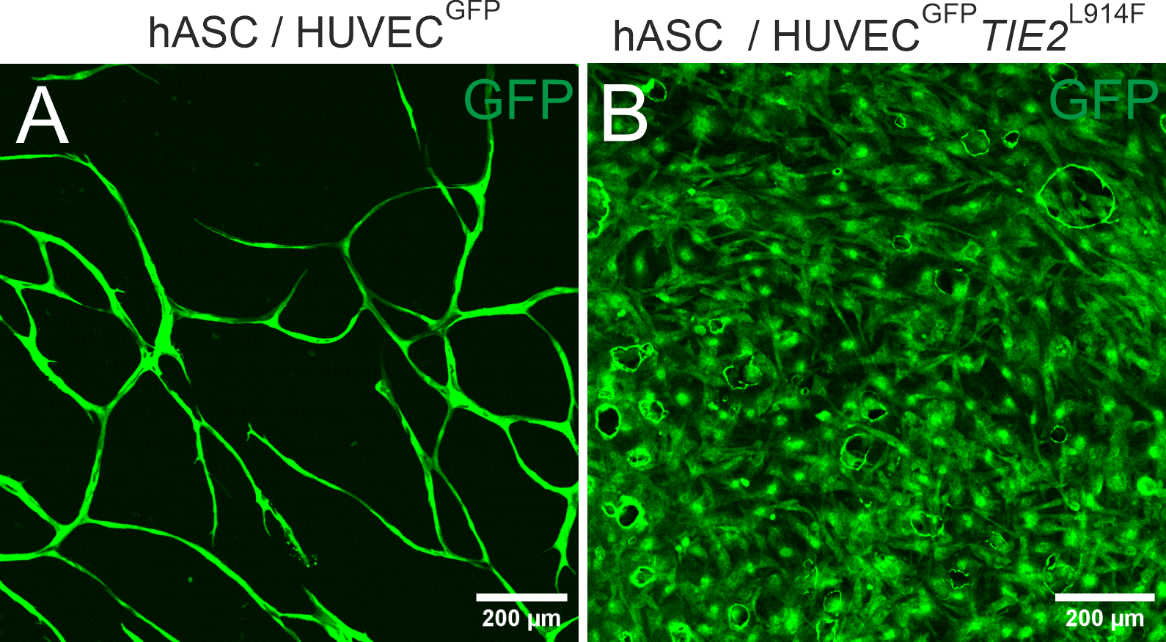


**Fig. S5**. Two-week co-culture of HUVEC^GFP^ and HUVEC^GFP^ *TIE2*^L914F^. (**A**) HUVEC^GFP^ formed well connected network of capillary-like structures and branching while (**B**) HUVEC^GFP^ *TIE2*^L914F^ failed to form similar EC arrangement after extended culture time.


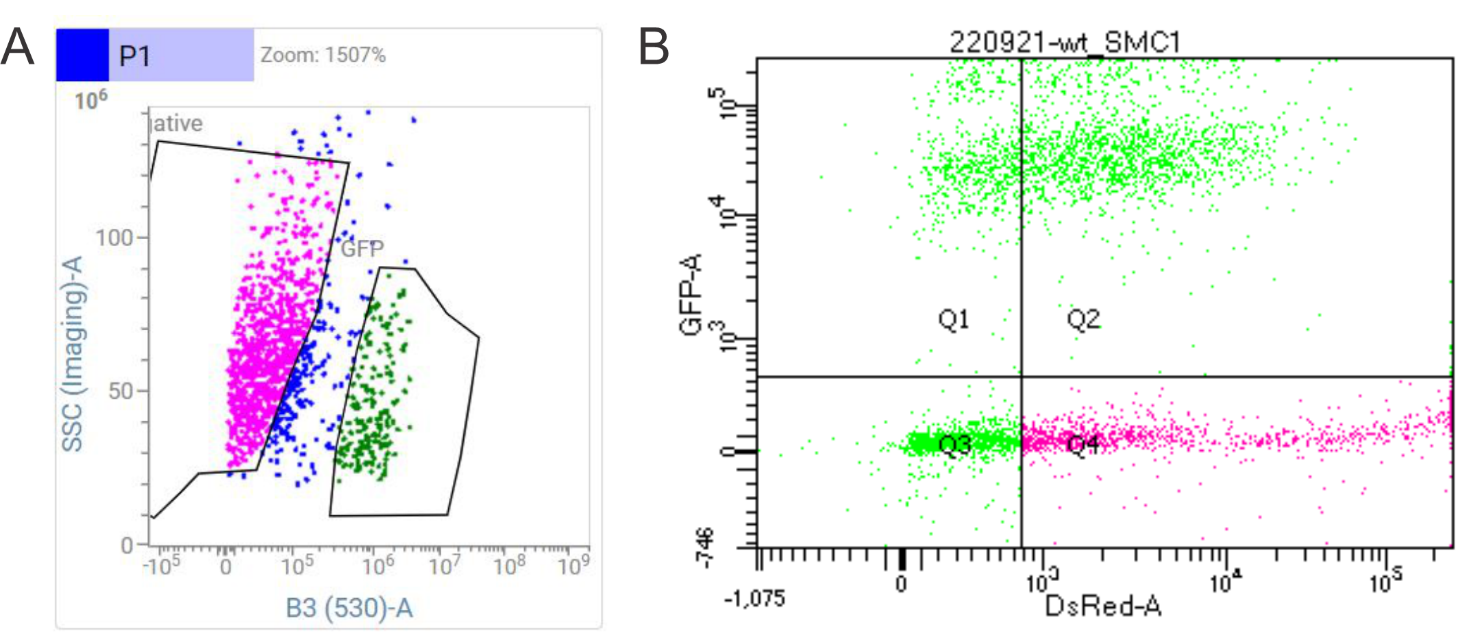
**Fig. S6. Sorting of hASCs, vSMCs and HUVECs using fluorescence-activated cell sorting (FACS).** (**A**) hASCs (GFP^-^) were sorted from GFP^+^ HUVECs using BD FACS Discover™ S8 Cell Sorter, hASCs are presented in pink, HUVECs in green, blue indicates low GFP^+^ cells not included in the analysis. (**B**) tdTomato^+^ vSMCs^tdT^ sorted from GFP^+^ HUVECs using BD FACS Aria III; Q4 represent tdT^+^ vSMCs free from GFP^+^ cells.


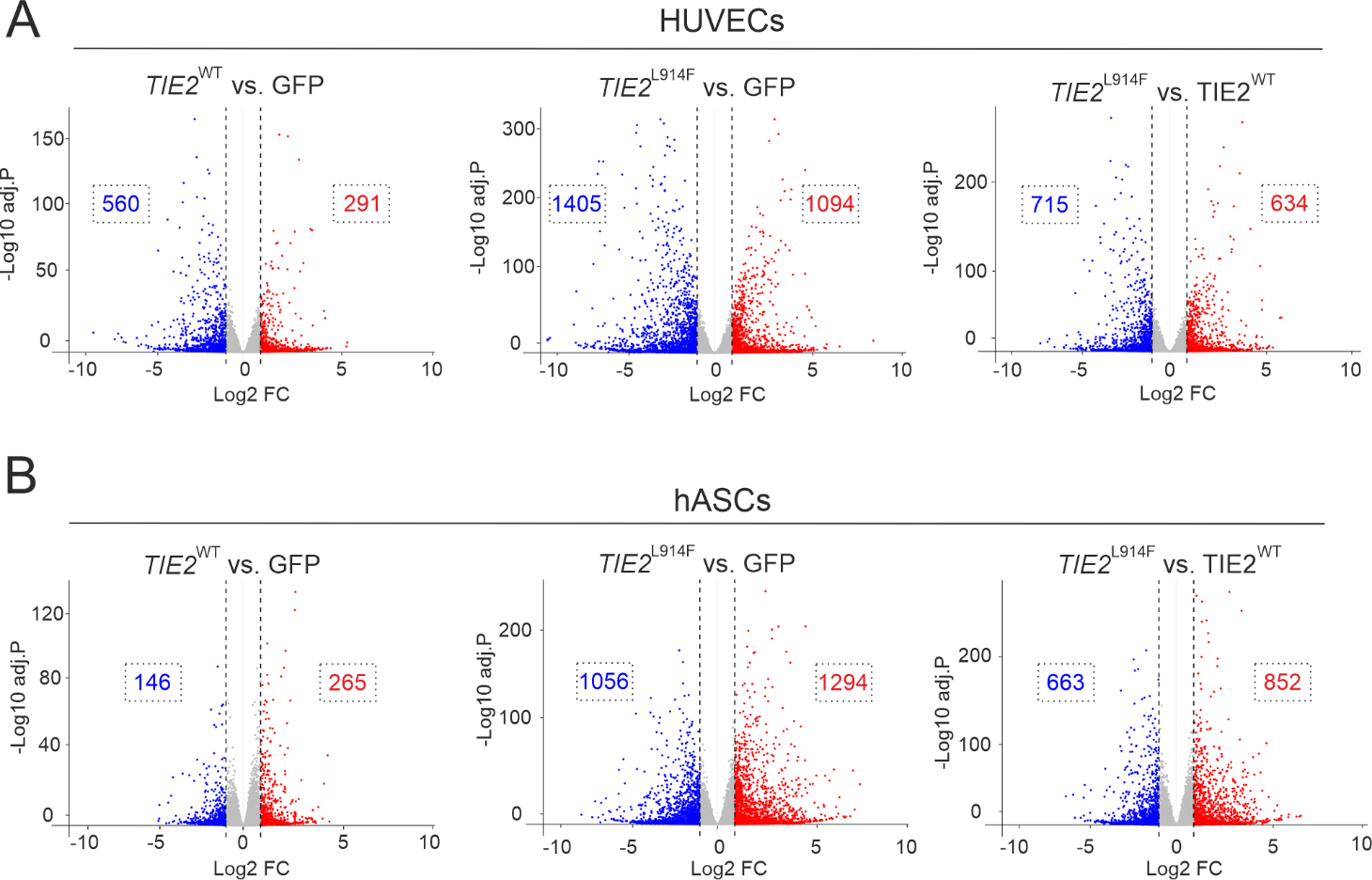


**Fig. S7. Transcriptomic analysis of HUVECs and hASCs in co-culture model**. Volcano plots display differentially expressed genes (DEGs) in (**A**) HUVECs and (**B**) hASCs in the comparisons indicated. The genes in blue/red are down/upregulated and the number of DEGs in each case are written in dashed box. The highest number of DEGs was observed in the hASC/HUVEC^GFP^ vs. hASC/HUVEC^GFP^ *TIE2*^L914F^ comparison.


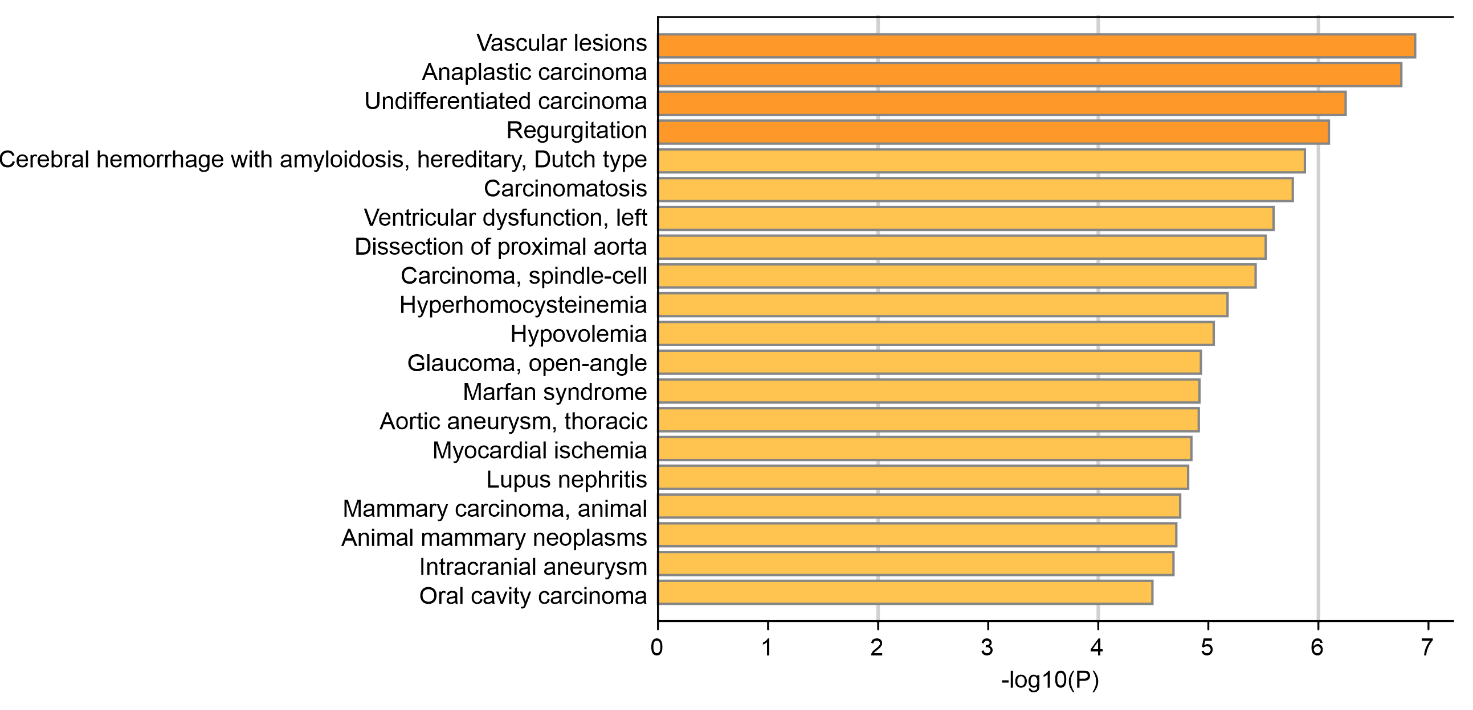
**Fig. S8.** Pathway enrichment (DisGeNet) analysis on the mutual upregulated genes among HUVECs isolated from hASC/HUVEC *TIE2*^L914F^ co-culture, iEC *TIE2*^L914F^ and patient-derived TIE2-VM data from [1].


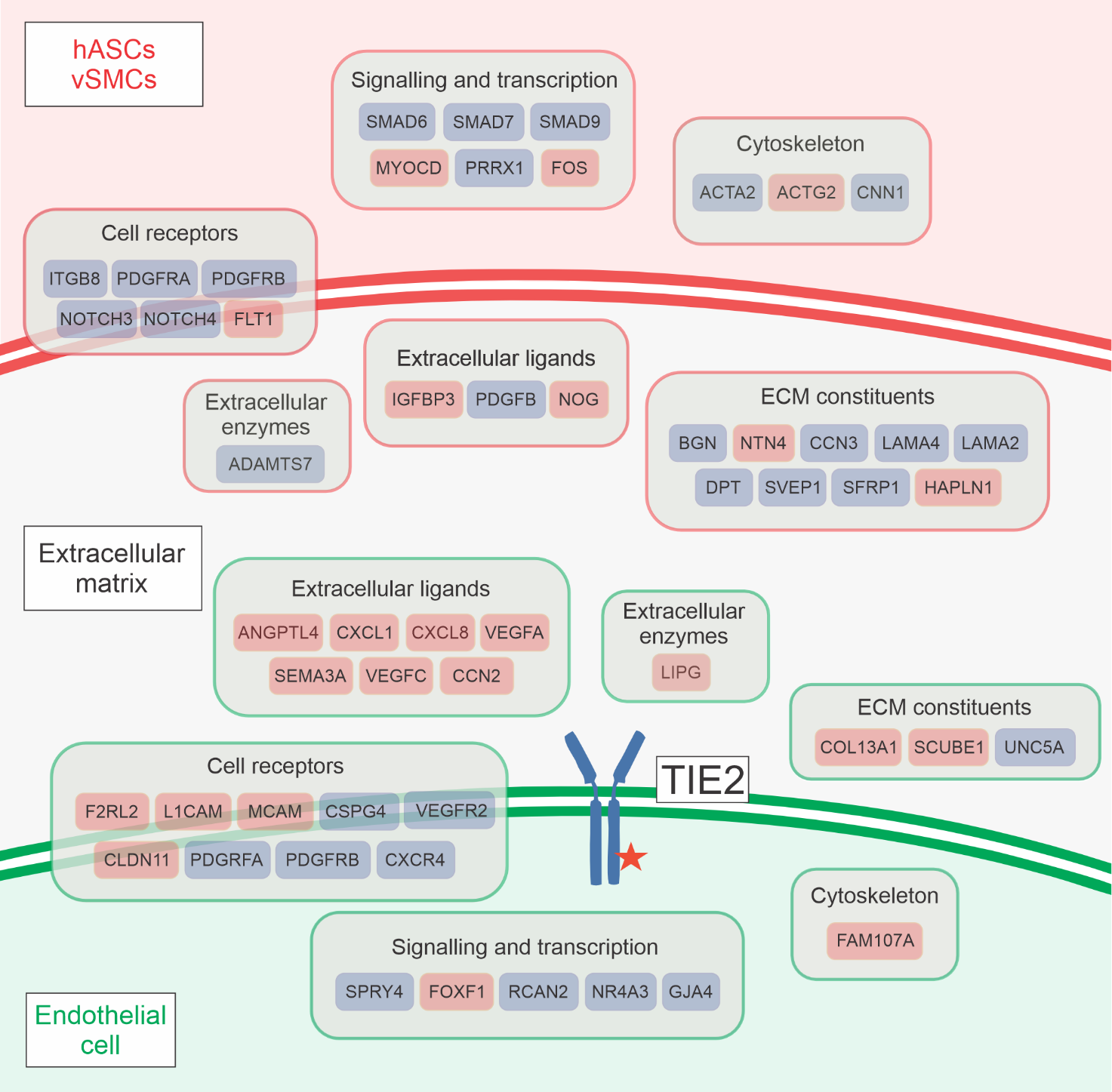
**Fig. S9. Visualization of differentially expressed genes identified in ECs, hASCs and vSMCs involved in vascular cell signaling and morphogenesis.** The top enriched GO terms were first ranked by adjusted P-value (<0.05), the most potential DEGs were narrowed down by fold change (Log2FC >1 or Log2FC <-1) and by known biological relevance in each cell type. DEGs identified in HUVECs are framed in green, DEGs in hASCs and vSMCs in red. The DEGs upregulated are on red and DEGs downregulated are on blue background.

**Supplementary Movie 1. Co-culture of hASC / HUVEC^GFP^**. Time-lapse Z-stacked images were captured at 20 min intervals and converted to maximum intensity projections. Imaging started immediately after EC seeding and continued for five days. Examples of formed lumen structure (arrow) and sprouting behavior (framed) are annotated in the movie (at 89.1 h time point).

**Supplementary Movie 2. Co-culture of hASC / HUVEC^GFP^*TIE2*^L914F^**. Time-lapse Z-stacked images were captured at 20 min intervals and converted to maximum intensity projections. Imaging started immediately after EC seeding and continued for five days.

**Table S1. Sequences of sgRNA guides used for gene editing of *TIE2*^KO^ and *HBEGF***

| *TIE2*^KO^ |  |
| --- | --- |
| sgRNA left: | |
| ATTGATCAAGATCAAGTCCA | |
| Reverse complement: TGGACTTGATCTTGATCAAT |  |
| sgRNA right: |  |
| ATGAGCCCATCACCATAGGA |  |
| *HBEGF* selection | |
| sgRNA: | |
| CACCTCTCTCCATGGTAACC | |

**Table S2. PCR primers for Amplicon-sequencing analysis**

|  | Indexing part | Annealing primer |
| --- | --- | --- |
| *TIE2*^KO^_NGS_F | TCGTCGGCAGCGTCAGATGTGTATAAGAGACAG | ACAAGGCTTTGTGAGCACCAG |
| *TIE2*^KO^_NGS_R | GTCTCGTGGGCTCGGAGATGTGTATAAGAGACAG | AGCCCATTCTCTGGTCACATCTT |
| Tm | 72°C |  |
| Product size | 280 bp |  |

**Table S3. Primary and secondary antibodies in immuno-precipitation and western blot**

| **Primary antibody** (IgG type, manufacturer, catalog number) | **Secondary antibody** (IgG type, manufacturer, catalog number) |
| --- | --- |
| Total-TIE2 (Clone Ab33, mouse monoclonal, Merck Millipore, 05-584) | HRP-conjugated goat anti-mouse IgG (Affinipure, 115-035-003) |
| Phospho-TIE2 (Y992, rabbit polyclonal, Cell Signaling Technology, 4221S) | HRP-conjugated goat anti-rabbit IgG (Affinipure, 111-035-003) |
| Total-Akt (Rabbit polyclonal, Cell Signaling Technology, 9272S) | HRP-conjugated goat anti-rabbit IgG (Affinipure, 111-035-003) |
| Phospho-Akt (Ser 473, rabbit polyclonal, Cell Signaling Technology, 4060B) | HRP-conjugated goat anti-rabbit IgG (Affinipure, 111-035-003) |
| Total-TIE2 (Clone Ab33, mouse monoclonal, Merck Millipore, 05-584) | Rabbit anti-mouse IgG (Jackson ImmunoResearch, 315-005-045) |
| β-Actin (Sigma, A5441) | HRP-conjugated goat anti-rabbit IgG (Affinipure, 111-035-003) |

**Table S4. Primary and secondary antibodies in immunofluorescent staining**

| **Primary antibody or fluorescent stain** (IgG type, manufacturer, catalog number) | **Dilution** | **Secondary antibody** (IgG type, manufacturer, catalog number) | **Dilution** |
| --- | --- | --- | --- |
| Human CD31 (mouse polyclonal, Dako, M0823) | 1:500 | Alexa Flour 488-conjugated donkey anti-mouse IgG (Jackson ImmunoResearch, 715-545-151) | 1:1000 |
| Human COL IV (rabbit polyclonal, Merk Millipore, AB756P) | 1:1000 | Alexa Flour 647-conjugated donkey anti-rabbit IgG (Jackson ImmunoResearch, 711-605-152) | 1:1000 |
| αSMA-Cy3-conjugated (mouse monoclonal, Sigma-Aldrich, C6198) | 1:1000 | - |  |
| DAPI (Sigma-Aldrich, D9542) | 1:1000 | - |  |
| GM130 (mouse monoclonal, BD Pharmingen, 610822) | 1:1000 | Alexa Flour 647-conjugated donkey anti-rabbit IgG (Jackson ImmunoResearch, 711-605-152) | 1:1000 |

**SUPPLEMENTARY REFERENCES**

1. Hirose K, Hori Y, Ozeki M, et al (2024) Comprehensive phenotypic and genomic characterization of venous malformations. Hum Pathol 145:. https://doi.org/10.1016/j.humpath.2024.02.004
