# Supplementary material for: A human endothelial and adipose stem cell-based co-culture model for venous malformations
Source: Angiogenesis. 2026 May 3;29(3):30. doi: 10.1007/s10456-026-10045-9 (PMC13136223; doi:10.1007/s10456-026-10045-9)
Supplement: Supplementary file 4 — Supplementary Pathway enrichment analysis [file 10456_2026_10045_MOESM4_ESM.zip › PathwayEnrichment analysis/hASC/Supplementary_GO_hASC_WT_vs_L914F_Biological Process.pdf]

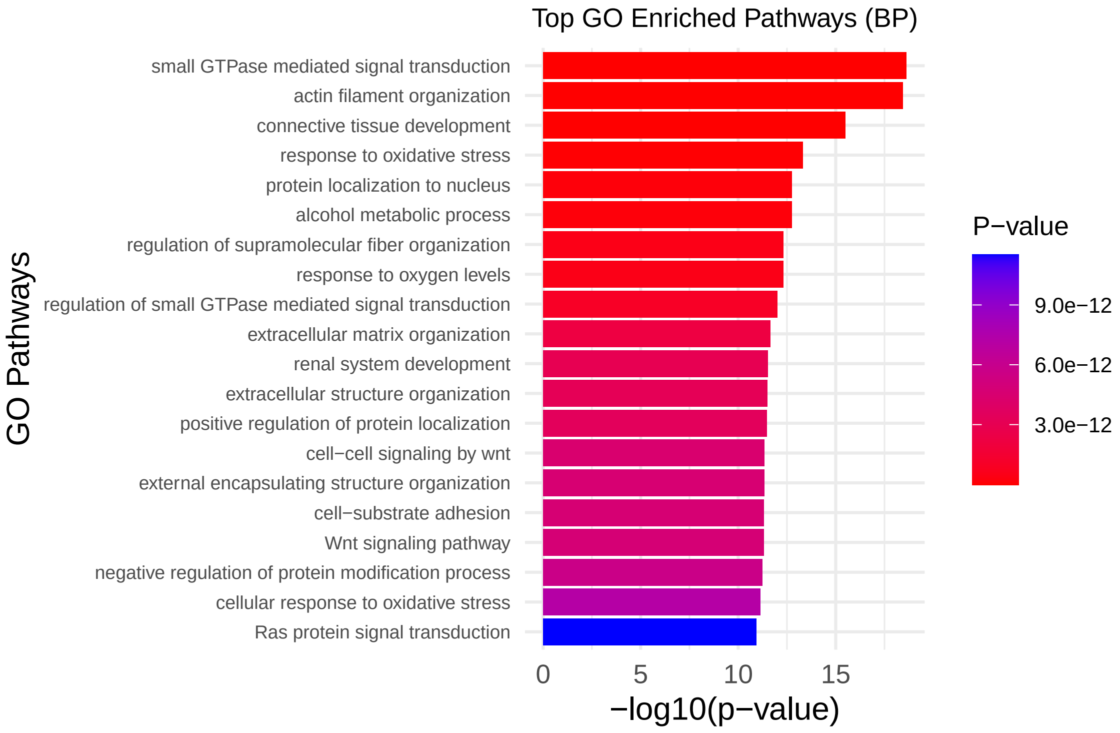

Top GO Enriched Pathways (BP)
GO Pathways
small GTPase mediated signal transduction
actin filament organization
connective tissue development
response to oxidative stress
protein localization to nucleus
alcohol metabolic process
regulation of supramolecular fiber organization
response to oxygen levels
regulation of small GTPase mediated signal transduction
extracellular matrix organization
renal system development
extracellular structure organization
positive regulation of protein localization
cell−cell signaling by wnt
external encapsulating structure organization
cell−substrate adhesion
Wnt signaling pathway
negative regulation of protein modification process
cellular response to oxidative stress
Ras protein signal transduction
0
5
10
15
−log10(p−value)
P−value
9.0e−12
6.0e−12
3.0e−12
