# Supplementary material for: A human endothelial and adipose stem cell-based co-culture model for venous malformations
Source: Angiogenesis. 2026 May 3;29(3):30. doi: 10.1007/s10456-026-10045-9 (PMC13136223; doi:10.1007/s10456-026-10045-9)
Supplement: Supplementary file 4 — Supplementary Pathway enrichment analysis [file 10456_2026_10045_MOESM4_ESM.zip › PathwayEnrichment analysis/HUVECs/Supplementary_GO_HUVECs_GFP_vs_WT-Biological Process.pdf]

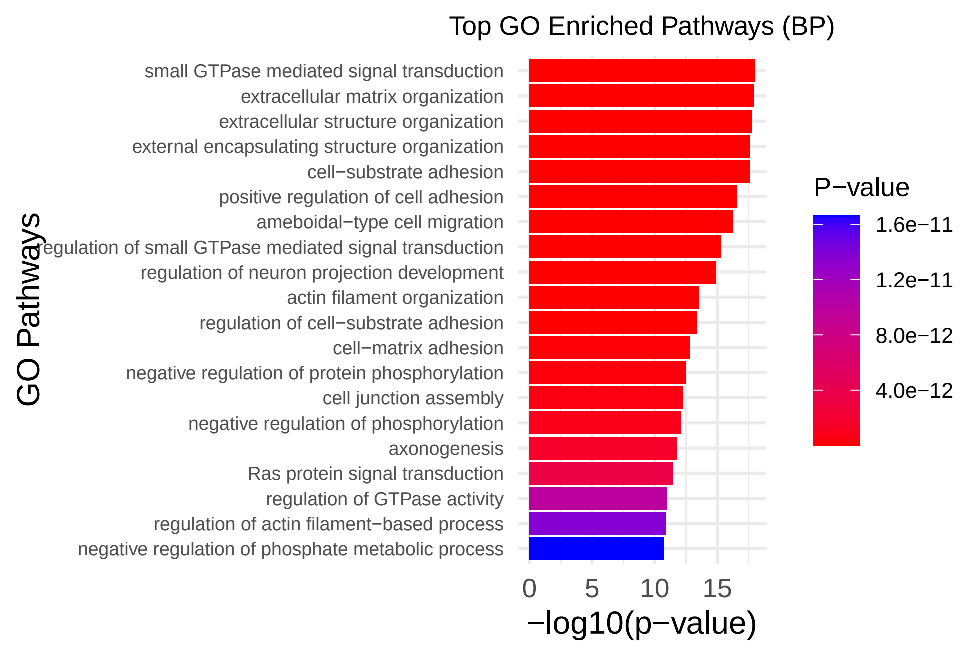
Top GO Enriched Pathways (BP)
GO Pathways
small GTPase mediated signal transduction
extracellular matrix organization
extracellular structure organization
external encapsulating structure organization
cell−substrate adhesion
positive regulation of cell adhesion
ameboidal−type cell migration
regulation of small GTPase mediated signal transduction
regulation of neuron projection development
actin filament organization
regulation of cell−substrate adhesion
cell−matrix adhesion
negative regulation of protein phosphorylation
cell junction assembly
negative regulation of phosphorylation
axonogenesis
Ras protein signal transduction
regulation of GTPase activity
regulation of actin filament−based process
negative regulation of phosphate metabolic process
0
5
10
15
−log10(p−value)
P−value
1.6e−11
1.2e−11
8.0e−12
4.0e−12
