# Supplementary material for: A human endothelial and adipose stem cell-based co-culture model for venous malformations
Source: Angiogenesis. 2026 May 3;29(3):30. doi: 10.1007/s10456-026-10045-9 (PMC13136223; doi:10.1007/s10456-026-10045-9)
Supplement: Supplementary file 4 — Supplementary Pathway enrichment analysis [file 10456_2026_10045_MOESM4_ESM.zip › PathwayEnrichment analysis/HUVECs/Supplementary_GO_HUVECs_GFP_vs_WT-Cellular Component.pdf]

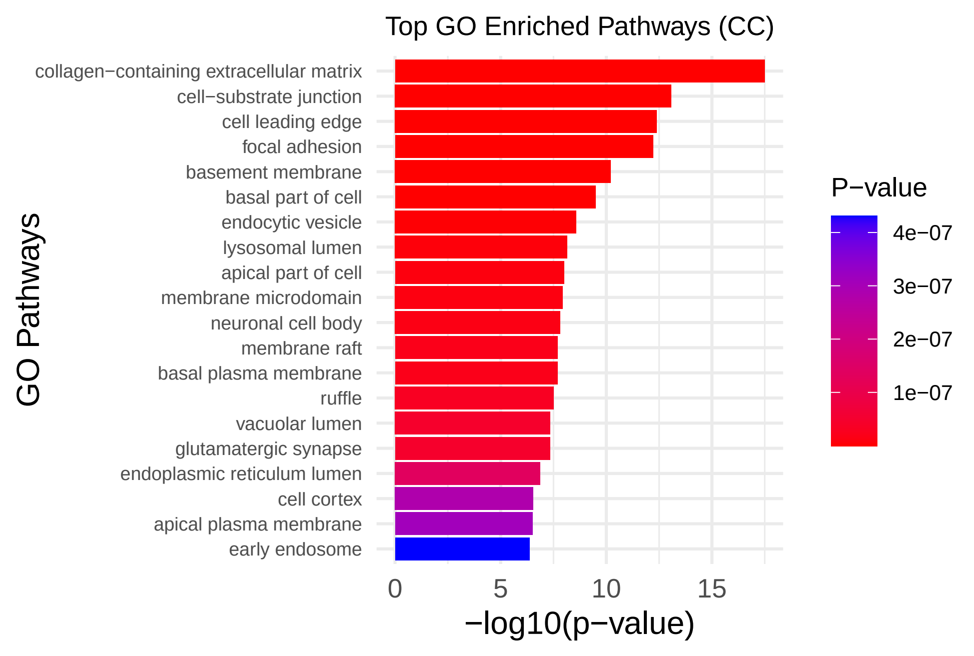

Top GO Enriched Pathways (CC)
GO Pathways
collagen−containing extracellular matrix
cell−substrate junction
cell leading edge
focal adhesion
basement membrane
basal part of cell
endocytic vesicle
lysosomal lumen
apical part of cell
membrane microdomain
neuronal cell body
membrane raft
basal plasma membrane
ruffle
vacuolar lumen
glutamatergic synapse
endoplasmic reticulum lumen
cell cortex
apical plasma membrane
early endosome
0
5
10
15
−log10(p−value)
P−value
4e−07
3e−07
2e−07
1e−07
