# Supplementary material for: A human endothelial and adipose stem cell-based co-culture model for venous malformations
Source: Angiogenesis. 2026 May 3;29(3):30. doi: 10.1007/s10456-026-10045-9 (PMC13136223; doi:10.1007/s10456-026-10045-9)
Supplement: Supplementary file 4 — Supplementary Pathway enrichment analysis [file 10456_2026_10045_MOESM4_ESM.zip › PathwayEnrichment analysis/HUVECs/Supplementary_GO_HUVECs_WT_vs_L914F-Molecular Function.pdf]

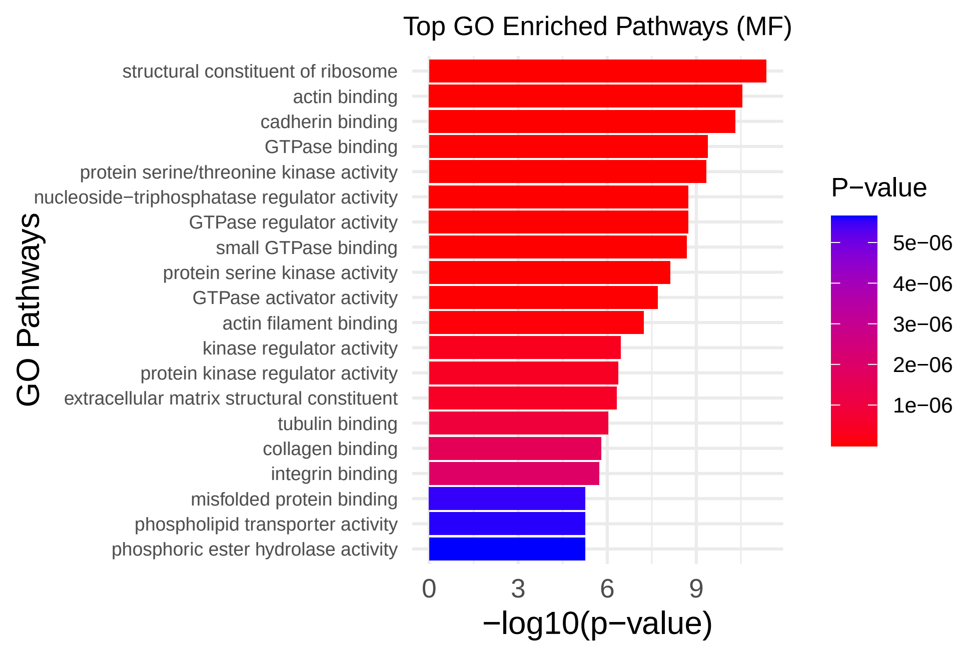

Top GO Enriched Pathways (MF)
GO Pathways
structural constituent of ribosome
actin binding
cadherin binding
GTPase binding
protein serine/threonine kinase activity
nucleoside−triphosphatase regulator activity
GTPase regulator activity
small GTPase binding
protein serine kinase activity
GTPase activator activity
actin filament binding
kinase regulator activity
protein kinase regulator activity
extracellular matrix structural constituent
tubulin binding
collagen binding
integrin binding
misfolded protein binding
phospholipid transporter activity
phosphoric ester hydrolase activity
0
3
6
9
−log10(p−value)
P−value
5e−06
4e−06
3e−06
2e−06
1e−06
