# Supplementary figures and images for: A human endothelial and adipose stem cell-based co-culture model for venous malformations
Source: Angiogenesis. 2026 May 3;29(3):30. doi: 10.1007/s10456-026-10045-9 (PMC13136223; doi:10.1007/s10456-026-10045-9)

# Top GO Enriched Pathways (BP)

GO Pathways

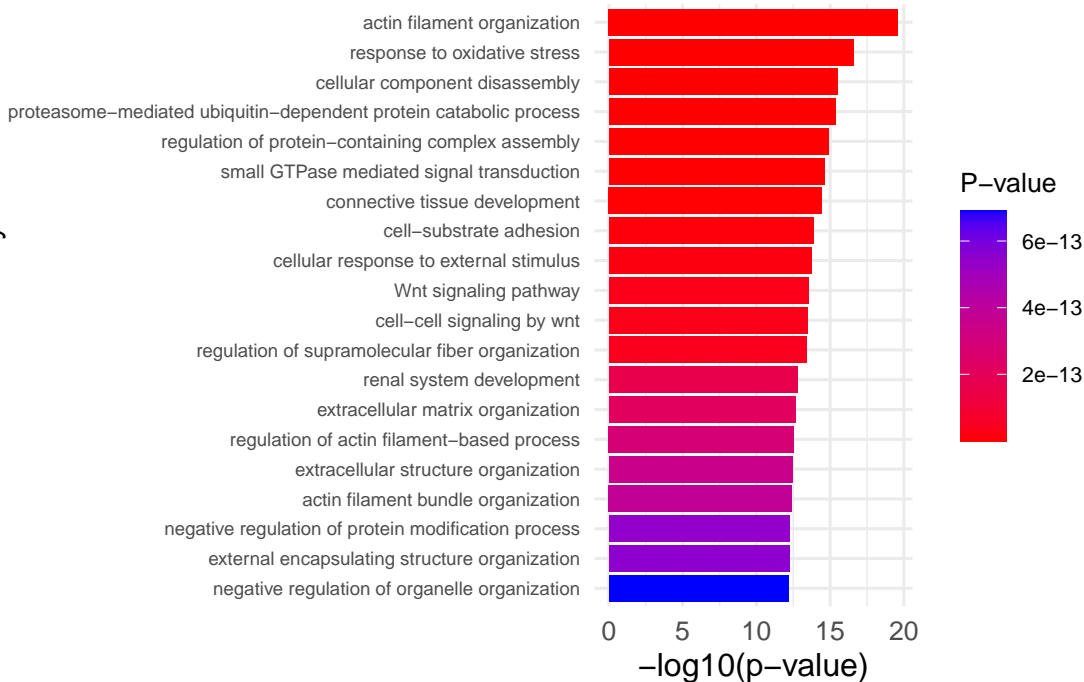

Supplement: Supplementary file 4 — Supplementary Pathway enrichment analysis [file 10456_2026_10045_MOESM4_ESM.zip › PathwayEnrichment analysis/hASC/Supplementary_GO_hASC_GFP_vs_L914F_Biological Process.pdf]

## Top GO Enriched Pathways (CC)

GO Pathways

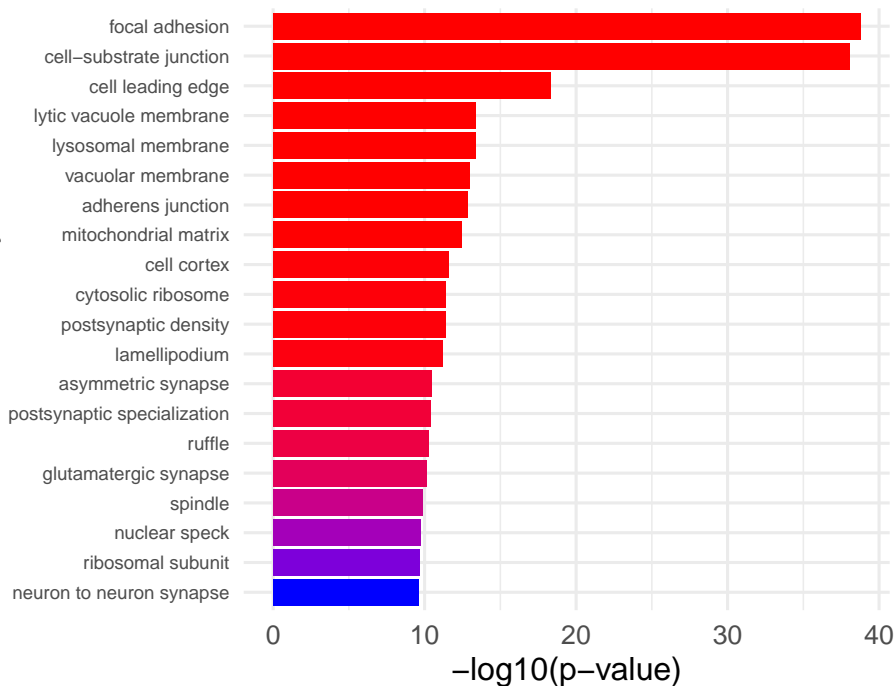

P-value

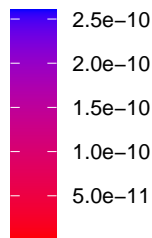

Supplement: Supplementary file 4 — Supplementary Pathway enrichment analysis [file 10456_2026_10045_MOESM4_ESM.zip › PathwayEnrichment analysis/hASC/Supplementary_GO_hASC_GFP_vs_L914F_Cellular Component.pdf]

# Top GO Enriched Pathways (MF)

GO Pathways

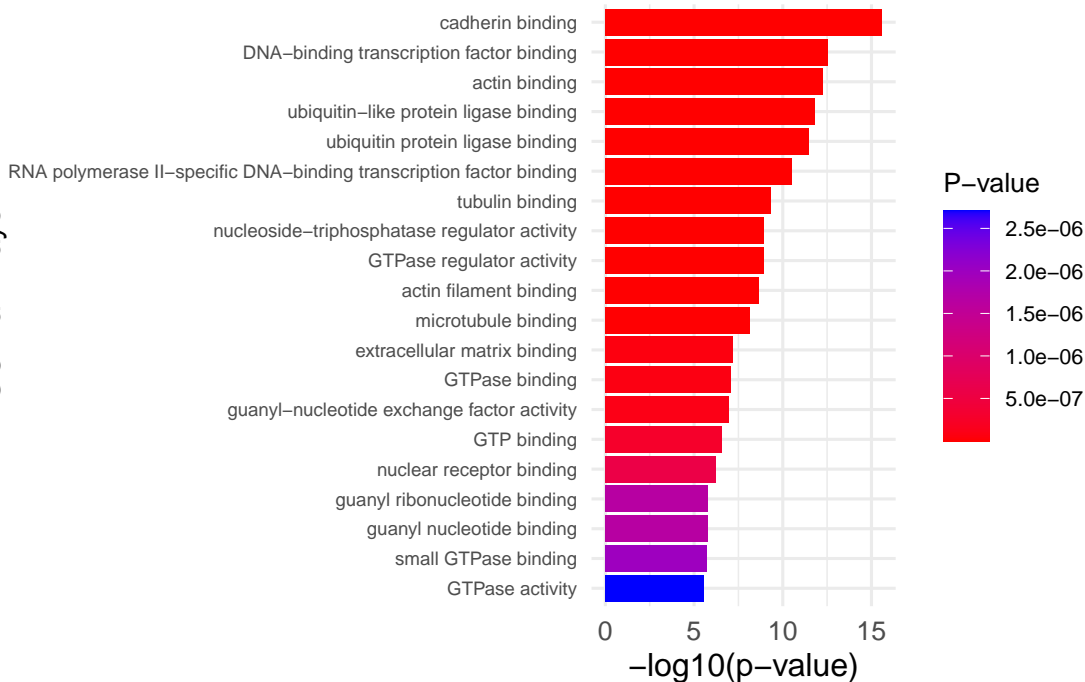

Supplement: Supplementary file 4 — Supplementary Pathway enrichment analysis [file 10456_2026_10045_MOESM4_ESM.zip › PathwayEnrichment analysis/hASC/Supplementary_GO_hASC_GFP_vs_L914F_Molecular Function.pdf]

# Top GO Enriched Pathways (BP)

GO Pathways

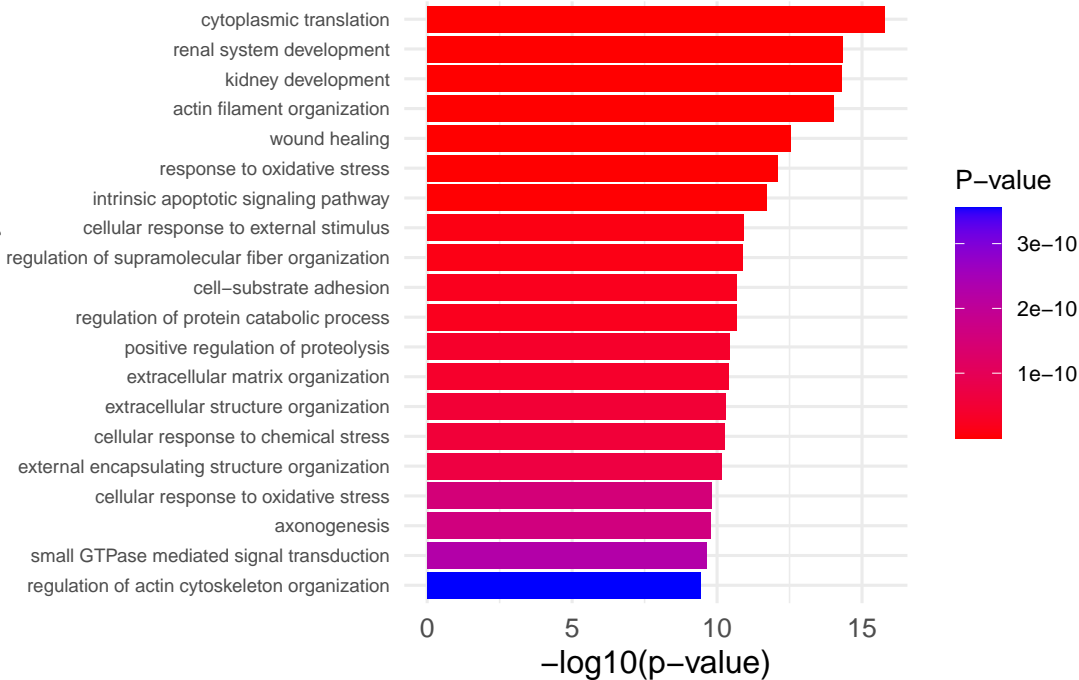

Supplement: Supplementary file 4 — Supplementary Pathway enrichment analysis [file 10456_2026_10045_MOESM4_ESM.zip › PathwayEnrichment analysis/hASC/Supplementary_GO_hASC_GFP_vs_WT_Biological Process.pdf]

# Top GO Enriched Pathways (CC)

GO Pathways

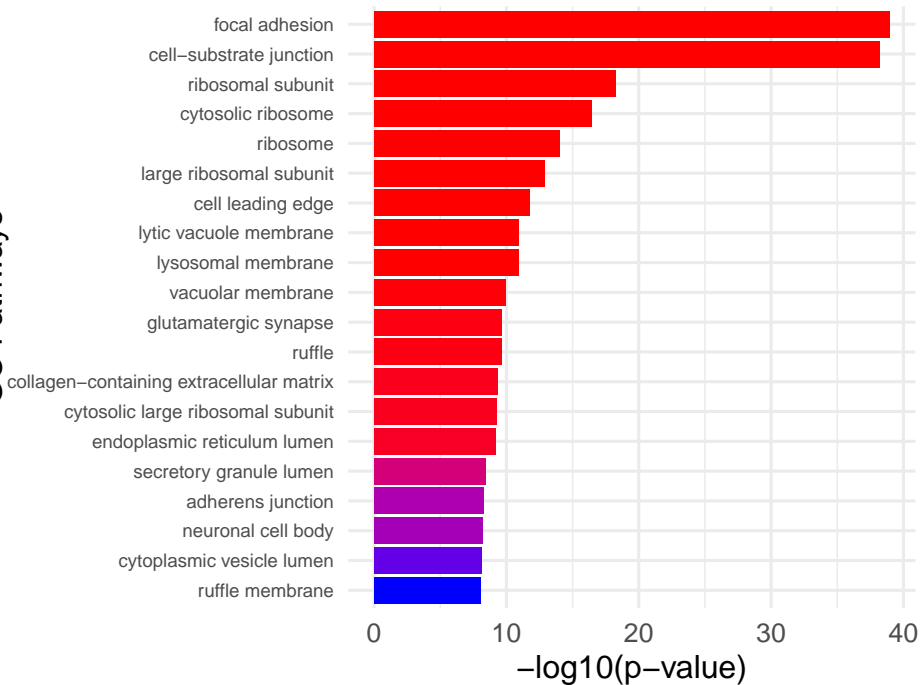

Supplement: Supplementary file 4 — Supplementary Pathway enrichment analysis [file 10456_2026_10045_MOESM4_ESM.zip › PathwayEnrichment analysis/hASC/Supplementary_GO_hASC_GFP_vs_WT_Cellular Component.pdf]

# Top GO Enriched Pathways (MF)

GO Pathways

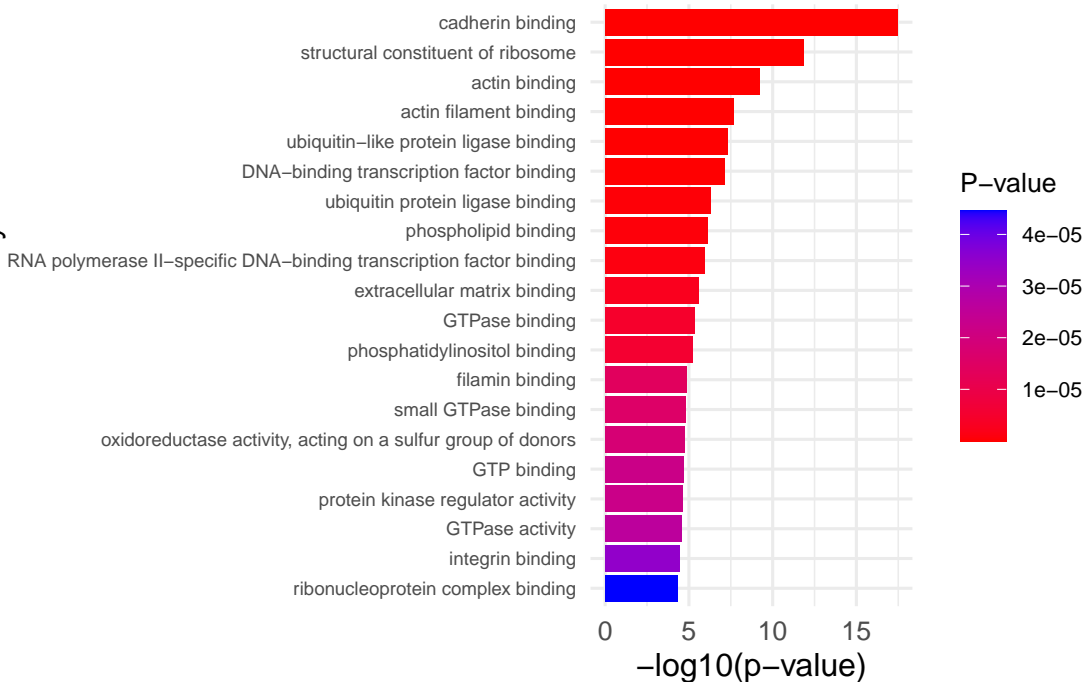

Supplement: Supplementary file 4 — Supplementary Pathway enrichment analysis [file 10456_2026_10045_MOESM4_ESM.zip › PathwayEnrichment analysis/hASC/Supplementary_GO_hASC_GFP_vs_WT_Molecular Function.pdf]

## Top GO Enriched Pathways (CC)

GO Pathways

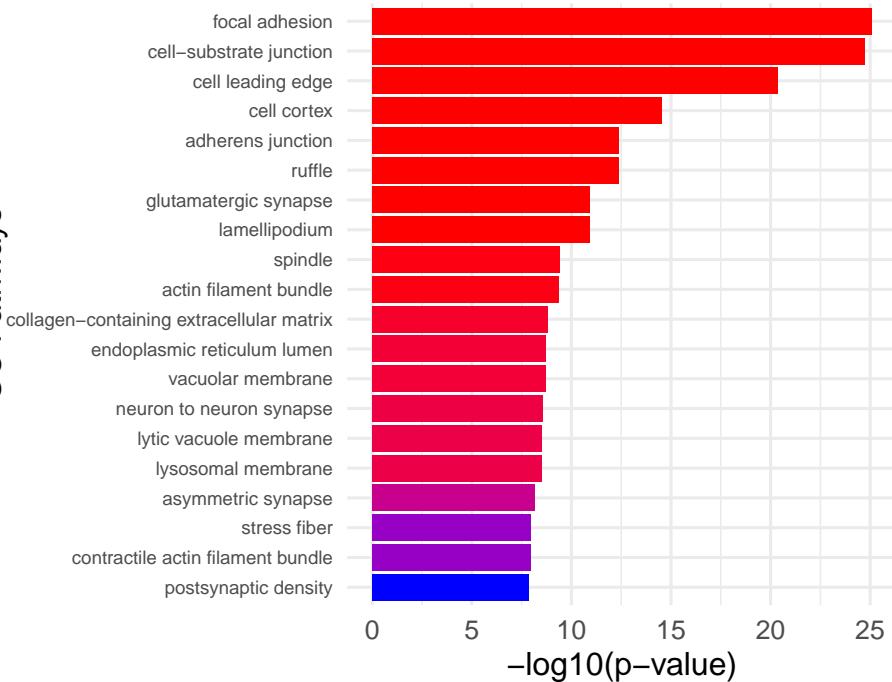

P-value

1.0e-08

5.0e-09

Supplement: Supplementary file 4 — Supplementary Pathway enrichment analysis [file 10456_2026_10045_MOESM4_ESM.zip › PathwayEnrichment analysis/hASC/Supplementary_GO_hASC_WT_vs_L914F_Cellular Component.pdf]

# Top GO Enriched Pathways (MF)

GO Pathways

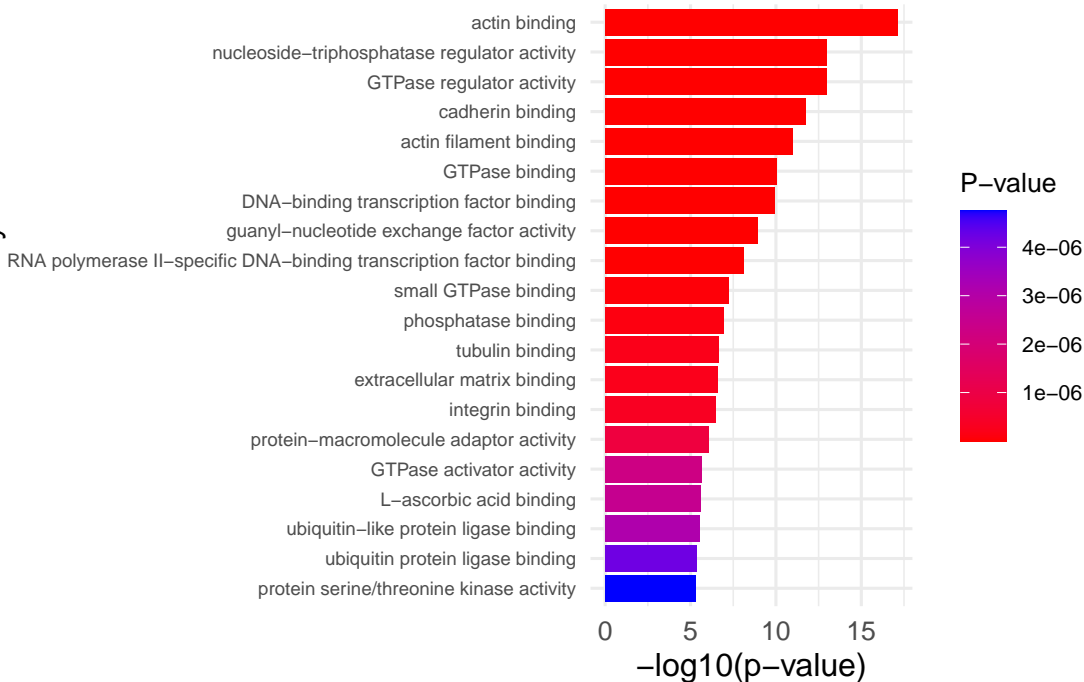

Supplement: Supplementary file 4 — Supplementary Pathway enrichment analysis [file 10456_2026_10045_MOESM4_ESM.zip › PathwayEnrichment analysis/hASC/Supplementary_GO_hASC_WT_vs_L914F_Molecular Function.pdf]

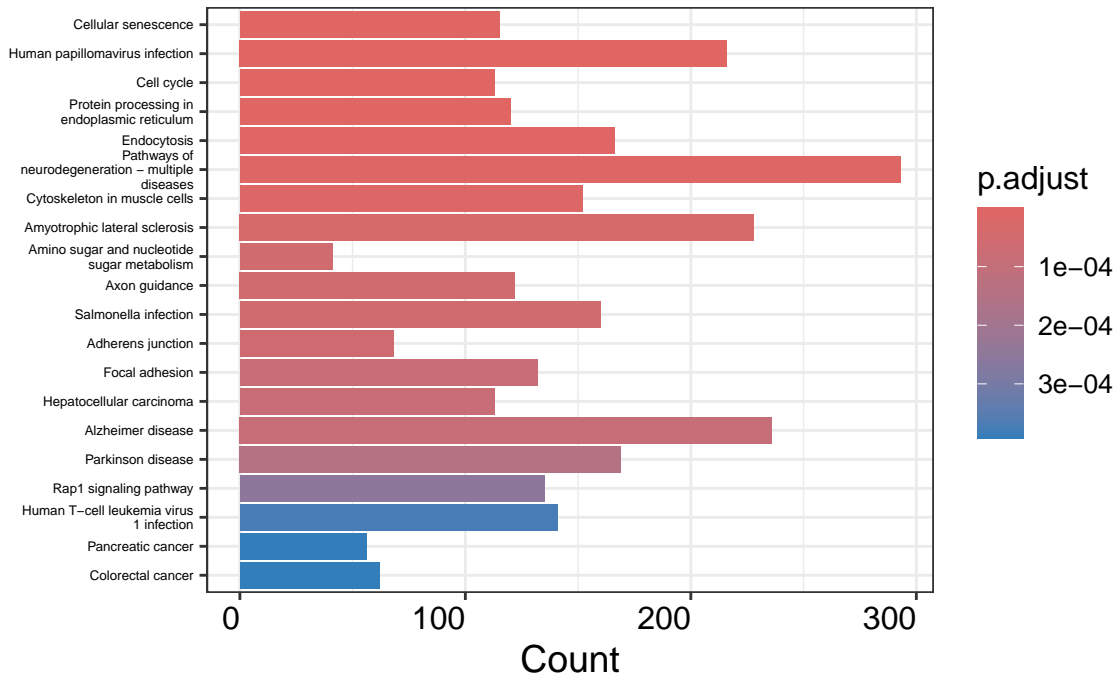

Supplement: Supplementary file 4 — Supplementary Pathway enrichment analysis [file 10456_2026_10045_MOESM4_ESM.zip › PathwayEnrichment analysis/hASC/Supplementary_KEGG_Barplot_hASC_GFP_vs_L914F.pdf]

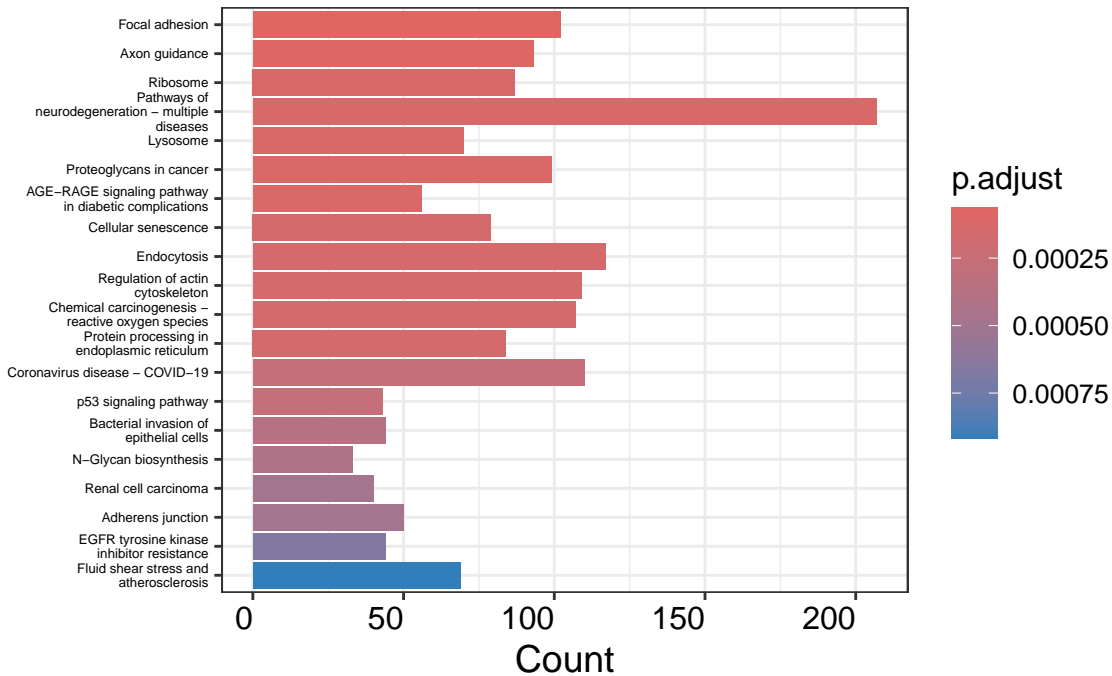

Supplement: Supplementary file 4 — Supplementary Pathway enrichment analysis [file 10456_2026_10045_MOESM4_ESM.zip › PathwayEnrichment analysis/hASC/Supplementary_KEGG_Barplot_hASC_GFP_vs_WT.pdf]

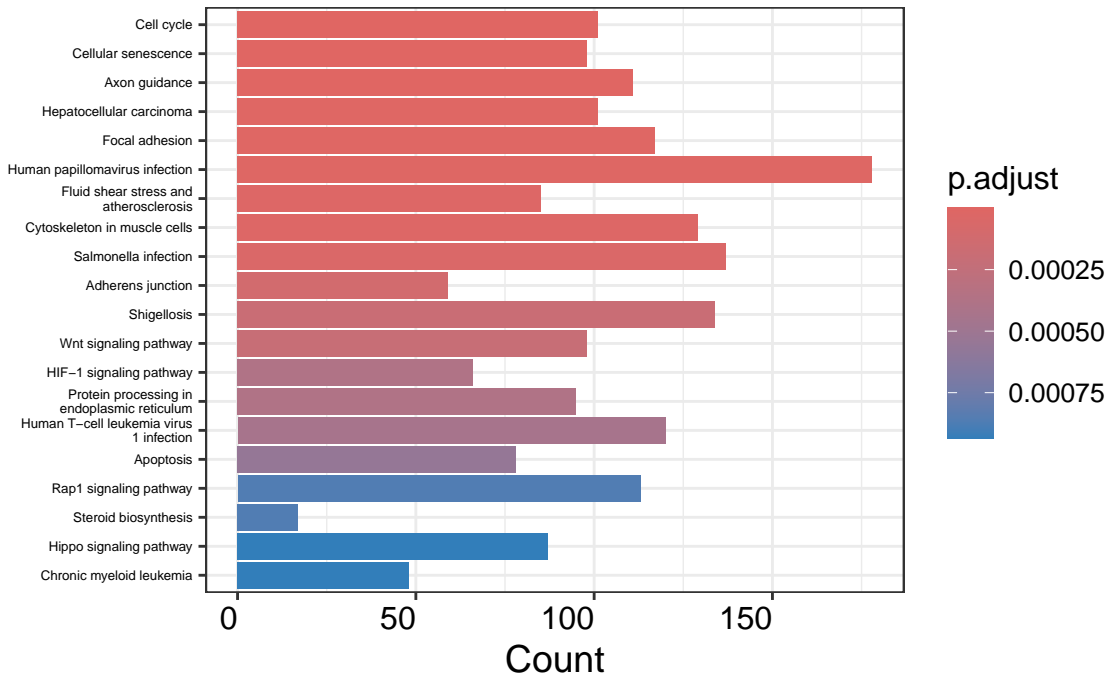

Supplement: Supplementary file 4 — Supplementary Pathway enrichment analysis [file 10456_2026_10045_MOESM4_ESM.zip › PathwayEnrichment analysis/hASC/Supplementary_KEGG_Barplot_hASC_WT_vs_L914F.pdf]

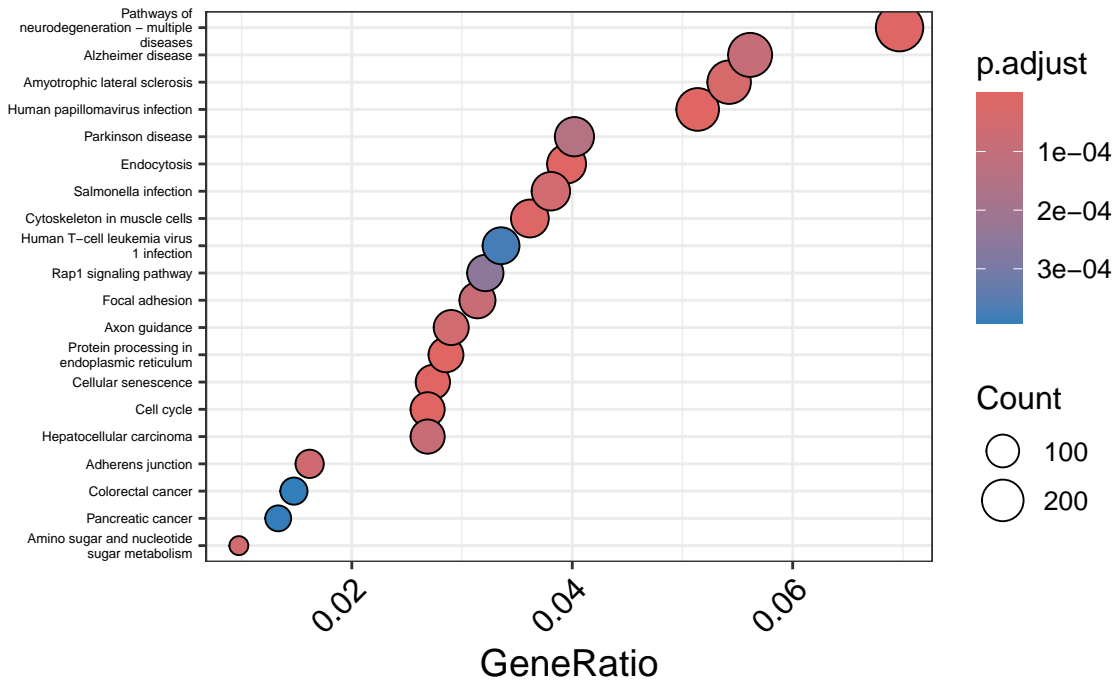

Supplement: Supplementary file 4 — Supplementary Pathway enrichment analysis [file 10456_2026_10045_MOESM4_ESM.zip › PathwayEnrichment analysis/hASC/Supplementary_KEGG_Dotplot_hASC_GFP_vs_L914F.pdf]

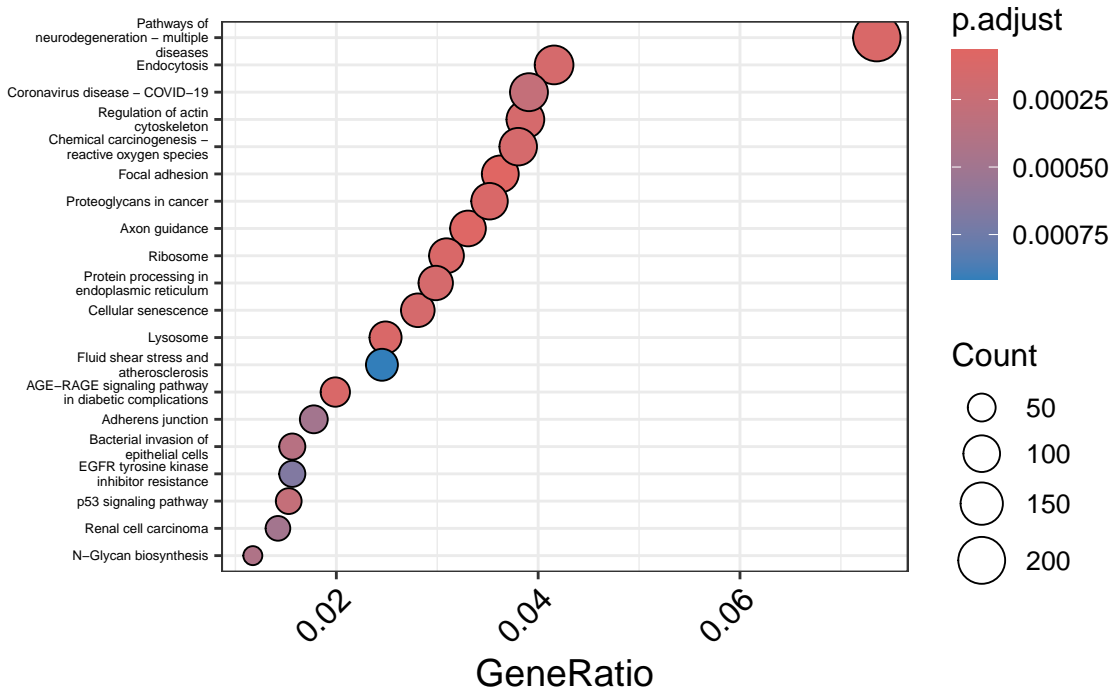

Supplement: Supplementary file 4 — Supplementary Pathway enrichment analysis [file 10456_2026_10045_MOESM4_ESM.zip › PathwayEnrichment analysis/hASC/Supplementary_KEGG_Dotplot_hASC_GFP_vs_WT.pdf]

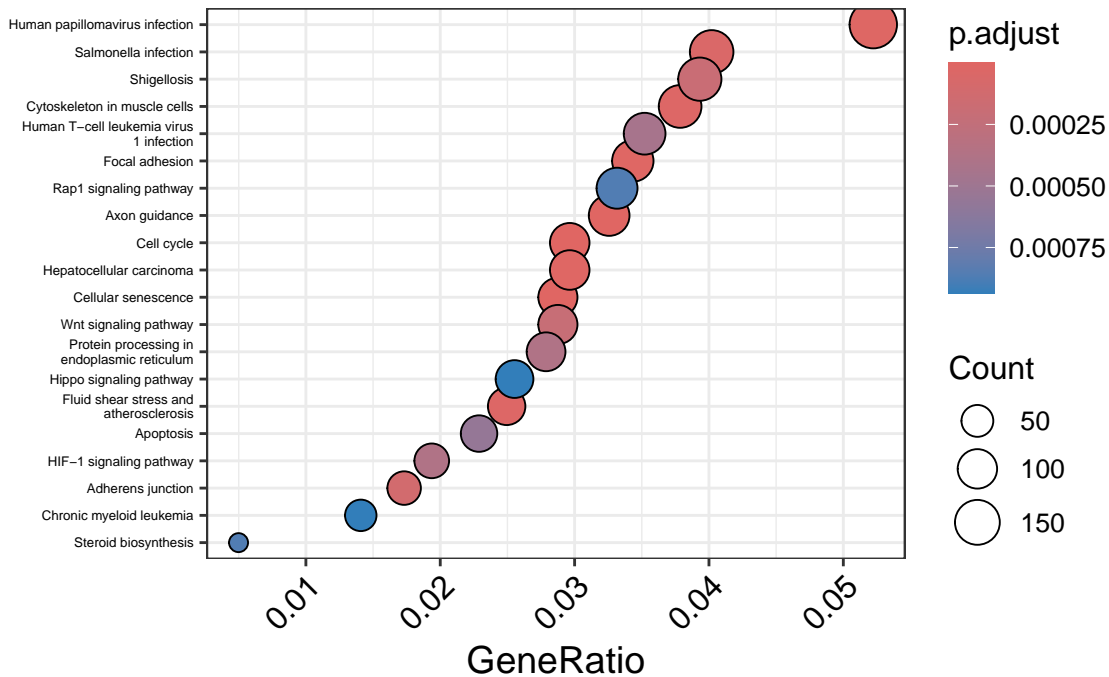

Supplement: Supplementary file 4 — Supplementary Pathway enrichment analysis [file 10456_2026_10045_MOESM4_ESM.zip › PathwayEnrichment analysis/hASC/Supplementary_KEGG_Dotplot_hASC_WT_vs_L914F.pdf]

## Top GO Enriched Pathways (BP)

GO Pathways

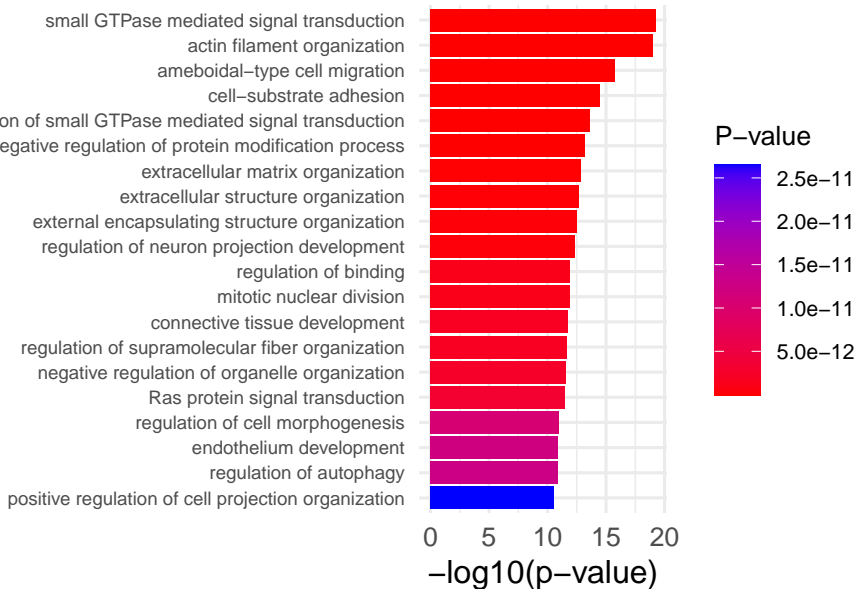

Supplement: Supplementary file 4 — Supplementary Pathway enrichment analysis [file 10456_2026_10045_MOESM4_ESM.zip › PathwayEnrichment analysis/HUVECs/Supplementary_GO_HUVECs_GFP_vs_L914F-Biological Process.pdf]

# Top GO Enriched Pathways (CC)

GO Pathways

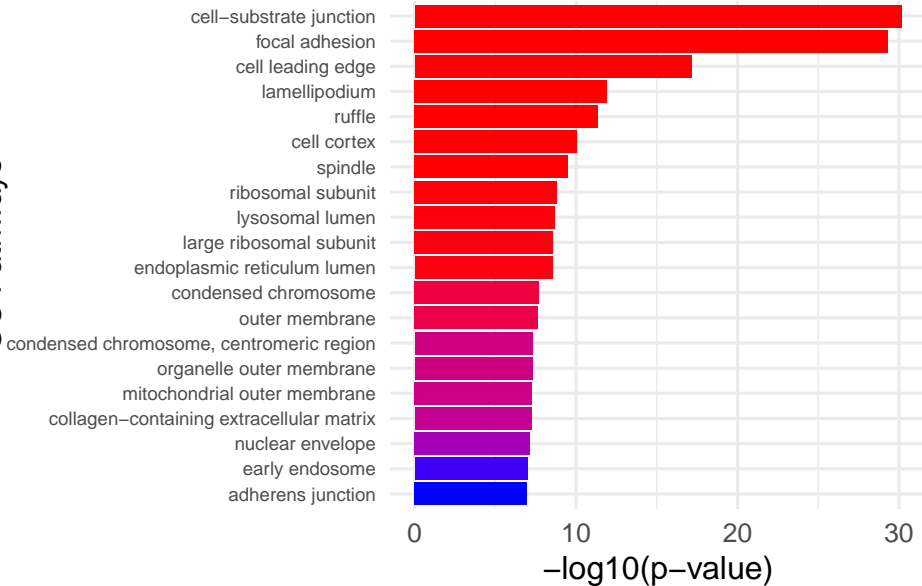

P-value

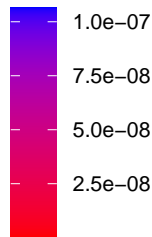

Supplement: Supplementary file 4 — Supplementary Pathway enrichment analysis [file 10456_2026_10045_MOESM4_ESM.zip › PathwayEnrichment analysis/HUVECs/Supplementary_GO_HUVECs_GFP_vs_L914F-Cellular Component.pdf]

# Top GO Enriched Pathways (MF)

GO Pathways

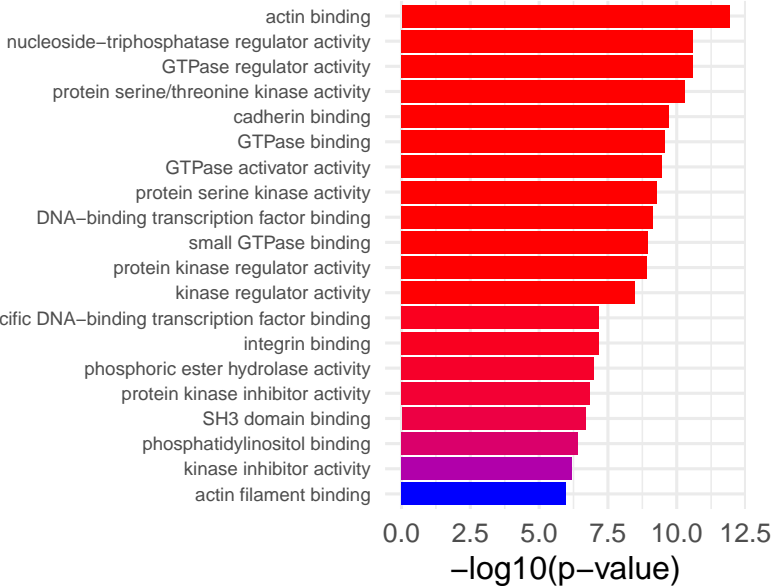

Supplement: Supplementary file 4 — Supplementary Pathway enrichment analysis [file 10456_2026_10045_MOESM4_ESM.zip › PathwayEnrichment analysis/HUVECs/Supplementary_GO_HUVECs_GFP_vs_L914F-Molecular Function.pdf]

## Top GO Enriched Pathways (MF)

GO Pathways

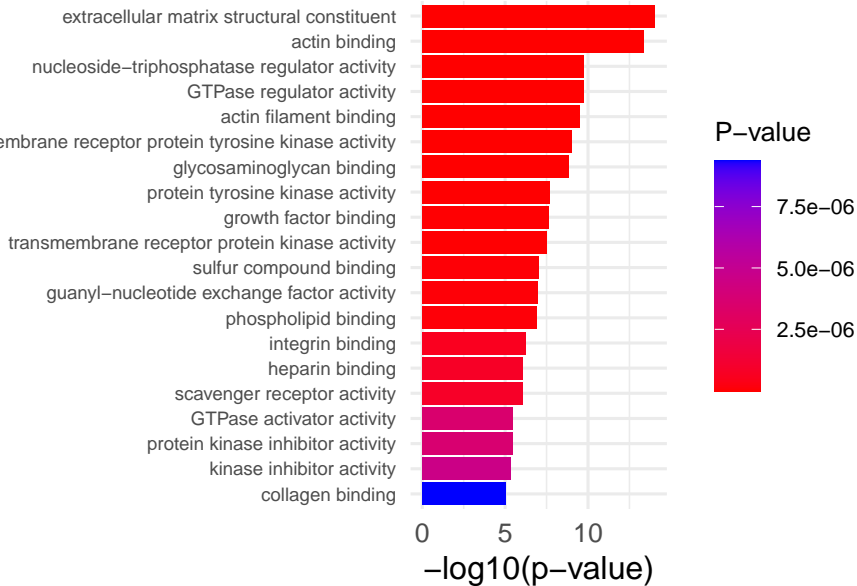

Supplement: Supplementary file 4 — Supplementary Pathway enrichment analysis [file 10456_2026_10045_MOESM4_ESM.zip › PathwayEnrichment analysis/HUVECs/Supplementary_GO_HUVECs_GFP_vs_WT-Molecular Function.pdf]

# Top GO Enriched Pathways (BP)

GO Pathways

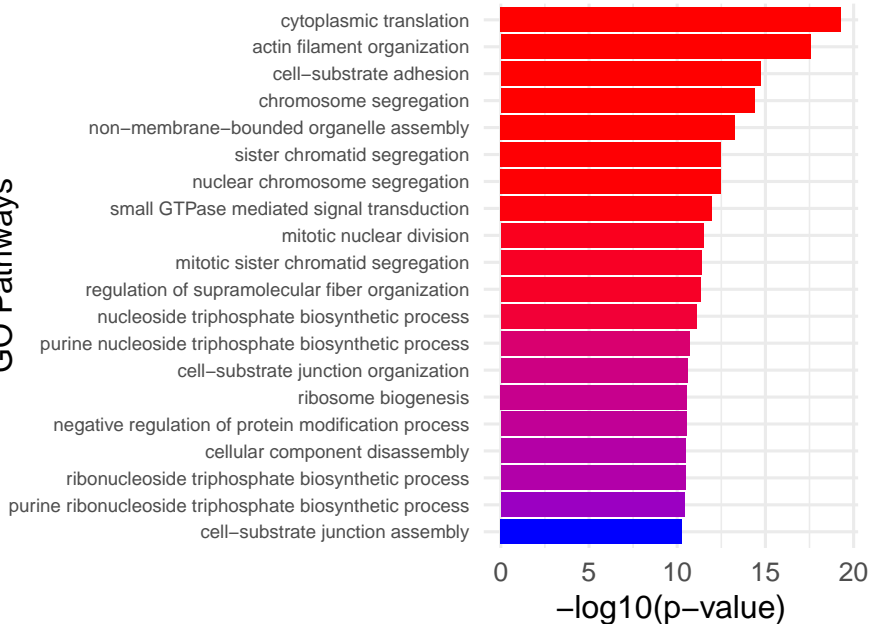

P-value

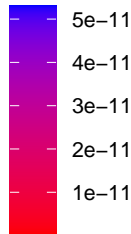

Supplement: Supplementary file 4 — Supplementary Pathway enrichment analysis [file 10456_2026_10045_MOESM4_ESM.zip › PathwayEnrichment analysis/HUVECs/Supplementary_GO_HUVECs_WT_vs_L914F-Biological Process.pdf]

## Top GO Enriched Pathways (CC)

GO Pathways

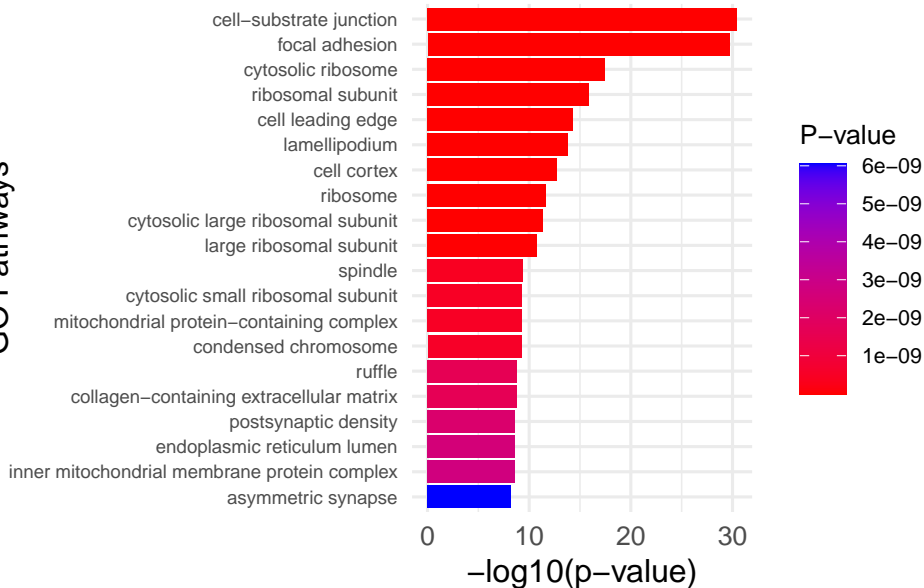

Supplement: Supplementary file 4 — Supplementary Pathway enrichment analysis [file 10456_2026_10045_MOESM4_ESM.zip › PathwayEnrichment analysis/HUVECs/Supplementary_GO_HUVECs_WT_vs_L914F-Cellular Component.pdf]

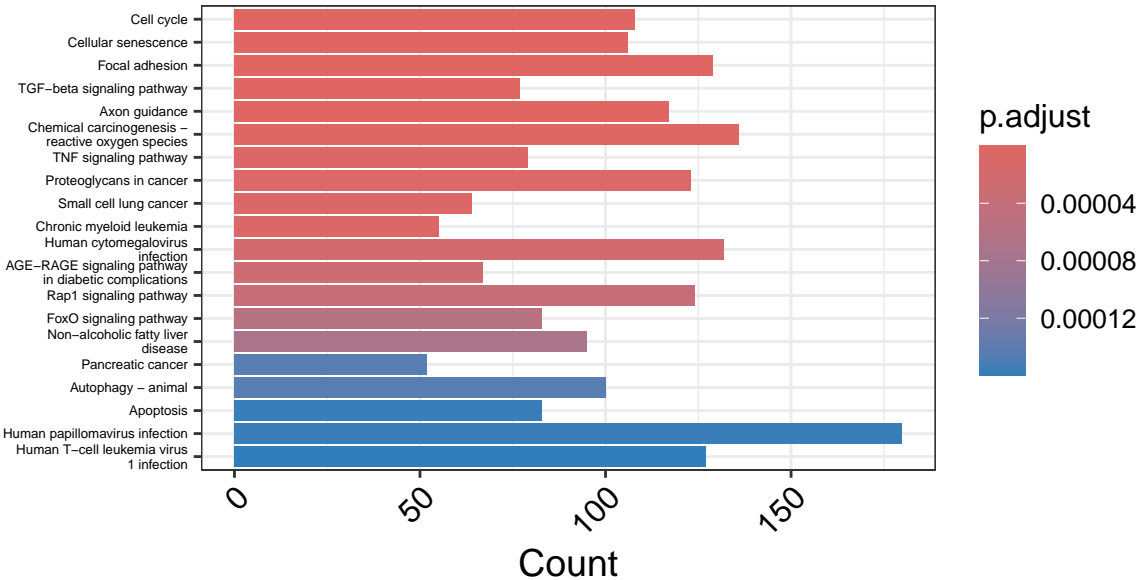

Supplement: Supplementary file 4 — Supplementary Pathway enrichment analysis [file 10456_2026_10045_MOESM4_ESM.zip › PathwayEnrichment analysis/HUVECs/Supplementary_KEGG_Barplot_HUVECs_GFP_vs_L914F.pdf]

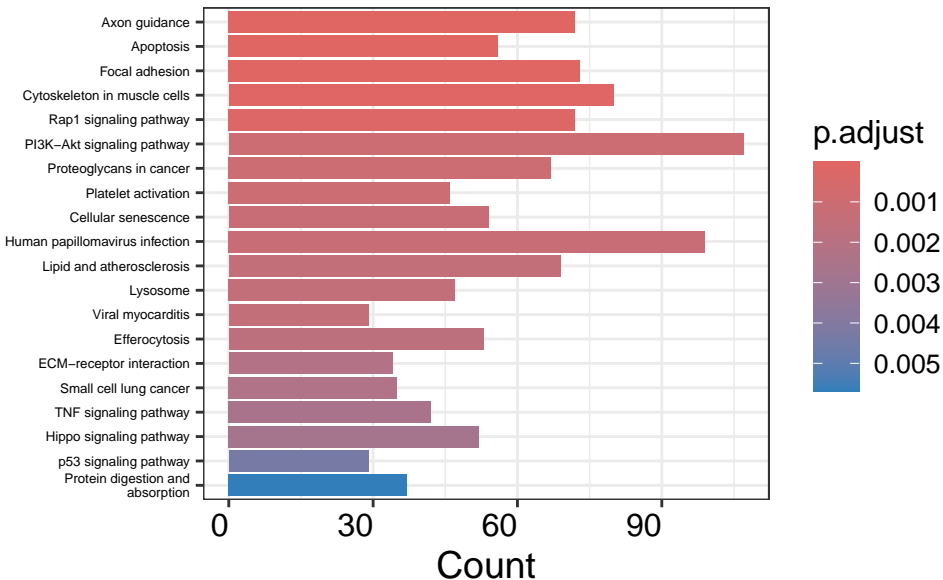

Supplement: Supplementary file 4 — Supplementary Pathway enrichment analysis [file 10456_2026_10045_MOESM4_ESM.zip › PathwayEnrichment analysis/HUVECs/Supplementary_KEGG_Barplot_HUVECs_GFP_vs_WT.pdf]

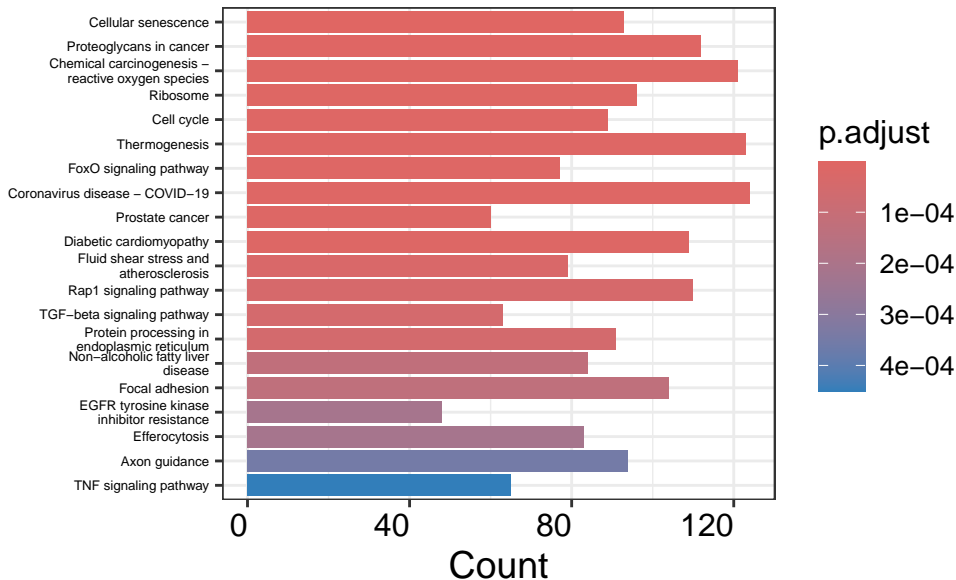

Supplement: Supplementary file 4 — Supplementary Pathway enrichment analysis [file 10456_2026_10045_MOESM4_ESM.zip › PathwayEnrichment analysis/HUVECs/Supplementary_KEGG_Barplot_HUVECs_WT_vs_L914F.pdf]

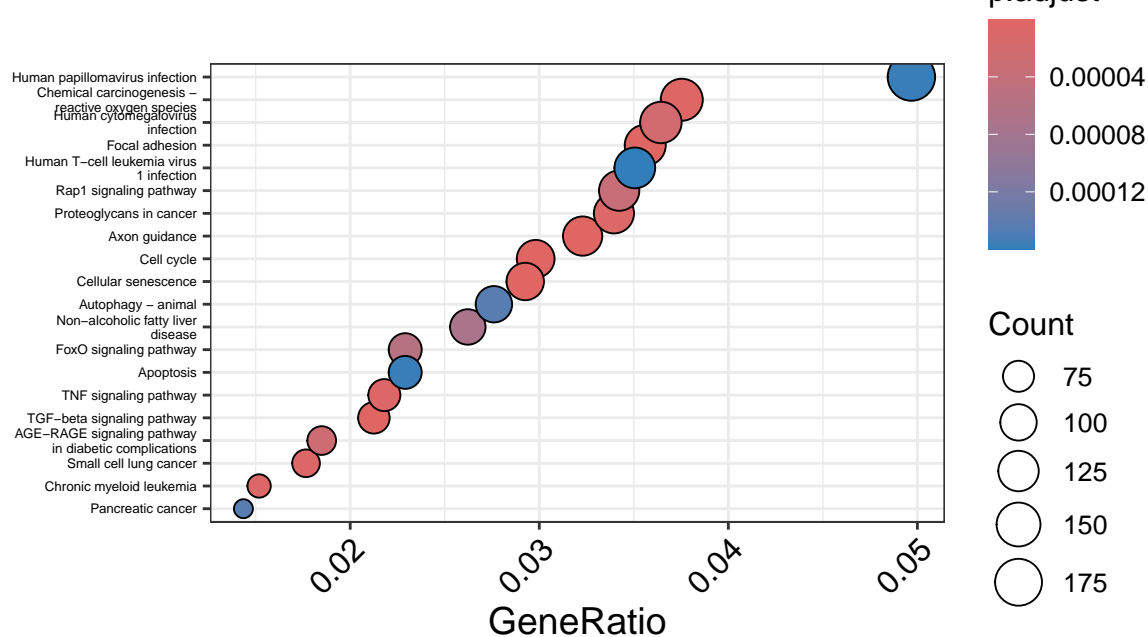

Supplement: Supplementary file 4 — Supplementary Pathway enrichment analysis [file 10456_2026_10045_MOESM4_ESM.zip › PathwayEnrichment analysis/HUVECs/Supplementary_KEGG_Dotplot_HUVECs_GFP_vs_L914F.pdf]

p.adjust

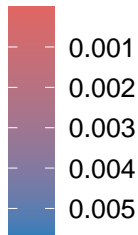

Count

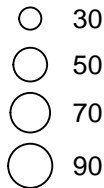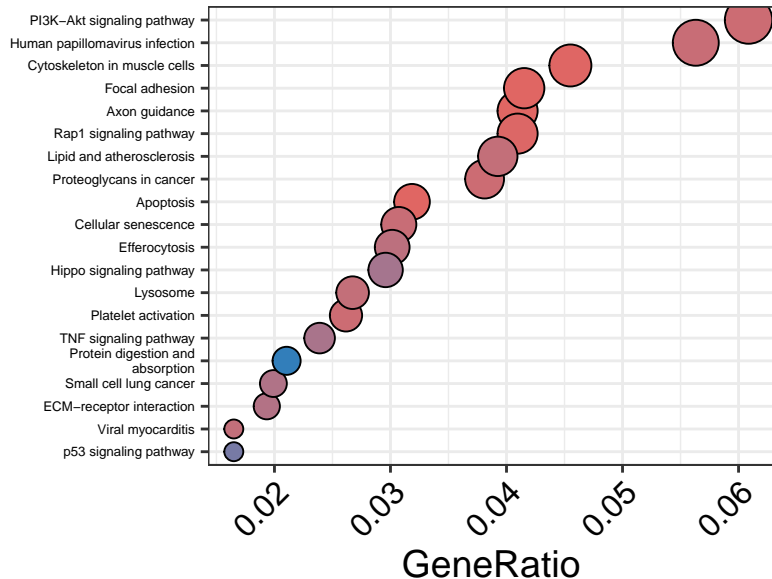

Supplement: Supplementary file 4 — Supplementary Pathway enrichment analysis [file 10456_2026_10045_MOESM4_ESM.zip › PathwayEnrichment analysis/HUVECs/Supplementary_KEGG_Dotplot_HUVECs_GFP_vs_WT.pdf]

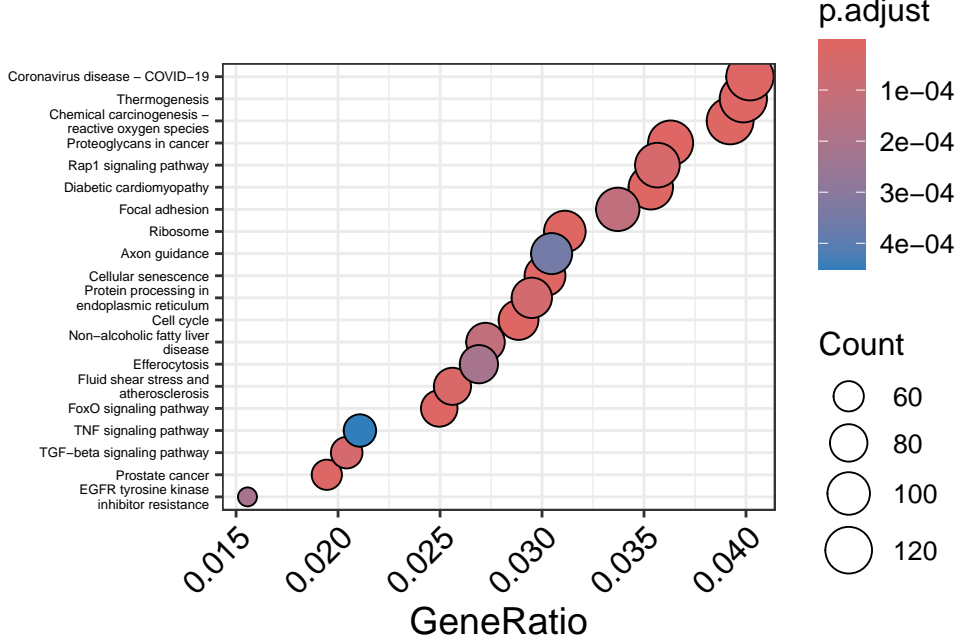

Supplement: Supplementary file 4 — Supplementary Pathway enrichment analysis [file 10456_2026_10045_MOESM4_ESM.zip › PathwayEnrichment analysis/HUVECs/Supplementary_KEGG_Dotplot_HUVECs_WT_vs_L914F.pdf]

## Top GO Enriched Pathways (BP)

GO Pathways

P-value

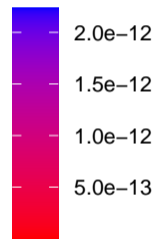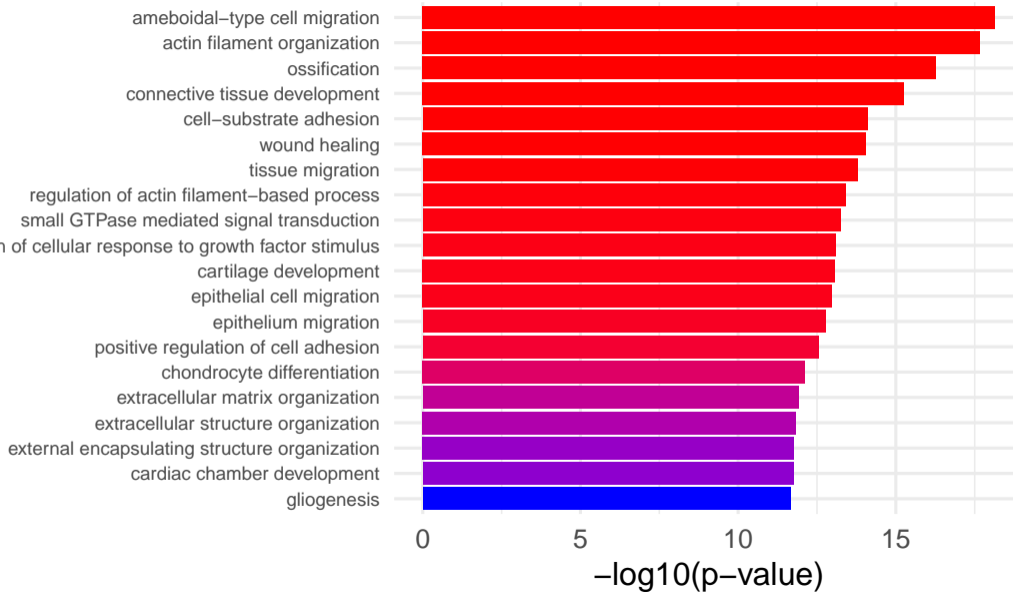

Supplement: Supplementary file 4 — Supplementary Pathway enrichment analysis [file 10456_2026_10045_MOESM4_ESM.zip › PathwayEnrichment analysis/SMC/Supplementary_GO_day2_SMC_WT_vs_L914F_Biological Processes.pdf]

## Top GO Enriched Pathways (CC)

GO Pathways

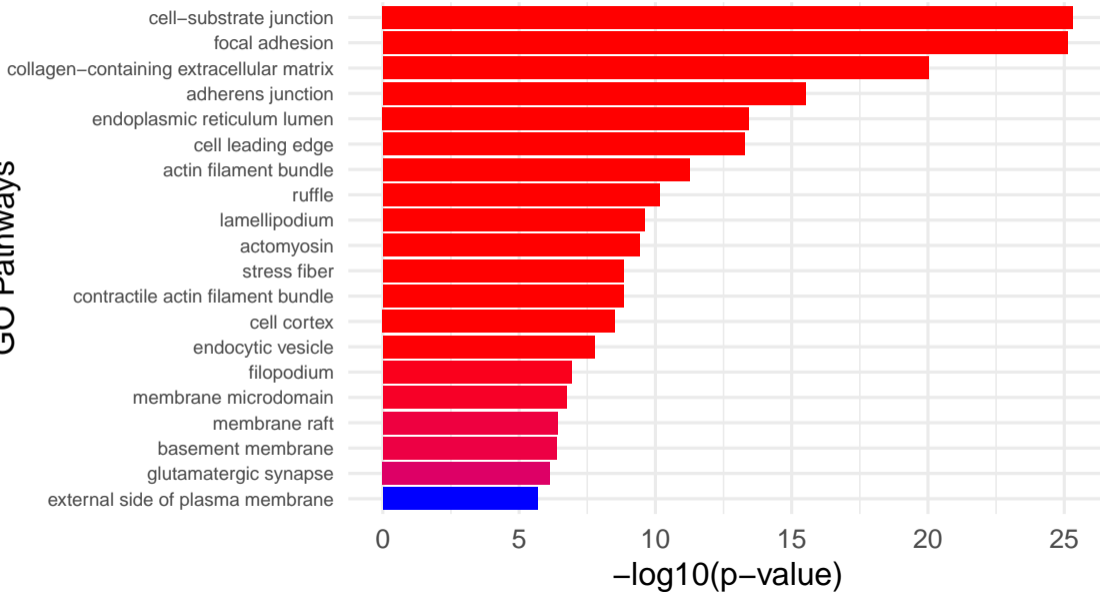

P-value

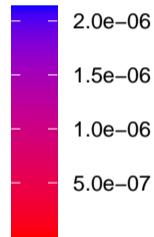

Supplement: Supplementary file 4 — Supplementary Pathway enrichment analysis [file 10456_2026_10045_MOESM4_ESM.zip › PathwayEnrichment analysis/SMC/Supplementary_GO_day2_SMC_WT_vs_L914F_Cellular Components.pdf]

## Top GO Enriched Pathways (MF)

GO Pathways

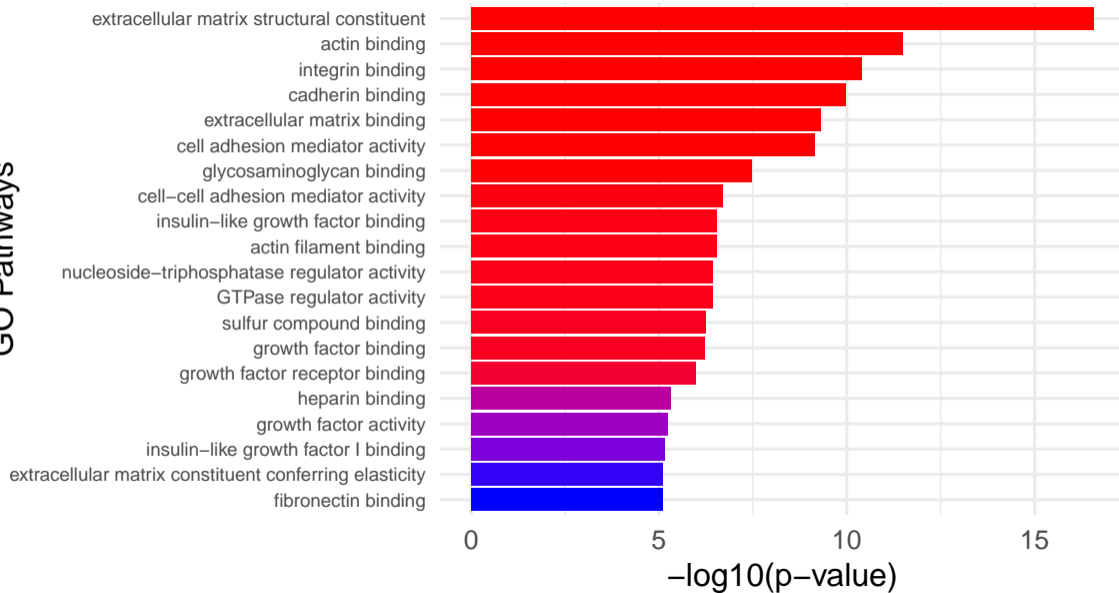

P-value

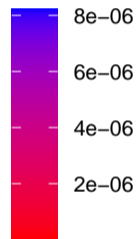

Supplement: Supplementary file 4 — Supplementary Pathway enrichment analysis [file 10456_2026_10045_MOESM4_ESM.zip › PathwayEnrichment analysis/SMC/Supplementary_GO_day2_SMC_WT_vs_L914F_Molecular Functions.pdf]

# Top GO Enriched Pathways (BP)

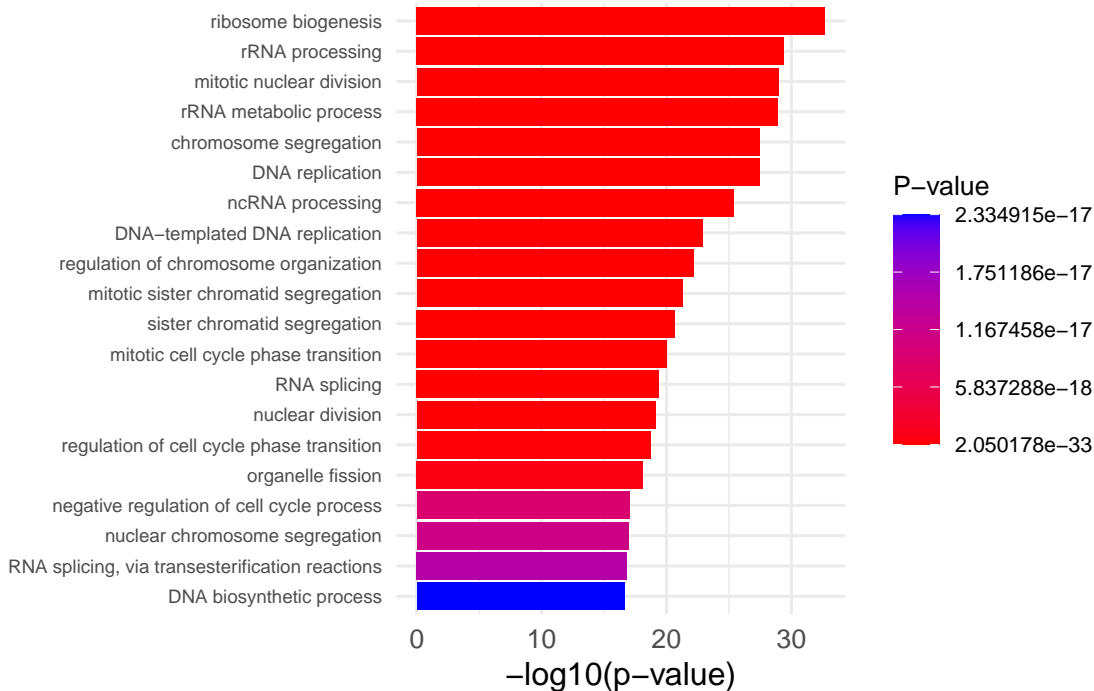

Supplement: Supplementary file 4 — Supplementary Pathway enrichment analysis [file 10456_2026_10045_MOESM4_ESM.zip › PathwayEnrichment analysis/SMC/Supplementary_GO_day4_SMC_WT_vs_L914F_Biological Processes.pdf]

# Top GO Enriched Pathways (CC)

GO Pathways

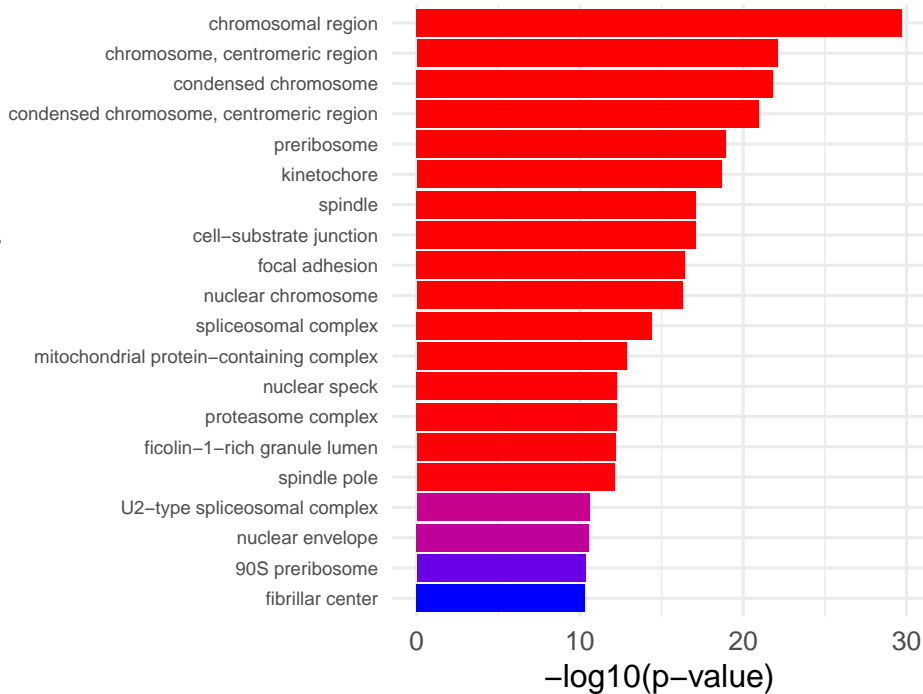

P-value

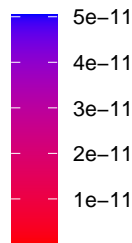

Supplement: Supplementary file 4 — Supplementary Pathway enrichment analysis [file 10456_2026_10045_MOESM4_ESM.zip › PathwayEnrichment analysis/SMC/Supplementary_GO_day4_SMC_WT_vs_L914F_Cellular Components.pdf]

# Top GO Enriched Pathways (MF)

GO Pathways

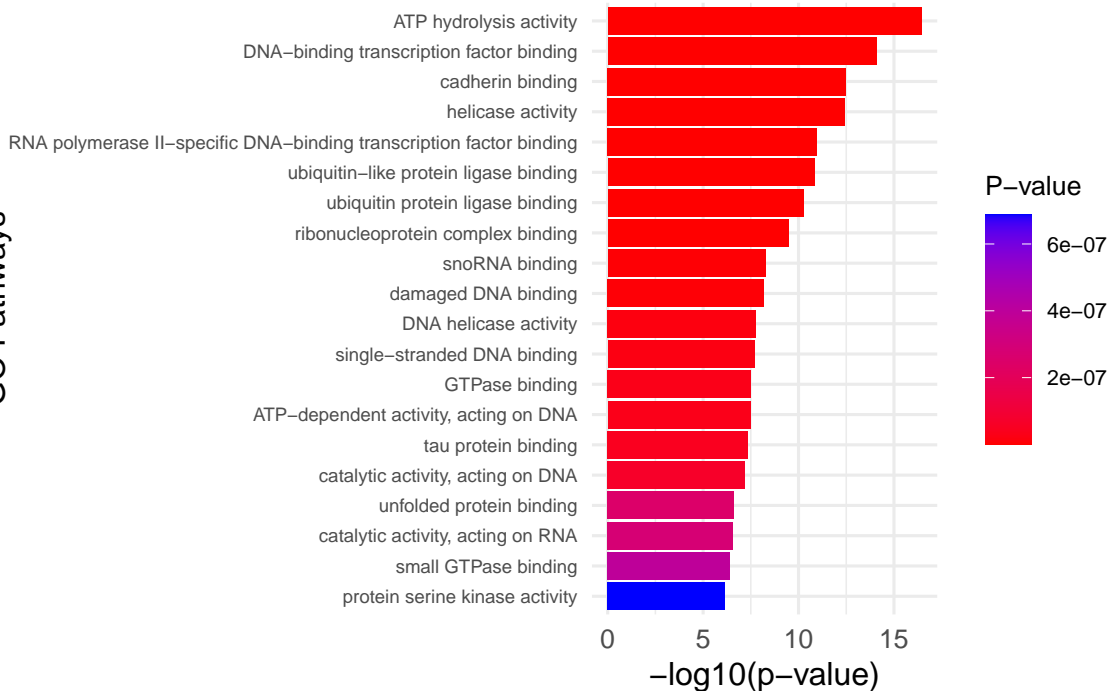

Supplement: Supplementary file 4 — Supplementary Pathway enrichment analysis [file 10456_2026_10045_MOESM4_ESM.zip › PathwayEnrichment analysis/SMC/Supplementary_GO_day4_SMC_WT_vs_L914F_Molecular Functions.pdf]

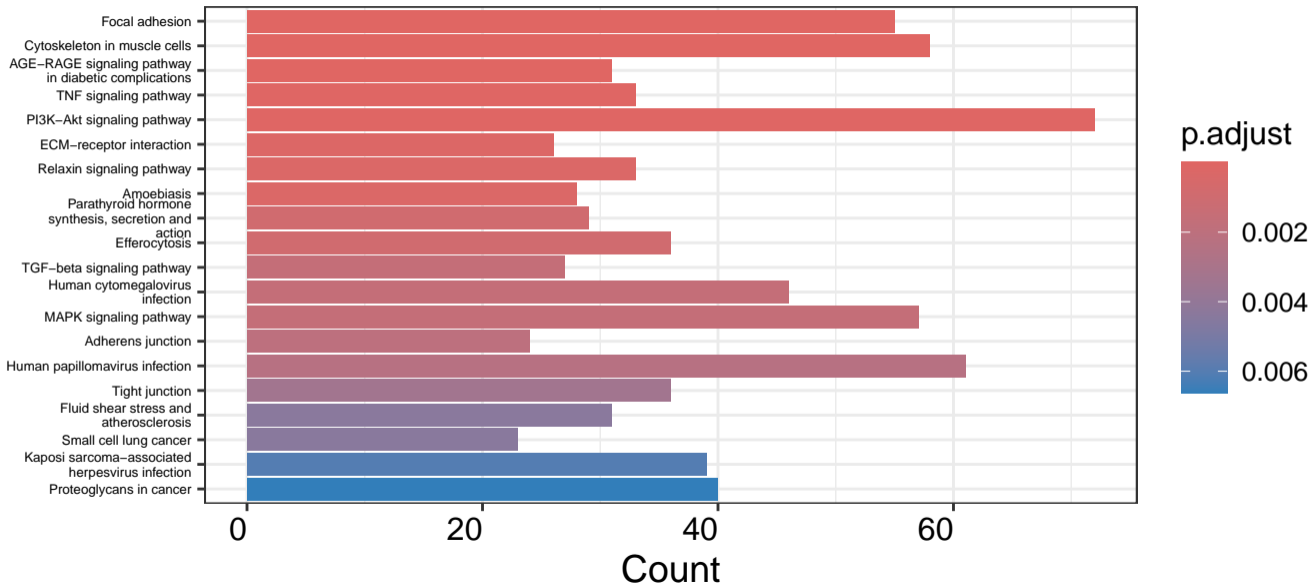

Supplement: Supplementary file 4 — Supplementary Pathway enrichment analysis [file 10456_2026_10045_MOESM4_ESM.zip › PathwayEnrichment analysis/SMC/Supplementary_KEGG_Barplot_day2_SMC_WT_vs_L914F.pdf]

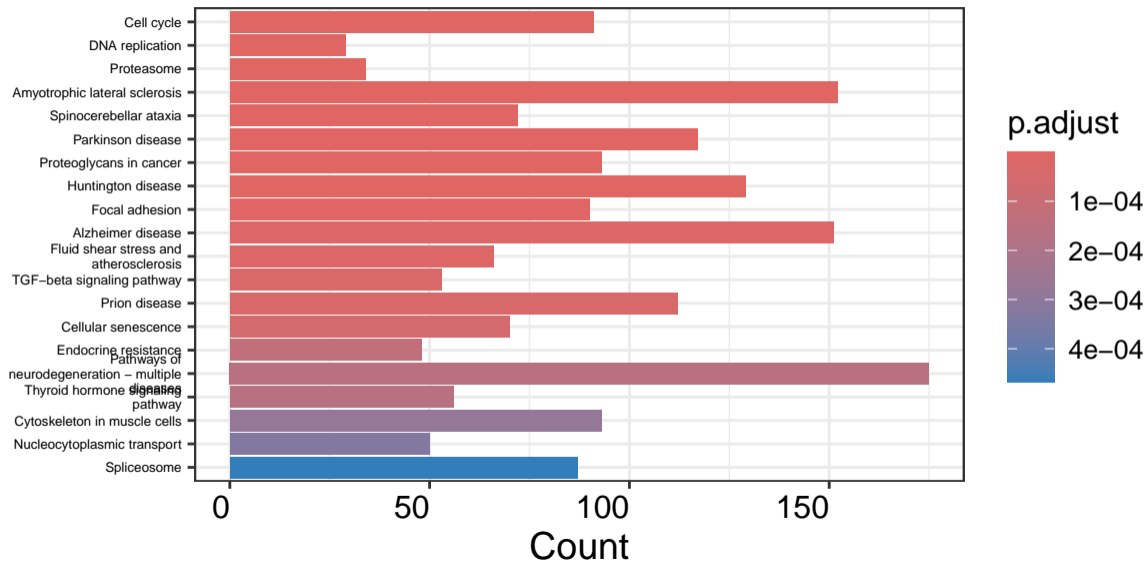

Supplement: Supplementary file 4 — Supplementary Pathway enrichment analysis [file 10456_2026_10045_MOESM4_ESM.zip › PathwayEnrichment analysis/SMC/Supplementary_KEGG_Barplot_day4_SMC_WT_vs_L914F.pdf]

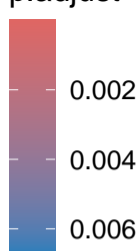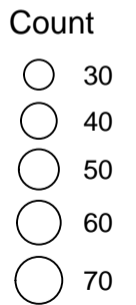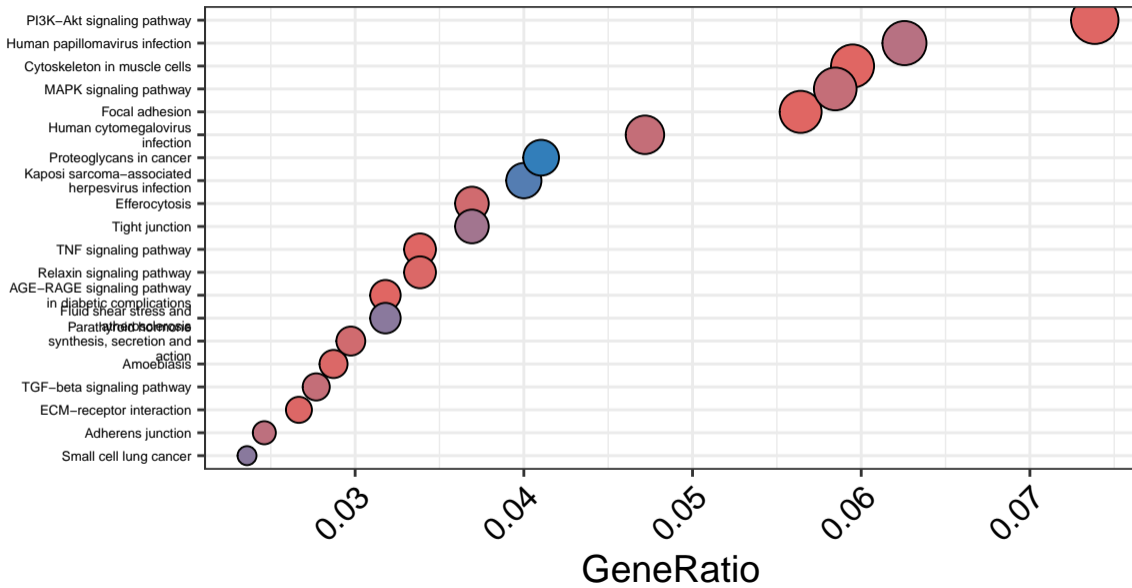

Supplement: Supplementary file 4 — Supplementary Pathway enrichment analysis [file 10456_2026_10045_MOESM4_ESM.zip › PathwayEnrichment analysis/SMC/Supplementary_KEGG_Dotplot_day2_SMC_WT_L914F.pdf]

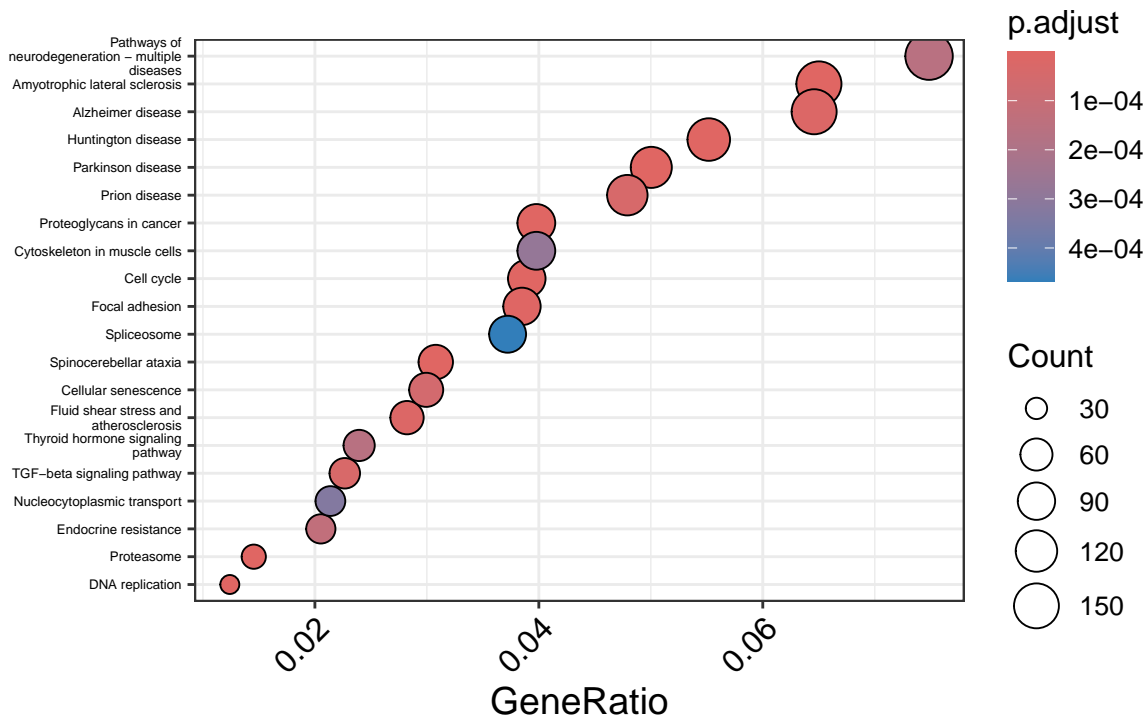

Supplement: Supplementary file 4 — Supplementary Pathway enrichment analysis [file 10456_2026_10045_MOESM4_ESM.zip › PathwayEnrichment analysis/SMC/Supplementary_KEGG_Dotplot_day4_SMC_WT_L914F.pdf]
